# Plastic ingestion by harbour porpoises *Phocoena phocoena* in the Netherlands: Establishing a standardised method

**DOI:** 10.1007/s13280-017-1002-y

**Published:** 2018-01-05

**Authors:** Jan A. van Franeker, Elisa L. Bravo Rebolledo, Eileen Hesse, Lonneke L. IJsseldijk, Susanne Kühn, Mardik Leopold, Lara Mielke

**Affiliations:** 1 WUR, Wageningen Marine Research, Ankerpark 27, 1781 AG Den Helder, The Netherlands; 2 Elisa Bravo - Ecological and Biological Research, Bilthoven, The Netherlands; 3 Department of Pathobiology, Faculty of Veterinary Medicine, Utrecht University, Yalelaan 1, 3584 CL Utrecht, The Netherlands; 4 4101 CK Culemborg, The Netherlands; 5 58640 Iserlohn, Germany; 6 24329 Goernitz, Germany

**Keywords:** Harbour porpoise, Marine litter monitoring, Marine strategy framework directive (MSFD), North Sea, *Phocoena phocoena*, Plastic ingestion

## Abstract

Stomach contents of harbour porpoises (*Phocoena phocoena*) collected in the Netherlands between 2003 and 2013 were inspected for the presence of plastic and other man-made litter. In 654 stomach samples the frequency of occurrence of plastic litter was 7% with less than 0.5% additional presence of non-synthetic man-made litter. However, we show that when a dedicated standard protocol for the detection of litter is followed, a considerably higher percentage (15% of 81 harbour porpoise stomachs from the period 2010–2013) contained plastic litter. Results thus strongly depended on methods used and time period considered. Occurrence of litter in the stomach was correlated to the presence of other non-food remains like stones, shells, bog-wood, etc., suggesting that litter was often ingested accidentally when the animals foraged close to the bottom. Most items were small and were not considered to have had a major health impact. No evident differences in ingestion were found between sexes or age groups, with the exception that neonates contained no litter. Polyethylene and polypropylene were the most common plastic types encountered. Compared to earlier literature on the harbour porpoise and related species, our results suggest higher levels of ingestion of litter. This is largely due to the lack of dedicated protocols to investigate marine litter ingestion in previous studies. Still, the low frequency of ingestion, and minor number and mass of litter items found in harbour porpoises in the relatively polluted southern North Sea indicates that the species is not a strong candidate for annual monitoring of marine litter trends under the EU marine strategy framework directive. However, for longer-term comparisons and regional differences, with proper dedicated protocols applied, the harbour porpoise has specific use in quantifying litter presence in the, for that specific objective, poorly studied benthic marine habitat.

**Electronic supplementary material:**

The online version of this article (10.1007/s13280-017-1002-y) contains supplementary material, which is available to authorized users.

## Introduction

The wide distribution and abundance of man-made litter, in particular plastics, has been signalled as a major threat to the oceans (UNEP 2011, 2014; CBD 2016) which affects a broad range of marine organisms through entanglement and ingestion (Kühn et al. 2015). Marine litter has long been an important issue in the Oslo-Paris Convention for the Protection of the Marine Environment of the North-East Atlantic (OSPAR) and more recently has become strongly embedded also in European Union (EU) policy. In the marine strategy framework directive (MSFD), the European Commission (EC) calls on its member states to achieve a ‘Good Environmental Status’ (GES) in all European seas by the year 2020 (EC 2008, 2010). Among the many environmental aspects of MSFD aiming at ecological conservation and sustainable use of marine resources, ‘Descriptor 10’ addresses marine litter, in which GES is defined as the situation where *“marine litter does not cause harm to the coastal and marine environment”*. How this should be interpreted and dealt with in terms of assessments is being addressed further by a specialist group (Galgani et al. 2010; MSFD-TSGML 2011, 2013; Werner et al. 2016), and regional planning in order to achieve GES is underway (e.g. OSPAR 2014).

An important aspect of ‘harm’ from marine litter is the ingestion of plastic objects by marine organisms. Ingestion of plastics is widespread among marine wildlife (Kühn et al. 2015) and the monitoring of ingestion levels is one of the policy approaches to assess the current situation and identify targets for future GES. In and around the North Sea, the northern fulmar *(Fulmarus glacialis)* has been selected as the principal monitoring species, using stomach contents of bird corpses that beached or accidentally died in collisions or as fisheries bycatch. Standard study methods have been published in Van Franeker et al. (2011), with specific policy monitoring requirements detailed in OSPAR (2015). In the Mediterranean, the loggerhead turtle (*Caretta caretta)* has been selected as the major monitoring species (Matiddi et al. 2017). In the European approach, where possible, additional studies of plastic ingestion among a diverse range of marine wildlife, including fish and marine mammals are recommended (MSFD-TSGML 2013; Galgani et al. 2014).

Within the Dutch sector of the North Sea, in addition to the long-term monitoring of plastic ingestion by fulmars (Van Franeker et al. 2011; Van Franeker and Law 2015), studies have been conducted on plastic ingestion by harbour seals (*Phoca vitulina)* (Bravo Rebolledo et al. 2013), fishes (Foekema et al. 2013) and whales (Besseling et al. 2015; Bravo Rebolledo et al. 2016; Unger et al. 2016). For harbour porpoises, except for single cases (Bosch 1950; Kastelein and Lavaleije 1992), no systematic data on litter ingestion in Dutch coastal waters were collected. Reports on harbour porpoises found dead in neighbouring countries suggested very low rates or absence of litter in the stomach (De Pierrepont et al. 2005; Deaville et al. 2010; Haelters et al. 2012; Unger et al. 2017). However, for Dutch harbour porpoises, a preliminary survey of non-food items listed in the database developed for diet studies (Leopold 2015) indicated that litter ingestion was in fact more common. In that diet study, after initial general inspection, the stomach contents had been cleaned under running water in a glass beaker in order to collect identifiable hard prey items (e.g. otoliths, bones, jaws) at the bottom, with lighter prey tissue remains flowing out. In this method, the residues remaining inside the beaker were carefully inspected with a binocular microscope, but the outflowing water was not controlled in detail for small objects. Microplastics (defined as smaller than 5 mm), but also larger transparent items might have passed unnoticed. To improve and standardise detection of non-food objects such as plastic, a start was made to collect all outflowing material over a 1 mm sieve using a subsample of stomachs that had not yet been processed. The current study aims to evaluate the added value of such a dedicated method for the assessment of plastic ingestion by the harbour porpoise, and to provide a baseline for plastic ingestion by this species in the Netherlands. Results can elucidate the potential of this species for more regular monitoring of marine litter in the area in relation to GES under the MSFD.

## Materials and Methods

### Post-mortem investigation

Beached harbour porpoises were collected for post-mortem investigation by the Dutch voluntary ‘*strandingsnetwerk*’. From 2003 to 2007, necropsies were performed on the Dutch island Texel by an international team of marine mammal pathologists and from 2008 to date at the Faculty of Veterinary Medicine of Utrecht University. All necropsies were conducted according to a standard protocol (Kuiken and García Hartmann^1993^).

Basic data for each harbour porpoise included finding date and location, total length, weight, sex and age. Age classes were based on the total body length: neonates/calves < 90 cm, juveniles 90–130 cm, and adults > 130 cm. Examination of reproductive organs was used to confirm the length class differentiation between juveniles and adults. Nutritional condition was assessed through investigation of blubber thickness, musculature and the presence of internal fat, and each case was assigned a nutritive condition code (NCC) on a six-point scale, with NCC1 for porpoises in a very good body condition to NCC6 for emaciated individuals. For research into diet composition, stomachs were collected, visually inspected for abnormalities during necropsy and stored in a sealed plastic bag at − 20°C until further stomach content analyses at Wageningen Marine Research (WMR).

### Stomach content sampling methods

Following a general initial inspection of the stomach, contents were rinsed into a large glass beaker, which was then placed under slowly running water by which heavier prey components (e.g. fish bones, otoliths, eye lenses, squid beaks, worm jaws etc.) remained at the bottom in the container, whereas lighter prey components (tissues, fluids) were gradually flushed out of the sample by overflow of the container (Leopold et al. 2015). Any non-dietary items found during initial visual inspection or detected among the heavier diet items in the bottom of the container, were recorded as ‘foreign bodies’ with descriptive notes, and they were separately labelled and stored. Results of the diet analysis are not used in this paper, except for a quantification of fish prey likely caught when foraging along the bottom [flatfishes and demersal roundfish such as whiting (*Merlangius merlangus)*, sandeels (*Ammodytidae*) and gobies (*Gobiidae*)] as opposed to fish species caught higher up in the water column [pelagic roundfish species, such as the clupeids herring (*Clupea harengus*) and sprat (*Sprattus sprattus*) (Leopold 2015)]. Fish abundance was estimated by the number of otoliths present, or by mass as reconstructed from numbers and sizes of the otoliths.

For the traditional dietary study, except for an initial check of large evident items flowing out at the start of the rinsing, no further attention was given to the material lost by overflowing. As a consequence, lightweight foreign objects, especially when small and/or transparent, may have gone unnoticed. In order to check for such potential losses, and to standardise the size range of foreign bodies quantified, a subset of samples was used that had not yet been processed. In this subset, the overflowing beaker was placed on a metal 1 mm mesh sieve (see Electronic Supplementary Material—S1), and the substances accumulating on the sieve were collected for analysis using a binocular microscope and a dedicated search for further ‘foreign bodies’ within the sample. The 1 mm mesh size matches the standard used in the EU protocols used for monitoring ingested plastics in fulmars (OSPAR 2015) and marine turtles (MSFD-TSGML 2013). Samples processed only by overflow, so without sieving the outflowing water, will be referred to as ‘overflow samples’ whereas the samples processed by the combination of overflow plus sieving are mostly simply addressed as ‘sieved samples’.

Foreign bodies include stones, pieces of shells, wood, plants, plastics and other man-made litter. Sand was also frequently found in stomachs, but not recorded. For this paper, the focus was on plastics or other man-made litter, although to further evaluate proportions of benthic versus pelagic feeding, numerical records of non-food items believed to be present at the bottom were made.

Man-made litter items were attributed to categories and quantified by number and mass following methods used in the monitoring of stomach contents of northern fulmars (Van Franeker et al. 2011; OSPAR 2015). In addition, plastic particles were individually weighed and analysed for their polymeric composition (see photo documentation in the ESM). Polymer characterisation was done with a Phazir handheld near infrared material analyser (NIR; DTS-PHAZIR-1624 for 1600–2400 nm). The Phazir compares spectra to an integrated reference library: results were accepted when the instrument indicated an 80% or higher match between the measured particle and a reference.

Single ‘dust-like’ fibres were ignored because fully clean processing from sampling to microscope work could not be guaranteed. In such conditions, secondary ‘atmospheric’ contamination of samples by dust-like fibres is unavoidable (cf. Foekema et al. 2013; Dekiff et al. 2014; Rummel et al. 2016; Roch and Brinker 2017). Only in one case a bundle of fibres too compact for aerial contamination was included (ESM photo of sample MFL-HAPO-UT0413).

### Data evaluation

Although the results include data on numerical and mass abundance of litter, the main analyses in this paper focus on frequency of occurrence (%FO, incidence or prevalence) among all individuals investigated. Numerical or mass data for litter items are given as population averages with standard error (cf. OSPAR 2015), meaning that averages were calculated over all animals investigated, including the individuals without litter. Differences between groups (by e.g. method, age class or sex) were tested for significance by the 2-sample *z* test to compare sample proportions as described by Sergeant (2017) using the 2-tailed approach and *p* = 0.05 as significance level (http://epitools.ausvet.com.au/content.php?page=z-test-2). For some evaluations, data were grouped into 5-year periods as in the plastic ingestion monitoring in northern fulmars (Van Franeker et al. 2011). The focus was on ingested plastics as these are the easiest to compare to the standard monitoring in fulmars, and because additional non-synthetic litter played a minor role.

The *z* test represents a basic type of single variate group comparisons, without clear knowledge of contributions from, and interactions between, other variables. Unfortunately multivariate approaches using data from individual animals have other limitations in our dataset. First of all, the variable expected to be of high importance, the method (overflow versus sieved) could cause temporal and other bias in the sense that one of the methods was only available for a few recent years. Furthermore, the overflow method in itself is not a fixed variable because it may vary with abundance and characteristics of contents of the stomach, and with personal variations of laboratory staff (speed of waterflow when rinsing water, duration and intensity of visual inspection of the overflowing materials). Nevertheless, using Genstat 18th Edition (Payne et al. 2015) Generalized Linear Mixed Model analyses (GLMM) (Schall 1991) were applied in an attempt to evaluate the role of individual variables of sampling method, year and area of collection, age, sex, condition and individual foraging preferences near bottom or in more pelagic water (by abundance of benthic fish, pelagic fish and non-food items). GLMM analyses were applied to three sets of the sample data: the full dataset, the data restricted to the years 2010–2013 irrespective of the fact that if samples were overflow or sieved, and the data restricted to samples quantified by the standardised method of sieving over a 1 mm sieve. As the model output needs to be viewed with caution, only general results are presented in the article, but full information is provided in the ESM.

## Results

Stomachs of 654 harbour porpoises found dead on the Dutch coast between 2003 and 2013 were analysed (Table 1); two necropsied individuals were from year 2003, 268 from years 2005 to 2009 and 384 from years 2010 to 2014. Overall 7% of the animals were neonates/calves, 72% were ‘juveniles’, and 21% were adults. Nutritional condition varied within all age classes, reflecting a mix of cases that suffered fairly instant mortality (those in normal to good nutritional condition) to animals that had been starving for a longer period of time. Roughly 80% of the stomachs contained food remains. Food remains were scarcely found in neonates/calves, showing many had not yet started independent feeding.

**Table 1:** Sample composition by age class and frequencies of occurrence (%FO) of main stomach contents for all harbour porpoise stomach samples, irrespective of method of analysis

	*n*	Avg body length cm ± SD		Avg condition score ± SD		%FO litter	%FO plastic	%FO food
Neonate	47	81	± 6	3.6	± 1.4	0.0	0.0	21
Juvenile	469	110	± 10	3.6	± 1.5	7.7	7.0	81
Adult	137	146	± 10	3.3	± 1.4	8.0	8.0	83
Unknown	1		–		–	0	0	0
All	654	116	± 20	3.5	± 1.5	7.2	6.7	78

Considering all 654 stomach contents (combining results for samples with or without analysis of the additionally sieved remains), man-made litter was detected in 47 stomachs (%FO 7.2%). In 44 cases this was plastic litter (%FO 6.7%), in three cases this was non-synthetic waste, and one case contained both plastics and non-synthetic waste. In total, 76 litter items were recorded (71× plastic, 3× paper, 1× non-synthetic rope, 1× fishing hook), in most cases just one item per individual, with a maximum of five items. For population averages of number and mass of plastic litter, see Table 2 with further details provided in the ESM in Tables S2 to S4. No litter was found in neonates, but in 7% of the juveniles and 8% of the adults examined, plastic was found in the stomach. Additional details of all individual animals with ingested litter are provided in the ESM (Table S6 and photo documentation).

**Table 2:** Frequency and abundance of plastic litter in Dutch harbour porpoise stomachs in relation to period of sampling and sampling method. Tests refer to differences in %FO between the overflow and overflow plus sieve methods

**A**								
**2003–2013**	*n*	%FO	Number of particles			Mass of particles (g)		
			*n*	± se	(Max)	*g*	± se	(Max)
All	654	7%	0.11	± 0.02	(5)	0.009	± 0.004	(2.6)
Overflow only	572	6%	0.09	± 0.02	(5)	0.010	± 0.005	(2.6)
Overflow + sieve	82	15%	0.23	± 0.07	(4)	0.004	± 0.002	(0.1)
		**p = 0.0031						

**B**								
**2005–2009**	*n*	%FO	Number of particles			Mass of particles (g)		
			*n*	± se	(Max)	*g*	± se	(Max)
All	268	8%	0.15	± 0.04	(5)	0.020	± 0.010	(2.6)
Overflow only	267	8%	0.15	± 0.04	(5)	0.020	± 0.010	(2.6)
Overflow + sieve	1	0%						

**C**								
**2010–2013**	*n*	%FO	Number of particles			Mass of particles (g)		
			*n*	± se	(Max)	*g*	± se	(Max)
All	384	5%	0.08	± 0.02	(4)	0.002	± 0.001	(0.2)
Overflow only	303	3%	0.03	± 0.01	(2)	0.001	± 0.001	(0.2)
Overflow + sieve	81	15%	0.23	± 0.07	(4)	0.004	± 0.002	(0.1)
		***p < 0.0001						

### Consequences of additional sieving of overflowing water

The basic added element of the standardised approach is that all rinsing water is sieved over a 1 mm mesh sieve and that sieved remains are studied under a binocular microscope. Any finds in the sieved remains are added to those detected during autopsy or in the residue inside the overflow glass beaker. Thus, no materials (litter or other) larger than 1 mm can be missed. In the earlier overflow method small or transparent floating items could have been lost with the rinsing water. When comparing the two methods (Table 2), there is a significant difference in the frequency of plastics detected. Measured over all data from all years, samples studied by only overflow indicated that 6% of the stomachs contained plastic, whereas samples investigated with the additional analysis of remains from the sieve showed 15% of stomachs to contain plastic (Table 2A; *z* test *p* = 0.0031). Moreover, it was noted that all but one of the 82 sieved samples originated from the 5-year period starting 2010. During that period (data 2010–2013), the overflow method resulted in a %FO of plastic litter of 3%, whereas the method with the additional sieving indicated a frequency of occurrence of 15% (Table 2C; *z* test *p* < 0.0001). Plastic litter was detected in 7 out of 81 remains in the sieves, in 6 of those no litter had been detected during the earlier dissection and overflow procedures. As would be expected, sizes of litter particles detected in the sieved remains average considerably smaller than those detected at dissection or during the overflow procedure (Table 3). However, without sieving, objects up to at least 5 mm in diameter may be missed, as demonstrated by two industrial granules not detected during the overflow procedure, but only detected later in the remains in the sieve (Tables S4, S6 and photographs). Multivariate GLMM models using individual data confirm a significant difference in detection of plastic litter between the two methods, most strongly so when the analysis was restricted to the 2010–2013 period when both methods had been applied (*p* < 0.001). See the ESM for further details.

**Table 3:** Comparison of litter items detected during overflow procedure with items found in additionally sieved remains after the overflow procedure. Note that these averages refer to number or mass per stomach that did contain litter; for population type averages see Table 2 and supplementary tables

	*n* samples	Number of plastic particles			(Max)	Mass of plastic particles			(Max)
		Total	Avg	± se		Total	Avg	± se	
Overflow	34	52	1.5	± 0.2	(5)	5.6	0.17	± 0.08	(2.6)
Sieved	13	19	1.5	± 0.3	(4)	0.3	0.03	± 0.01	(0.1)
All	47	71	1.5	± 0.2	(5)	6.0	0.13	± 0.06	(2.6)

### Temporal aspect

Due to the uneven distribution of sampling methods over time, the difference between time periods can only be tested for the overflow samples (without sieving the overflowing water): the decrease in %FO of 8% in the 2005–2009 period to 3% in the 2010 and after samples (Table 2A and B) is significant (*z* test *p* = 0.008). Further analysis of temporal data is complicated because analysis of annual data as provided in Table S2, suffers from small sizes of subsamples, low frequencies of occurrence, and erratic inter-annual variations. GLMM models, detailed in the ESM, appear to support a significant role of year of collection when analysing the full dataset, but must be viewed with caution. Year of collection did not play a detectable role over the 2010–2013 period when samples from both methods were available.

### Age and sex differences

As indicated in Table 1, no litter but also little food was found in neonates/calves, which differed strongly from the results for the older age classes. Further evaluation of the differences in ingested plastics between juveniles and adults showed no significant differences when tested for all samples, the overflow samples or the sieved samples. Source data for these comparisons can be found in the detailed tables in the online supplement. When comparing sexes, a significant difference (*z* test *p* = 0.019) within the 82 sieved samples (ESM Table S4), suggests higher incidence of ingestion by male harbour porpoises. However no evident sex differences in plastic ingestion were seen in the larger samples of all animals combined or the overflow samples separately. GLMM models detailed in ESM indicate that sex and age have no correlation to plastic ingestion, irrespective of using the full dataset or restricted ones.

### Foraging aspects

**Fig. 1:**
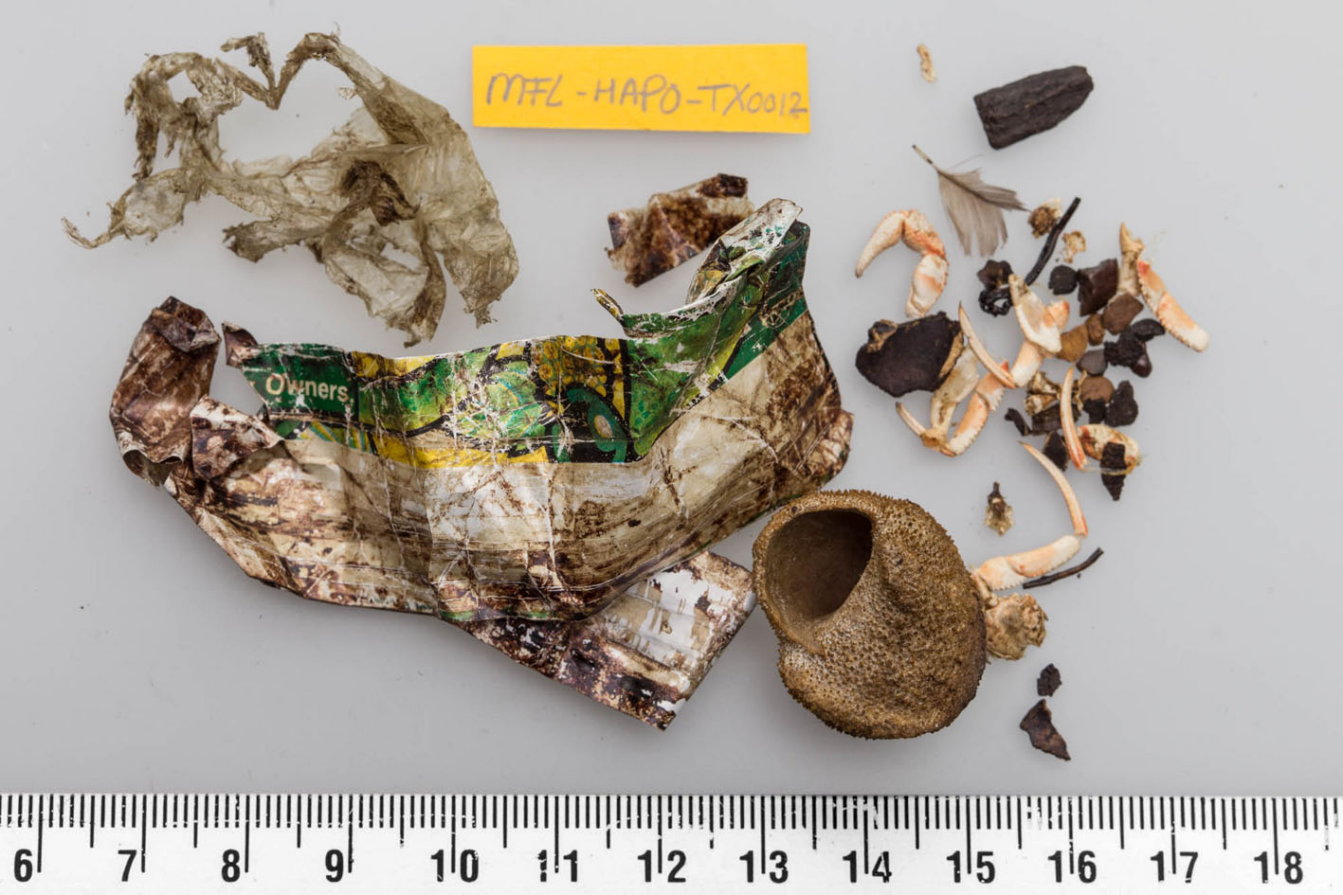
Foreign bodies found in the stomach of harbour porpoise nr. MFL-HAPO-TX0012 with on the left various plastic sheets, and on the right an old shell, hermit crab bits, and a mix of small stones, feather and bits of wood. The ruler scale bar shows size in centimetres

Table 4 shows whether foraging near the bottom as opposed to pelagic foraging higher in the water column might be linked to the ingestion of litter. Prey fish frequency and abundance and non-food frequency and abundance are compared between samples with, or without litter. This was done for the separate sampling methods, and their results combined. In all three comparisons, the frequency of occurrence of benthic fishes and the average number of such fish per stomach are higher in animals that had ingested litter, but only for all samples combined the difference in frequency of benthic fish occurrence was significant (*z* test *p* = 0.044). However, the same was found for the pelagic fish species (*z* test *p* = 0.044). A clearer picture emerges from the analyses of non-food items: both in overflow samples and in the total sample data, the frequency and abundance of non-food items (shells, stones, bog-wood from old peat layers, plant remains etc.) was much higher in animals that had ingested litter (*z* test *p* < 0.0001 in both cases). A similar result is suggested for the sieved samples, but lacks significance possibly due to the reduced sample size. Figure 1 illustrates the presence of non-food materials in a porpoise stomach also containing plastic. In the ESM similar photographs have been added for some of the more extreme cases, to further illustrate the character of such non-food items which almost all are believed to originate from the bottom. GLMM analyses on all samples (see ESM for details) suggest a significant model contribution from correlation between the presence of plastic litter and the mass of ingested bottom fish species. Such correlation was not observed for the mass of pelagic fish species. In GLMM analyses of the full dataset, the numerical abundance of bottom related non-food items, although fairly highly ranked among variables considered, showed no significant contribution to the models. In restricted datasets, the ingested fish mass or abundance of non-food items showed no contribution to the model of plastic ingestion.

**Table 4:** Evaluation of frequency (%FO) and abundance (average number in brackets) of ingested litter in relation to benthic feeding

	*n*	Litter		Bottom fish		Pelagic fish		Non-food items	
		%FO	(*n*)	%FO	(*n*)	%FO	(*n*)	%FO	(*n*)
Overflow samples									
With litter	34	100	(1.6)	82	(143)	35	(10)	32	(5)
Without	538	0	(0.0)	68	(103)	27	(5)	9	(1)
				ns		ns		*p* < 0.0001	
Sieved samples									
With litter	13	100	(1.6)	85	(355)	62	(11)	15	(1)
Without	69	0	(0.0)	81	(197)	42	(8)	6	(0)
				ns		ns		ns	
All samples									
With litter	47	100	(1.6)	83	(202)	43	(10)	28	(4)
Without	607	0	(0.0)	69	(114)	29	(6)	8	(1)
				*p* = 0.044		*p* = 0.044		*p* < 0.0001

### Litter sizes and materials

An indication of average size and mass may be derived from the average particle number and mass data provided in Table 3. Individual details of litter items can be seen in photos in the ESM. Polymer characterisation by NIR was performed on 66 particles. However, 31 of these (47%) showed a less than 80% match to substances in the reference library which is insufficient for a reliable identification. Especially dark particles are problematic as the NIR is based on reflected light. Surface degradation and biofouling as a consequence of long-term exposure in the marine environment may also reduce reliability of polymer assessments because those processes will alter the spectra. Of 35 particles with reliable matches to the reference library, 16 were polyethylene (PE, 46%), 14 polypropylene (PP, 40%), 2 polyamide (PA, 6%), 2 polyvinylchloride (PVC, 6%) and 1 polyethylene terephthalate (PET, 3%).

## Discussion

Over the full study period 2003–2013, and combining different methods, 7% of 654 beached harbour porpoises from the Netherlands had plastic in the stomach. However, using a dedicated protocol with microscopic inspection of all remains from the rinsing beaker and from the 1 mm sieve showed that 15% of 82 harbour porpoises (81 from the 2010–2013 period) had ingested plastic. Group comparisons using the *z* test as well as GLMM analyses of individual data confirmed a highly significant difference in results between the two methods. The dedicated protocol complies with the ones established in the MSFD for the formal monitoring of plastic ingestion by fulmars (MSFD-TSGML 2013; OSPAR 2015) and marine turtles (Matiddi et al. 2017). We strongly recommend this standard protocol to be applied to marine mammals in diet studies where quantification of marine litter is included.

Evaluation of further variables affecting the observed ingestion of marine plastic litter in harbour porpoises is complicated because of the uncertainty on consistency within the overflow method, and the limited time frame and low number of samples analysed by the standard method.

For monitoring purposes, a variable of major importance is that of time. Harbour porpoises studied by the overflow method showed a change in %FO for plastic ingestion from 8% up to the year 2009 to 3% in the years after. Group comparisons by *z* test and GLMM analyses both suggest a significant reduction in plastic incidence, but bias in the data cannot be excluded completely and unfortunately we lack sufficient years of data for standard samples to run a proper analysis. Year was not a significant variable in any analysis on the restricted datasets.

No clear indications exist that nutritive condition, sex or age of harbour porpoises affect plastic ingestion. Among harbour seals, which co-occur with harbour porpoises in the North Sea, younger animals showed higher frequencies of litter in the stomach: plastics were found in 8 of 43 seals up to 3 years of age and in 2 of 48 older seals (*z* test *p* = 0.028) (Bravo Rebolledo et al. 2013). It was suggested that the seals accidentally ingested litter particles when foraging on bottom dwelling or even burrowed prey (Bowen et al. 2002), thus suggesting more frequent bottom feeding among inexperienced youngsters. No age effect was seen in harbour porpoises, but some data do support the idea that plastic ingestion may be related to feeding close to the bottom. Group comparisons indicate a significant positive correlation between the frequency of man-made litter and the frequency of natural benthic non-food items in the stomach. Similarly, some of the GLMM analyses indicate that plastic ingestion is correlated to the mass of benthic fishes consumed. These observations support the idea of accidental intake of man-made litter when capturing benthic preys using suction feeding (MacLeod et al. 2006; Marx et al. 2016).

Identification of the polymer types of plastics ingested by the harbour porpoises was difficult, with almost half of items showing insufficient match with the reference library for a reliable assessment. Among the items that could be reliably identified, at least 86% were plastics with specific weights lower than that of seawater (46% PE; 40%PP). Different types of additives, but certainly biofouling and turbulence may force many of such particles to sink to the bottom (Frère et al. 2017), where most litter items were probably picked up by the harbour porpoises, in combination with other bottom materials.

Quantities of plastics ingested by harbour porpoises appear relatively low, with maxima of 5 items and 2.6 g of plastic in individual animals among the 654 studied. These quantities are unlikely to have caused detectable physical harm, but sub-lethal negative impacts, like partial blockage of passage of food cannot be excluded. Other studies concluded that harbour porpoises occasionally do ingest lethal or seriously harmful items or quantities of litter (Kastelein and Lavaleije 1992; Baird and Hooker 2000), but this was not found in our study.

In published earlier accounts, relatively little comparative information is available on plastic or litter ingestion by harbour porpoises. Some conspicuous cases in individual animals have been published and concerned mainly plastic bags, but also cloth and remains of bananas (Bosch 1950; Walker and Coe 1990; Kastelein and Lavaleije 1992; Baird and Hooker 2000; Bogomolni et al. 2010). Some studies do report plastic ingestion by harbour porpoises among larger samples (Deaville et al. 2010; Haelters et al. 2012; Unger et al. 2017; Lusher et al. 2017), but methods differ and are not always clearly documented and are in most cases less rigorously aimed at detecting plastics than the standardised sieving method. In related porpoise species, ingestion is known from finless porpoise (*Neophocaena phocaenoides*; Baird and Hooker 2000), Burmeister’s porpoise (*Phocoena spinipinnis;* Baulch and Perry 2014), and Dall’s porpoise (*Phocoenoides dalli;* Walker and Coe 1990). However, Walker and Coe (1990) also report 918 Dall’s porpoises in which no ingested plastics were encountered. For further details see the ESM Table S5.

The main result from this study is that without dedicated standard protocols it is not possible to conduct proper comparative studies on litter ingestion by marine mammals, let alone monitoring programmes (cf. Provencher et al. 2017). For example, environmental pollution levels in the German marine areas are likely to be similar to those in the Netherlands. Yet Unger et al. (2017) reported only 0.7% of necropsied harbour porpoises in the North Sea and Baltic to have ingested litter (4 out of 548 individuals, of which two with plastic and one with a fishing hook in the stomach and one animal with a plastic bracelet found in the mouth). Limited to the North Sea the litter ingestion rate was 1.2% (3 of 241). In the German study the gastro-intestinal system was investigated ‘macroscopically’ during the necropsy for the occurrence of marine litter such as plastics, without further dietary work presented. In the UK, Deaville et al. (2010) inspected stomachs macroscopically and reported a %FO of ingested plastic of 2.2% among 495 harbour porpoises. Recent information from Ireland reported that 4.8% of 125 autopsied animals contained litter (Lusher et al. 2017). The lack of detail of non-specific macroscopic inspections hampers a comparison of results. In the Dutch necropsies at the initial opening of the stomach, no notes were made on its contents because of the subsequent more detailed diet study: however, looking at the larger items (see photo’s in online supplement) about 5 of the animals, that is less than 1% of the sample, might have been detected to contain plastic litter at the macroscopic level. This is roughly similar to the results of the German, UK and Irish studies (Deaville et al. 2010; Unger et al. 2017; Lusher et al. 2017) and *z* tests are unable to detect any significant difference between any of these studies. Zero ingested plastics were reported from detailed diet studies using sieved remains of stomach contents in seven harbour porpoises from Normandy (De Pierrepont et al. 2005) and 64 specimens from the Belgian coast (Haelters et al. 2012). The difference with Dutch data likely simply reflects inadequate samples sizes at low frequency of occurrence: for example the 2-sample *z* test does not rate the difference between the Belgian (64 stomachs 0% plastic) and UK samples (495 stomachs 2.2% plastic) as being different, and would require over 170 stomachs analysed in the Belgian sample to test the difference as being significant. Similarly, two datasets from the Black Sea, with one showing zero plastics among 12 necropsied animals (Birkun and Krivokhizhin 2008) and the other 14% of ingested plastic among 42 sieved stomach contents (Tonay et al. 2007) cannot be compared because of their methodological differences. But even assuming comparable methods, the number of samples was too small to label the difference as significant.

In the absence of standardised studies from other regions, harbour porpoise data currently cannot confirm a geographical pattern with a relatively high pollution level in the southern North Sea, as was demonstrated earlier by fulmars (Van Franeker et al. 2011) and to some extent by fishes (Foekema et al. 2013) and bottom trawl data (OSPAR 2017).

Temporal trends and spatial patterns in marine litter occurrence and impacts can be assessed only by the use of standard approaches with dedicated protocols and adequately sized samples.

## Conclusion

Using a standard approach to quantify ingested (plastic) litter in stomachs of harbour porpoises in the Netherlands, a 15% frequency of occurrence of mostly small plastic litter items was observed. In a polluted area such as the southern North Sea, such frequency indicates that ingestion of litter currently represents no major hazard for this species. The low frequency of ingested litter makes the harbour porpoise a less practical species for targeted annual monitoring of change under the EU MSFD as this would require large yearly sample sizes to be processed. However, harbour porpoises, like seals, can provide a measure for plastic abundance in a poorly studied compartment of the water column, that is the water–sediment interface. Future work, in combination with ongoing diet studies, could thus indicate regional differences and long-term temporal changes in benthic litter abundance. The dataset in this paper has clearly demonstrated that such is only possible if data are collected from adequately sized samples using a protocol standardised to established MSFD monitoring efforts. The basic element of the standardisation is to not rely on visual detection of litter during autopsies or rinsing procedures, but to sieve all stomach contents over a 1 mm mesh and inspect all remains under binocular microscope.

## Electronic supplementary material

Below is the link to the electronic supplementary material.

Supplementary material 1 (PDF 4261 kb)
