# Supplementary material for: Plastic ingestion by harbour porpoises Phocoena phocoena in the Netherlands: Establishing a standardised method
Source: Ambio. 2018 Jan 5;47(4):387–97. doi: 10.1007/s13280-017-1002-y (PMC5884759; doi:10.1007/s13280-017-1002-y)
Supplement: Supplementary file 1 — Supplementary material 1 (PDF 4261 kb) [file 13280_2017_1002_MOESM1_ESM.pdf]

Ambio

Electronic Supplementary Material (ESM)

This supplementary material has not been peer reviewed

Title:

Plastic ingestion by harbour porpoises *Phocoena phocoena* in the Netherlands: establishing a standardized method

Authors:
Jan A. van Franeker [1], Elisa L. Bravo Rebolledo[1,2] , Eileen Hesse[1], Lonneke L. IJsseldijk[3], Susanne Kühn[1], Mardik Leopold[1] and Lara Mielke[1]

Corresponding author: Jan A. van Franeker Jan.vanfraneker@wur.nl

1 Wageningen Marine Research (formerly IMARES),
Ankerpark 27, 1781 AG Den Helder, The Netherlands.
2 Elisa Bravo Ecological and Biological Research
3 Department of Pathology, Faculty of Veterinary Medicine, Utrecht University

ESM (*Electronic Supplementary Material)* - Contents

Standardized stomach processing by overflow plus sieving 3

Polymer characterization 4

Table S1 Reference list of polymer spectra available in the handheld Phazir 4

Detailed tables of plastic abundance 5

Table S2 Plastic abundance in stomachs of harbour porpoises from the Netherlands, all sampling methods combined. 6

Table S3 Plastic abundance in stomachs of harbour porpoises from the Netherlands, where stomach contents were only investigated by overflow method for diet study. 7

Table S4 Details for plastic abundance in stomachs of harbour porpoises from the Netherlands, where stomach contents were first investigated by overflow method for diet study plus additional analysis of sample remains captured from the supernatant on a 1 mm sieve. 8

Generalized Linear Mixed Model analyses (GLMM) 9

Literature overview 11

Table S5 Literature overview 11

Stomach content detail in table and photo pages 13

Table S6 Details for harbour porpoises with plastics or other litter in stomachs. 14

Photographs of litter in harbour porpoises.. 16

Standardized stomach processing by overflow plus sieving

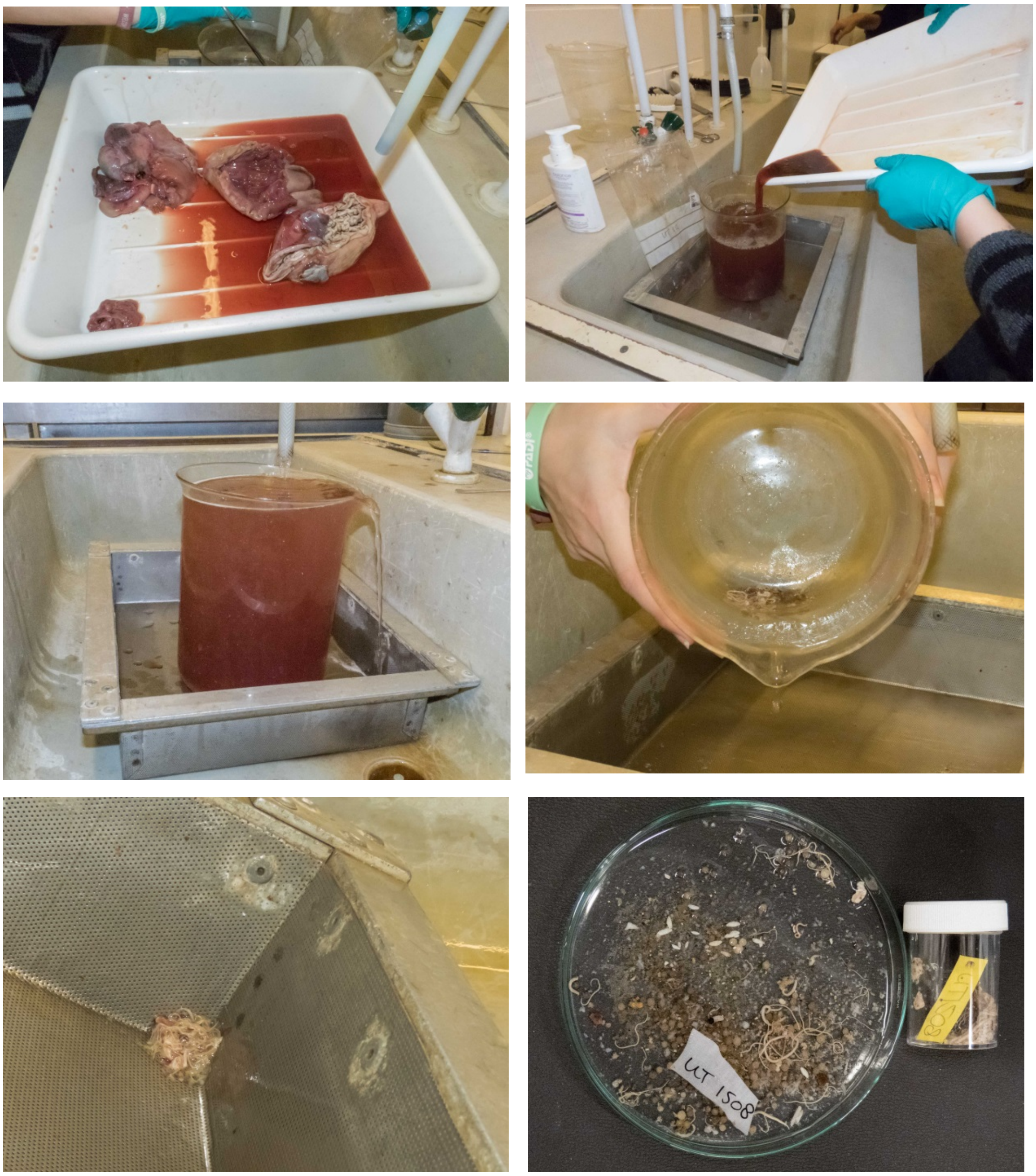

Photographic summary of the overflow method as used in diet studies, but in combination with 1 mm sieving of anything rinsed out of the container. Depending on aims of the study, contents of different stomach compartments (top left) can be treated separately or combined (as here). Stomachs are rinsed out and contents transferred to a glass beaker. The beaker is placed under running water, but on top of a 1 mm sieve tray. Bottom contents of the container are the main sample used for diet study; remaining materials in the sieve are checked for presence of any further material, with special attention for foreign bodies such as plastics. Both samples are checked under binocular microscope. In this standardized approach all stomach contents larger than 1 mm are inspected. General visual inspection of the stomachs, or just overflow without the sieve lack the standardization essential to comparative studies or monitoring.

Polymer characterization

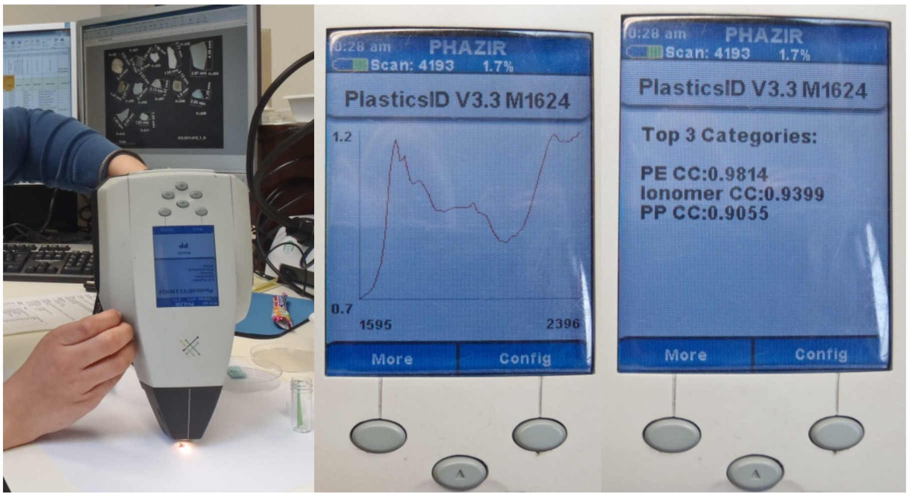

Characterization of polymers was done with a Phazir Handheld Near Infrared Material analyser (NIR; DTS-PHAZIR-1624 for 1600-2400 nm) which was kindly lend to us by Arend Bolt of the Van Gansewinkel group. The Phazir compares the spectra of light reflected by particles to spectra stored an integrated reference library and returns the 3 most likely options, with a percentage for the match between the spectra; we accepted its first choice when the instrument indicated a match between the measured particle and a reference spectrum of 80% or more. Lower matches are treated as 'not identified (NoId). With photographs, when items could not be identified with certainty, we list them as NoId but additionally provide the best first match with percentage of that assessment.

Table S1 Reference list of polymer spectra available in the handheld Phazir

Acronym	Name/description
ABS	Acrylonitrile butadiene styrene
CA	Cellulose acetate
EVA	Ethylene-vinyl acetate
PA	Nylon (polyamide)
PB	Polybutylene
PBT	Polybutylene terephthalate
PC	Polycarbonate
PE	Polyethylene
PET	Polyethylene terephthalate
PETG	Polyethylene terephthalate glycol
PI	Polyimide
PMMA	Polymethyl metacrylate
PMP	Polymethyl pentane
POM	Acetal (Polyoxymethylene)
PP	Polypropylene
PPO	Polyphenylene oxide
PPS	Polyphenolyne sulfide
PS	Polystyrene
PSO	Polysulfone
PTT	Polytrimethylene terephthalate
PUR	Polyurethane
PVC	Polyvinyl chloride
SAN	Styrene acrylonitrile
TPV	Thermoplastic elastomer
Elastomer	Elastomer (in rubbery materials)
Ionomer	Ionomer (in strong packaging films, adhesive layers)
Nylon/ABSblend	Nylon/ABSblend
Styrenic terpolymer	Styrenic terpolymer

Detailed tables of plastic abundance

Details of plastic abundance by sampling method

- all samples combined (Table S2),
- overflow samples (Table S3), and
- sieved samples *(=combination of litter visually detected in stomach + remains in overflow beaker + remains on 1 mm sieve)* (Table S4)

in harbour porpoises in the Netherlands 2003-2013, with specifications for sexes, ages and time periods.

Table S2 **Plastic abundance in stomachs of harbour porpoises from the Netherlands, all sampling methods combined. %FO is for percentage frequency of occurrence of plastic (also known as incidence or prevalence). Numerical and mass abundance of plastic given as averages for all samples, including those without plastic ('population average').**

ALL SAMPLES IRRESPECTIVE OF METHOD			number of particles		mass of particles (g)	
	N	%FO	n ± se	(max)	g ± se	(max)
all	*654*	7%	0.11 ± 0.02	(5)	0.009 ± 0.004	(2.6)
by sex						
females	*280*	7%	0.12 ± 0.03	(5)	0.006 ± 0.003	(0.7)
males	*364*	7%	0.10 ± 0.02	(4)	0.012 ± 0.007	(2.6)
unknown sex	*10*	0%	0.00 ± 0.00	(0)	0.000 ± 0.000	(0.0)
by age						
neonate	*47*	0%	0.00 ± 0.00	(0)	0.000 ± 0.000	(0.0)
juvenile	*469*	7%	0.10 ± 0.02	(4)	0.010 ± 0.006	(2.6)
adult	*137*	8%	0.16 ± 0.06	(5)	0.010 ± 0.005	(0.6)
unknown age	*1*	0%	0.00 ± 0.00	(0)	0.000 ± 0.000	(0.0)
by sex and age						
female neonate	*19*	0%	0.00 ± 0.00	(0)	0.000 ± 0.000	(0.0)
female juvenile	*179*	7%	0.11 ± 0.04	(4)	0.008 ± 0.004	(0.7)
female adult	*82*	7%	0.17 ± 0.08	(5)	0.004 ± 0.004	(0.3)
male neonate	*28*	0%	0.00 ± 0.00	(0)	0.000 ± 0.000	(0.0)
male juvenile	*280*	7%	0.10 ± 0.03	(4)	0.012 ± 0.009	(2.6)
male adult	*55*	9%	0.15 ± 0.08	(4)	0.018 ± 0.012	(0.6)
unknown age	*1*	*0%*	*0.00 ± 0.00*	*(0)*	*0.000 ± 0.000*	*(0.0)*
unknown sex	*10*	*0%*	*0.00 ± 0.00*	*(0)*	*0.000 ± 0.000*	*(0.0)*
by year						
2003	*2*	50%	1.00 ± 1.00	(2)	0.001 ± 0.001	(0.0)
2004	*0*					
2005	*3*	67%	1.33 ± 0.88	(3)	0.063 ± 0.054	(0.2)
2006	*64*	6%	0.08 ± 0.04	(2)	0.004 ± 0.003	(0.2)
2007	*55*	0%	0.00 ± 0.00	(0)	0.000 ± 0.000	(0.0)
2008	*89*	10%	0.24 ± 0.09	(5)	0.054 ± 0.031	(2.6)
2009	*57*	12%	0.18 ± 0.08	(4)	0.002 ± 0.001	(0.1)
2010	*48*	10%	0.19 ± 0.10	(4)	0.010 ± 0.006	(0.2)
2011	*246*	4%	0.06 ± 0.02	(2)	0.001 ± 0.000	(0.0)
2012	*68*	1%	0.01 ± 0.01	(1)	0.000 ± 0.000	(0.0)
2013	*22*	18%	0.23 ± 0.11	(2)	0.000 ± 0.000	(0.0)
by pentade						
2000-2004	*2*	50%	1.00 ± 1.00	(2)	0.001 ± 0.001	(0.0)
2005-2009	*268*	8%	0.15 ± 0.04	(5)	0.020 ± 0.010	(2.6)
2010-2014	*384*	5%	0.08 ± 0.02	(4)	0.002 ± 0.001	(0.2)

Table S3 **Plastic abundance in stomachs of harbour porpoises from the Netherlands, where stomach contents were only investigated by overflow method for diet study. Further details see caption for supplement table 1.**

**OVERFLOW samples**			number of particles		mass of particles (g)	
	n	%FO	n ± se	(max)	g ± se	(max)
ALL	*572*	6%	0.09 ± 0.02	(5)	0.010 ± 0.005	(2.6)
by sex						
females	*249*	7%	0.13 ± 0.04	(5)	0.006 ± 0.003	(0.7)
males	*314*	4%	0.06 ± 0.02	(4)	0.013 ± 0.009	(2.6)
unknown sex	*9*	*0%*	*0.00 ± 0.00*	*(0)*	*0.000 ± 0.000*	*(0.0)*
by age						
neonates	*45*	0%	0.00 ± 0.00	(0)	0.000 ± 0.000	(0.0)
juveniles	*404*	5%	0.08 ± 0.02	(4)	0.011 ± 0.007	(2.6)
adults	*123*	8%	0.15 ± 0.06	(5)	0.010 ± 0.006	(0.6)
by sex and age						
female neonates	*19*	0%	0.00 ± 0.00	(0)	0.000 ± 0.000	(0.0)
female juveniles	*156*	8%	0.12 ± 0.04	(4)	0.008 ± 0.005	(0.7)
female adults	*74*	8%	0.19 ± 0.09	(5)	0.005 ± 0.004	(0.3)
male neonates	*26*	0%	0.00 ± 0.00	(0)	0.000 ± 0.000	(0.0)
male juveniles	*239*	4%	0.06 ± 0.02	(4)	0.013 ± 0.011	(2.6)
male adults	*49*	8%	0.08 ± 0.04	(1)	0.018 ± 0.013	(0.6)
unknown sex	*9*	*0%*	*0.00 ± 0.00*	*(0)*	*0.000 ± 0.000*	*(0.0)*
by year						
2003	*2*	50%	1.00 ± 1.00	(2)	0.001 ± 0.001	(0.0)
2004	*0*					
2005	*3*	67%	1.33 ± 0.88	(3)	0.063 ± 0.054	(0.2)
2006	*64*	6%	0.08 ± 0.04	(2)	0.004 ± 0.003	(0.2)
2007	*55*	0%	0.00 ± 0.00	(0)	0.000 ± 0.000	(0.0)
2008	*88*	10%	0.24 ± 0.09	(5)	0.054 ± 0.031	(2.6)
2009	*57*	12%	0.18 ± 0.08	(4)	0.002 ± 0.001	(0.1)
2010	*40*	5%	0.05 ± 0.03	(1)	0.006 ± 0.006	(0.2)
2011	*207*	3%	0.04 ± 0.02	(2)	0.000 ± 0.000	(0.0)
2012	*49*	0%	0.00 ± 0.00	(0)	0.000 ± 0.000	(0.0)
2013	*7*	0%	0.00 ± 0.00	(0)	0.000 ± 0.000	(0.0)
by pentade						
2000-2004	*2*	50%	1.00 ± 1.00	(2)	0.001 ± 0.001	(0.0)
2005-2009	*267*	8%	0.15 ± 0.04	(5)	0.020 ± 0.010	(2.6)
2010-2014	*303*	3%	0.03 ± 0.01	(2)	0.001 ± 0.001	(0.2)

Table S4 **Details for plastic abundance in stomachs of harbour porpoises from the Netherlands, where stomach contents were first investigated by overflow method for diet study plus additional analysis of sample remains captured from the supernatant on a 1 mm sieve. Further details see caption for supplement table 1.**

**OVERFLOW+SIEVED samples**			number of particles		mass of particles (g)	
	N	%FO	n ± se	(max)	g ± se	(max)
ALL	*82*	15%	0.23 ± 0.07	(4)	0.004 ± 0.002	(0.1)
by sex						
females	*31*	3%	0.03 ± 0.03	(1)	0.004 ± 0.004	(0.1)
males	*50*	22%	0.36 ± 0.11	(4)	0.004 ± 0.002	(0.1)
unknown sex	*1*	*0%*	*0.00 ± 0.00*	*(0)*	*0.000 ± 0.000*	*(0.0)*
by age						
neonates	*2*	0%	0.00 ± 0.00	(0)	0.000 ± 0.000	(0.0)
juveniles	*65*	17%	0.23 ± 0.07	(2)	0.003 ± 0.002	(0.1)
adults	*14*	7%	0.29 ± 0.29	(4)	0.008 ± 0.008	(0.1)
unknown age	*1*	*0%*	*0.00 ± 0.00*	*(0)*	*0.000 ± 0.000*	*(0.0)*
by sex and age						
female neonates	*0*					
female juveniles	*23*	4%	0.04 ± 0.04	(1)	0.005 ± 0.005	(0.1)
female adults	*8*	0%	0.00 ± 0.00	(0)	0.000 ± 0.000	(0.0)
male neonates	*2*	0%	0.00 ± 0.00	(0)	0.000 ± 0.000	(0.0)
male juveniles	*41*	24%	0.34 ± 0.10	(2)	0.003 ± 0.001	(0.0)
male adults	*6*	17%	0.67 ± 0.67	(4)	0.018 ± 0.018	(0.1)
unknown age	*1*	*0%*	*0.00 ± 0.00*	*(0)*	*0.000 ± 0.000*	*(0.0)*
unknown sex	*1*	*0%*	*0.00 ± 0.00*	*(0)*	*0.000 ± 0.000*	*(0.0)*
by year						
2003	*0*					
2004	*0*					
2005	*0*					
2006	*0*					
2007	*0*					
2008	*1*	0%	0.00 ± 0.00	(0)	0.000 ± 0.000	(0.0)
2009	*0*					
2010	*8*	38%	0.88 ± 0.52	(4)	0.028 ± 0.018	(0.1)
2011	*39*	10%	0.15 ± 0.08	(2)	0.002 ± 0.001	(0.0)
2012	*19*	5%	0.05 ± 0.05	(1)	0.000 ± 0.000	(0.0)
2013	*15*	27%	0.33 ± 0.16	(2)	0.001 ± 0.000	(0.0)
by pentade						
2000-2004	*0*					
2005-2009	*1*	0%	0.00 ± 0.00	(0)	0.000 ± 0.000	(0.0)
2010-2014	*81*	15%	0.23 ± 0.07	(4)	0.004 ± 0.002	(0.1)

Generalized Linear Mixed Model analyses (GLMM)

GLLM analyses were conducted in order to check for variables potentially correlated to plastic presence in harbour porpoises. Multi-variate model analyses were performed using:

1) the full data-set (n=654),
2) the data restricted to years for which samples of both types of processing were available (n=384)
3) samples processed by the standard method (overflow followed by sieving; n=82)

A major problem with dataset 1 is that it is biased in the sense that the standardized method of sample processing (=overlflow+sieving), has virtually not been applied to samples prior to year 2010. A problem affecting analyses of datasets 1 as well as 2 is that 'the overflow method' is not a fixed situation, as variations in detection of litter may have been caused by variability in abundance and decay level of natural stomach contents, or by different persons using different speeds or duration for the overflow rinsing and/or giving different attention to outflowing materials. These problems are of course avoided if only using the standardized samples for an analysis of further variates affecting records of plastic ingestion. However, that sample is currently still very small.
The analyses were conducted using Genstat 18th edition and are based on REsidual Maximum Likelihood (REML). A range of models using different variable selections in different sequences was analysed, which in general resulted in fairly similar significance level of a variable in the models. Results shown below give the Genstat output for models using all variables in a (in general) decreasing level of importance in the models

GLLM Method c.f. Schall (1991) Biometrika 78: 719-727.
Response variate: PLAincT *(=plastic presence: absent=0 present=1)*
Distribution: binomial Link function: logit
Variables used in models
STC1mm = factor (sample method: 0 for only overflow; 1 for overflow + sieve)
YEAR = variate (years 2003 to 2013, or subset)
DEMFISH_G = variate (mass of benthic fish reconstructed from otoliths)
NNFO = variate (number of non-food items like shells, stones, bogwood, etc)
PELFISH_G = variate (mass of pelagic fish reconstructed from otoliths)
AGE = factor (Adult, Juvenile, Neonate or unknown)
SEX = factor (Male; Female; or unknown)
REGION = factor (one of 8 subareas in the Netherlands or unknown)
NCC = variate (Nutritive Body Code from 1 for very good, to 6 very poor)
After initial trials, body condition (NCC) was left out of model runs because warnings by GENSTAT indicated that many missing values affected model runs, and no indication existed of any correlation to plastic ingestion.

Model 1: All data ***n=654*** *(572 overflow and 82 standard method (overflow + sieved)*

Constant + STC1mm + YEAR + demfish_g + NNFO + pelfish_g + Age + Sex + Region

Sequentially adding terms to fixed model

**Fixed term**	**Wald statistic**	**n.d.f.**	**F statistic**	**d.d.f.**	**F pr**
STC1MM	5.91	1	5.91	635	0.015
YEAR	5.63	1	5.63	635	0.018
DEMFISH_G	6.09	1	6.09	635	0.014
NNFO	1.59	1	1.59	635	0.207
PELFISH_G	1.07	1	1.07	635	0.302
AGE	1.29	3	0.43	635	0.731
SEX	0.12	2	0.06	635	0.941
REGION	1.88	8	0.23	635	0.984

Dropping individual terms from full fixed model

Fixed term	Wald statistic	n.d.f.	F statistic	d.d.f.	F pr
STC1MM	12.70	1	12.70	635	<0.001
YEAR	5.52	1	5.52	635	0.019
DEMFISH_G	2.05	1	2.05	635	0.153
NNFO	3.27	1	3.27	635	0.071
PELFISH_G	1.70	1	1.70	635	0.192
AGE	1.41	3	0.47	635	0.703
SEX	0.10	2	0.05	635	0.951
REGION	1.88	8	0.23	635	0.984

Model 2: data restricted to years when both methods had been applied data 2010-2013 ***n=384*** *(303 overflow and 81 standard method (overflow + sieved)*

Constant + STC1mm + YEAR + demfish_g + NNFO + pelfish_g + Age + Sex + Region

Sequentially adding terms to fixed model

Fixed term	Wald statistic	n.d.f.	F statistic	d.d.f.	F pr
STC1MM	11.59	1	11.59	365	<0.001
YEAR	1.18	1	1.18	365	0.278
DEMFISH_G	1.21	1	1.21	365	0.272
NNFO	0.63	1	0.63	365	0.428
PELFISH_G	0.21	1	0.21	365	0.650
AGE	0.06	3	0.02	365	0.996
SEX	0.15	2	0.07	365	0.930
REGION	4.85	8	0.61	365	0.773

Dropping individual terms from full fixed model

Fixed term	Wald statistic	n.d.f.	F statistic	d.d.f.	F pr
STC1MM	15.76	1	15.76	365	<0.001
YEAR	1.11	1	1.11	365	0.294
DEMFISH_G	0.09	1	0.09	365	0.769
NNFO	0.10	1	0.10	365	0.749
PELFISH_G	0.20	1	0.20	365	0.653
AGE	0.12	3	0.04	365	0.989
SEX	0.18	2	0.09	365	0.914
REGION	4.85	8	0.61	365	0.773

Model 3: data for sieved samples (standardized method)

data n=82 *(standard method = overflow+sieve)*

Constant + YEAR + demfish_g + NNFO + pelfish_g + Age + Sex

Sequentially adding terms to fixed model

Fixed term	Wald statistic	n.d.f.	F statistic	d.d.f.	F pr
YEAR	0.10	1	0.10	72	0.756
DEMFISH_G	0.51	1	0.51	72	0.479
NNFO	0.30	1	0.30	72	0.586
PELFISH_G	0.84	1	0.84	72	0.362
AGE	0.09	3	0.03	72	0.993
SEX	3.28	2	1.64	72	0.201

Dropping individual terms from full fixed model

Fixed term	Wald statistic	n.d.f.	F statistic	d.d.f.	F pr
YEAR	0.01	1	0.01	72	0.920
DEMFISH_G	0.20	1	0.20	72	0.657
NNFO	1.60	1	1.60	72	0.210
PELFISH_G	1.26	1	1.26	72	0.266
AGE	0.16	3	0.05	72	0.983
SEX	3.28	2	1.64	72	0.201

Region omitted from this model because causing fault messages in this model calculation

Literature overview

Table S5 **Literature overview of records of litter ingestion in harbour porpoises and other porpoise species.**

**A. harbour porpoise - individual records**				
source	area	period	sample	notes
Bosch 1950	Netherlands	1950	1	Unripe banana
Walker and Coe 1990	US Coast	1963-1986	1	Piece of cloth and plastic
Kastelein and Lavaleije 1992	North Sea, Netherlands	1991	1	Failed rehabilitation: regurgitated plastic bag, nylon fishing and a banana peel likely blocking passage
Baird and Hooker 2000	Nova Scotia, Canada	1997	1	Ball of plastic blocking passage to stomach
Bogomolni et al. 2010	Massachusetts, USA	2000-2006	?	30 animals studied, but unclear how many stomachs and how investigated; one animal reported with diverse plastic items

**B. harbour porpoise – larger sample studies**					
Source	area	period	sample size	%FO	method plastic detection
De Pierrepont et al. 2005	Normandy, France	1998-2003	7	0	sieved 1mm mesh size
Haelters et al. 2012	North Sea, Belgium	1997-2011	64	0	sieved (315µm)
Deaville et al. 2010	United Kingdom	2005-2010	459	2.2%	visual examination
Unger et al. 2017	North and Baltic Seas, Germany	1990-2014	548	0.7%	visual examination (North Sea only 1.2%FO in 241 animals)
Lusher et al. 2017	Ireland	1990-2015	125	4.8%	Visual examination *(21 samples sieved over 0.118 µm all had microplastics almost all fibres)*
Birkun and Krivokhizhin 2014	Black Sea, Ukraine	2008-2009	12	0	unclear
Tonay et al. 2007	Black Sea, Turkey	2002-2003	42	11.9%	sieved (200µm)

**C. other porpoise species plastic ingestion**						
source	species	area	period	Sample size	%FO	notes
Baird and Hooker 2000	Finless Porpoise (*Neophocaena phocaenoides*)	?	?	?	+	Unpublished(C. Parsons pers comm)
Baulch and Perry 2014	Burmeister's Porpoise (*Phocoena spinipinnis*)	?	?	?	+	Unpublished: 2 known Denuncio pers comm)
Walker and Coe 1990 (Table 1)	Dall's Porpoise (*Phocoenoides dalli*)	California	1973, 1978	?	+	3 individual cases with mainly plastic bags
Walker and Coe 1990 (Table 2)	Dall's Porpoise (*Phocoenoides dalli*)	Japan (86), North Pacific (815),California (17)	1958-1988	918	0	Unclear methods

References

Baird, R.W., and Hooker, S.K. 2000. Ingestion of plastic and unusual prey by a juvenile Harbour Porpoise. Marine Pollution Bulletin 40: 719-720. http://www.sciencedirect.com/science/article/pii/S0025326X00000515)

Baulch, S., and Perry, C. 2014. Evaluating the impacts of marine debris on cetaceans. Marine Pollution Bulletin 80, 210-221. http://dx.doi.org/10.1016/j.marpolbul.2013.12.050

Birkun, A.A.Jr., and Krivokhizhin, S. 2008. Involvement of Black Sea artisanal fisheries in anti-bycatch and anti-marine litter activities. Report to the Permanent Secretariat of the Commission on the Protection of the Black Sea Against Pollution (BSC) and Permanent Secretariat of the Agreement on the Conservation of Cetaceans of the Black Sea, Mediterranean Sea and contiguous Atlantic area (ACCOBAMS). 52 pp. https://accobams.org/file.php/1481/Annex%20D%20Final%20Report.pdf

Bogomolni, A.L., Pugliares, K.R., Sharp, S.M., Patchett, K., Harry, C.T., LaRocque, J.M., Touhey, K.M., and Moore, M.J. 2010. Mortality trends of stranded marine mammals on Cape Cod and southeastern Massachusetts, USA, 2000 to 2006. Diseases of Aquatic Organisms 88: 143-155. https://www.ncbi.nlm.nih.gov/pubmed/20225675

Bosch G. 1950. Bruinvis eet banaan. Vanellus 3(5): 58

De Pierrepont, J.F., Dubois, B., Desormonts, S., Santos, M.B., and Robin, J.P. 2005. Stomach contents of English Channel cetaceans stranded on the coast of Normandy. Journal of the Marine Biological Association of the United Kingdom 85: 1539-1546. https://doi.org/10.1017/S0025315405012762

Deaville, R., Jepson, P.D., Brownlow, A., Reid, R.J., Smith, B., Duffell, E.L., Sabin, R.C., Penrose, R., and Perkins, M. 2010. CSIP Final Report for the period 1 st January 2005 - 31 st December 2010 (Covering contract numbers CR0346 and CR0364). UK Cetacean Strandings Investigation Programme, 98pp www.ukstrandings.org

Haelters, J., Kerckhof, F., Toussaint, E., Jauniaux, T., Degraer, S. 2012. The diet of harbour porpoises bycaught or washed ashore in Belgium, and relationship with relevant data from the strandings database. Royal Belgian Institute of Natural Sciences, 46pp. www.vliz.be/imisdocs/publications/240631.pdf

Kastelein, R.A., and Lavaleije, S.S. 1992. Foreign bodies in the stomach of a female harbour porpoise (*Phocoena phocoena*) from the North Sea. Aquatic Mammals 18: 40-46. http://www.aquaticmammalsjournal.org/share/AquaticMammalsIssueArchives/1992/Aquatic_Mammals_18_2/Kastelein.pdf

Lusher, A.L., Hernandez-Milian, G., Berrow, S., Rogan, E. and O'Connor, I. 2017. Incidence of marine debris in cetaceans stranded and bycaught in Ireland: Recent findings and a review of historical knowledge. Environmental Pollution xx: xx-xx (online) https://doi.org/10.1016/j.envpol.2017.09.070

Payne, R., Murray, D. & Harding, S. 2015. An Introduction to the Genstat Command Language (18th Edition).. VSN International. Hemel Hempstead, UK. 133pp. http://cdn.vsni.co.uk/downloads/genstat/release18/doc/CommandIntro.pdf

Schall, R. 1991. Estimation in generalized linear models with random effects. Biometrika 78: 719-727. https://doi.org/10.1093/biomet/78.4.719

Tonay, A.M., Dede, A., Öztürk, A.A., and Öztürk, B. 2007. Stomach content of harbour porpoises (*Phocoena phocoena*) from the Turkish Western Black Sea in spring and early summer. Rapport Commission Internationale pour L'Exploration Scientifique de la Mer Méditerranée, 38, 616. http://citeseerx.ist.psu.edu/viewdoc/download?doi=10.1.1.540.1963&rep=rep1&type=pdf.

Unger, B., Herr, H., Benke, H., Böhmert, M., Burkhardt-Holm, P., Dähne, M., Hillmann, M., Wolff-Schmidt, K., Wohlsein, P. & Siebert, U. 2017. Marine debris in harbour porpoises and seals from German waters. Marine Environmental Research 130: 77-84. https://doi.org/10.1016/j.marenvres.2017.07.009

Walker, W.A., and Coe, J.M. 1990. Survey of marine debris ingestion by odontocete cetaceans. p 747-774 in: Shomura, R.S. and M.L. Godfrey (Eds). Proceedings of the second international conference on marine debris, 2-7 April 1989, Honolulu, Hawaii. U.S. Dep. Commer., NOAA Techn. Memo. NMFS, NOAA-TM-NMFS-SFWC-154. http://citeseerx.ist.psu.edu/viewdoc/download?doi=10.1.1.573.3472&rep=rep1&type=pdf

Stomach content detail in table and photo pages

The table (S6) shows a listing of all harbour stomach content samples that contained litter, with sample number, year of collection, number of industrial and user plastics or other non-synthetic materials, and whether items were detected in the overflow phase of sample processing, or only after microscopic inspection of materials collected from the 1 mm sieve.

Photographs following the table show all stomachs contents of the harbour porpoises that contained litter listed in Table S6.
In photos particle sizes can be read from scale bars: scale numbers refer to centimetres, with 0.5 cm and 1 mm submarkers. Notes with photo's provide information on plastic category (industrial, user sheet, thread, foam, fragment or other), the polymer basis of the items shown as assessed by Phazir Hand-held NIR (see above) and mass of items (in gram to 4th decimal).
In many cases the polymer basis could not be identified. Such cases texts mention **No ID** (= not identified), but nevertheless the highest polymer matching type with the library is provided in brackets with the percentage. For example this could be **Sheet No ID (PP 73%)** for a sheetlike plastic item possibly being polypropylene, but with an insufficient match to consider the identification reliable.
When the match to the reference library was 80% or higher the information is provided as for example **Fragment PE (86%)** for a certain particle of Polyethylene plastic at 86% match.
Acronyms for plastic types are given in Table S1.

Table S6 **Details for harbour porpoises with plastics or other litter in stomachs. For the two detection columns on the right, 1 means that plastic was detected during the indicated procedure, 0 that is was not detected. Blanks in the last column indicate that no sieved remains were collected and studied.**

Plastics

Sample number	year	number of industrial plastics	number of user plastics	**total number of plastic particles**	**total mass of plastic particles (g)**	detected during flow procedure	detected in sieved remains
MFL-HAPO-TX0003	2005	0	1	**1**	**0.0192**	1	
MFL-HAPO-TX0005	2003	0	2	**2**	**0.0016**	1	
MFL-HAPO-TX0006	2006	0	1	**1**	**0.0123**	1	
MFL-HAPO-TX0011	2006	0	1	**1**	**0.0196**	1	
MFL-HAPO-TX0012	2005	0	3	**3**	**0.1710**	1	
MFL-HAPO-TX0038	2006	0	2	**2**	**0.2080**	1	
MFL-HAPO-TX0049	2006	0	1	**1**	**0.0038**	1	
MFL-HAPO-UT0101	2008	0	4	**4**	**0.1354**	1	
MFL-HAPO-UT0108	2008	0	4	**4**	**0.0158**	1	
MFL-HAPO-UT0122	2008	0	3	**3**	**0.2234**	1	
MFL-HAPO-UT0145	2008	0	1	**1**	**0.3138**	1	
MFL-HAPO-UT0151	2008	0	5	**5**	**0.2960**	1	
MFL-HAPO-UT0153	2008	0	1	**1**	**0.6086**	1	
MFL-HAPO-UT0164	2008	0	1	**1**	**2.5722**	1	
MFL-HAPO-UT0177	2008	0	1	**1**	**0.0037**	1	
MFL-HAPO-UT0180	2008	0	1	**1**	**0.6596**	1	
MFL-HAPO-UT0193	2009	0	1	**1**	**0.0059**	1	
MFL-HAPO-UT0197	2009	0	1	**1**	**0.0023**	1	
MFL-HAPO-UT0201	2009	0	1	**1**	**0.0003**	1	
MFL-HAPO-UT0208	2009	0	1	**1**	**0.0010**	1	
MFL-HAPO-UT0210	2009	0	4	**4**	**0.0153**	1	
MFL-HAPO-UT0232	2009	0	1	**1**	**0.0746**	1	
MFL-HAPO-UT0238 *	2009	0	1	**1**	**0.0100**	1	
MFL-HAPO-UT0348	2010	0	1	**1**	**0.0114**	1	
MFL-HAPO-UT0360	2010	0	4	**4**	**0.1104**	1	0
MFL-HAPO-UT0384	2010	0	1	**1**	**0.1137**	1	0
MFL-HAPO-UT0413**	2010	0	2	**2**	**0.0021**	1	1
MFL-HAPO-UT0435	2011	0	1	**1**	**0.0074**	0	1
MFL-HAPO-UT0444	2011	0	1	**1**	**0.0037**	1	
MFL-HAPO-UT0471	2011	0	1	**1**	**0.0071**	1	
MFL-HAPO-UT0510	2011	0	1	**1**	**0.0041**	1	
MFL-HAPO-UT0511	2011	0	1	**1**	**0.0013**	1	
MFL-HAPO-UT0516	2011	0	2	**2**	**0.0335**	1	

MFL-HAPO-UT0532	2010	0	1	**1**	**0.2457**	1	
MFL-HAPO-UT0605	2011	0	1	**1**	**0.0032**	1	
MFL-HAPO-UT0617	2011	0	1	**1**	**0.0111**	1	
MFL-HAPO-UT0687	2011	0	1	**1**	**0.0389**	1	0
MFL-HAPO-UT0714	2012	0	1	**1**	**0.0001**	0	1
MFL-HAPO-UT0799	2011	1	1	**2**	**0.0344**	0	1
MFL-HAPO-UT0867	2013	0	1	**1**	**0.0001**	1	0
MFL-HAPO-UT0932	2011	1	1	**2**	**0.0155**	0	1
MFL-HAPO-UT0945	2013	0	1	**1**	**0.0006**	0	1
MFL-HAPO-UT0974	2013	0	2	**2**	**0.0050**	1	0
MFL-HAPO-UT0983	2013	0	1	**1**	**0.0031**	0	1
**44**	**totals**	**2**	**69**	**71**	**6.0258**	**38**	**7**

Non synthetic litter

Sample number	year	*non synthetic litter description*	number of other litter	mass of other litter	detected during flow procedure	detected in sieved remains
MFL-HAPO-TX0040	2006	*paper*	1	0.0051	1	
MFL-HAPO-UT0221	2009	*non synthetic rope*	1	1.1426	1	
MFL-HAPO-UT0238 *	2009	*fishhook*	1	0.1000	1	
MFL-HAPO-UT0674	2011	*paper*	2	0.0001	1	0
**4**	**totals**		**5**	**1.2478**	**4**	**0**

Plastic and other litter totals

number of positive samples (in 654 stomachs investigated)	years	number of industrial plastics	number of user plastics	total number of litter	total mass litter	detected during flow procedure	detected in sieved remains
**47 ***	**2003-13**	**2**	**69**	**76**	**7.2736**	**42**	**7**

<u>notes</u>

* MFL-HAPO-UT0238 had both synthetic (nylon line) and non-synthetic litter (metal fishhook) in one item.

** In 6 out 7 cases where plastics were detected in sieved remains, no litter had been detected before,

only in MFL-HAPO-UT0413 plastics were detected during overflow as well as in sieved remains

Photographs of litter in harbour porpoises

MFL-HAPO-TX0003

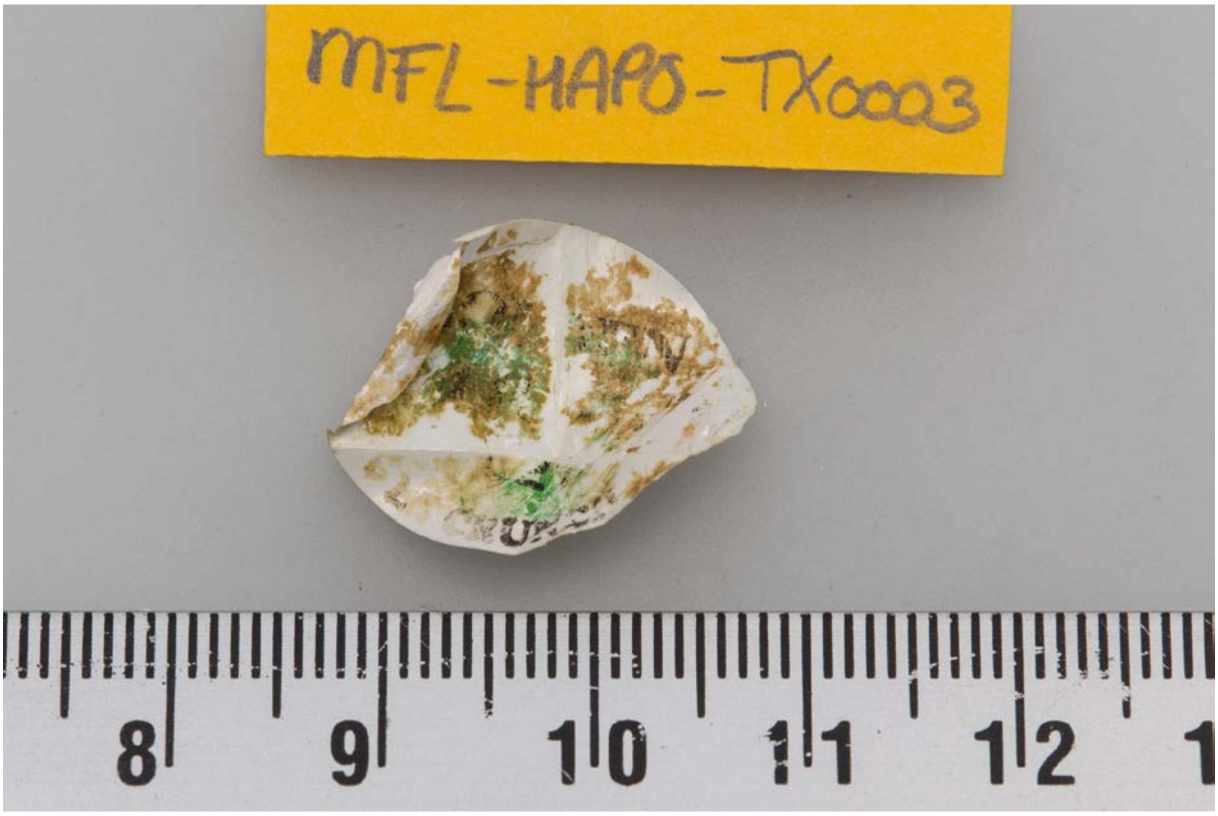

Sheet No ID (PP 53%) Mass 0.0192 g

MFL-HAPO-TX0005

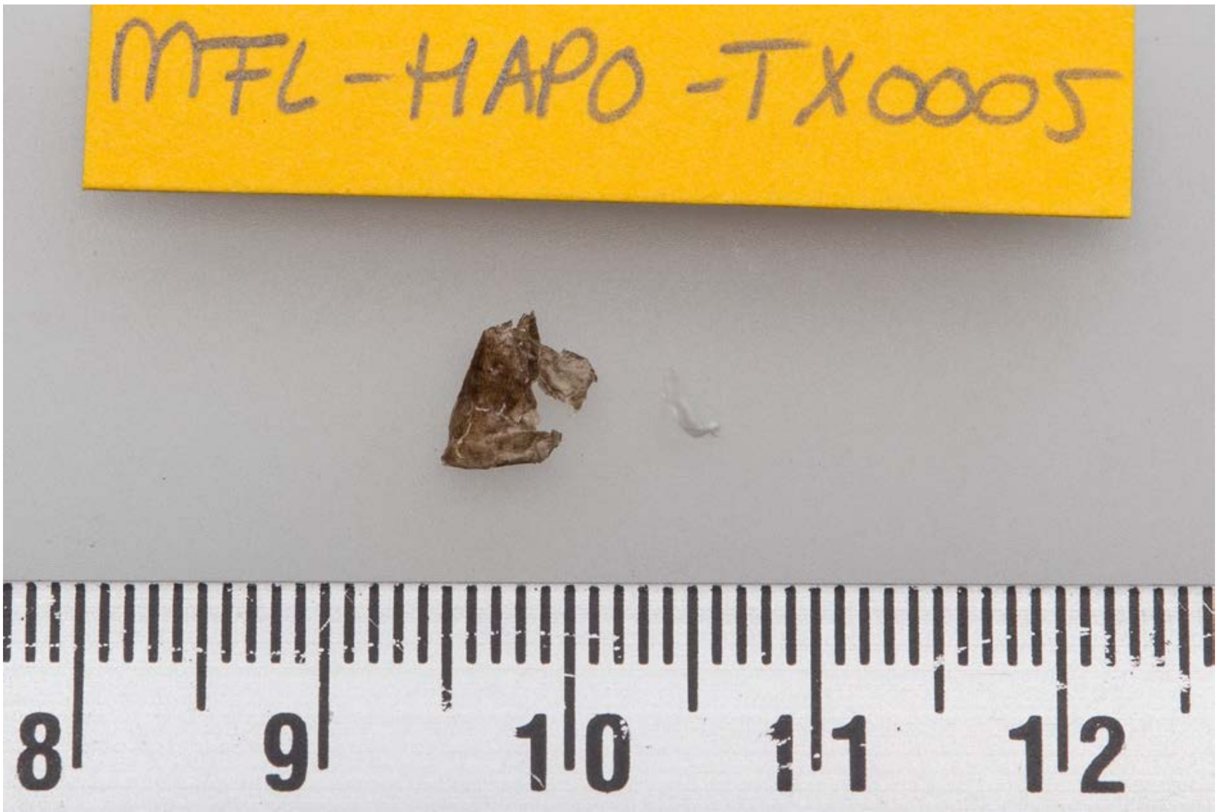

Left Sheet No ID (PA 72%) Mass 0.0016 g;

Right Sheet No NIR assessment or separate mass recorded

MFL-HAPO-TX0006

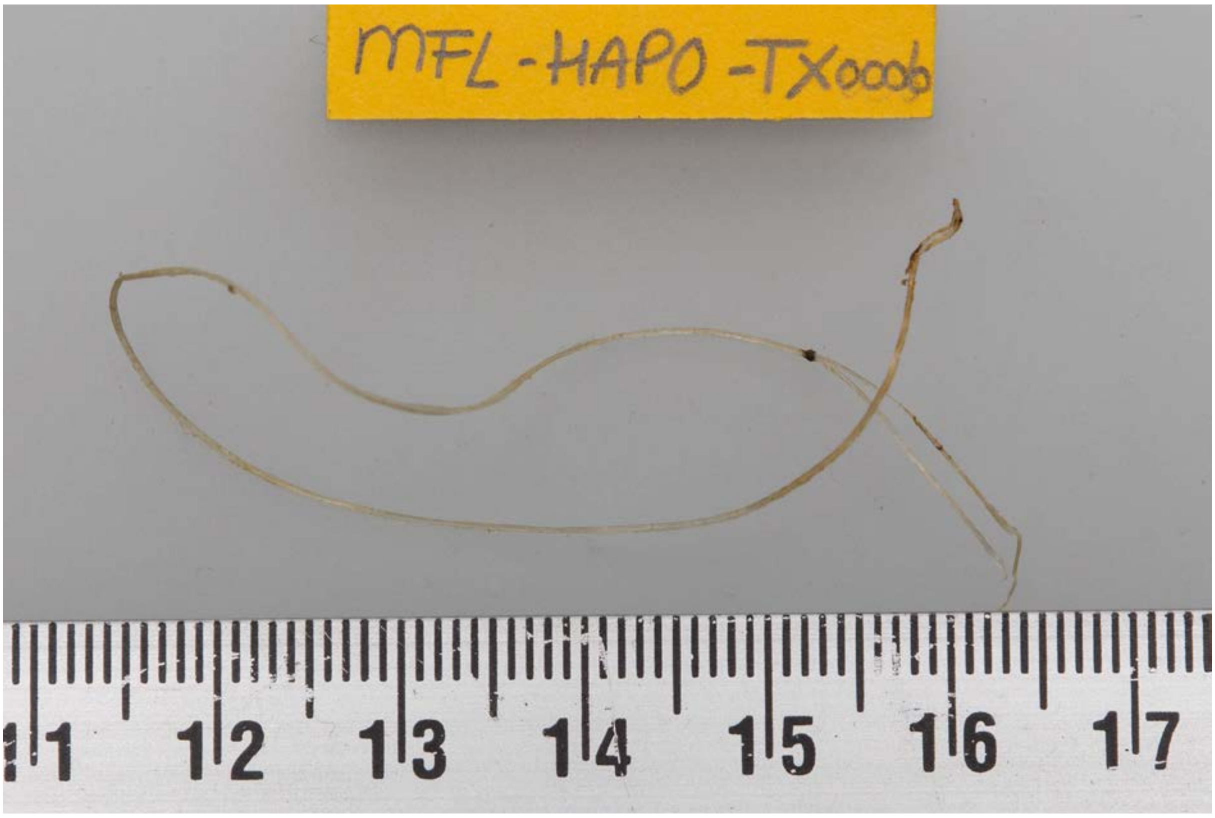

Thread PP (88%) Mass 0.0123 g

MFL-HAPO-TX0011

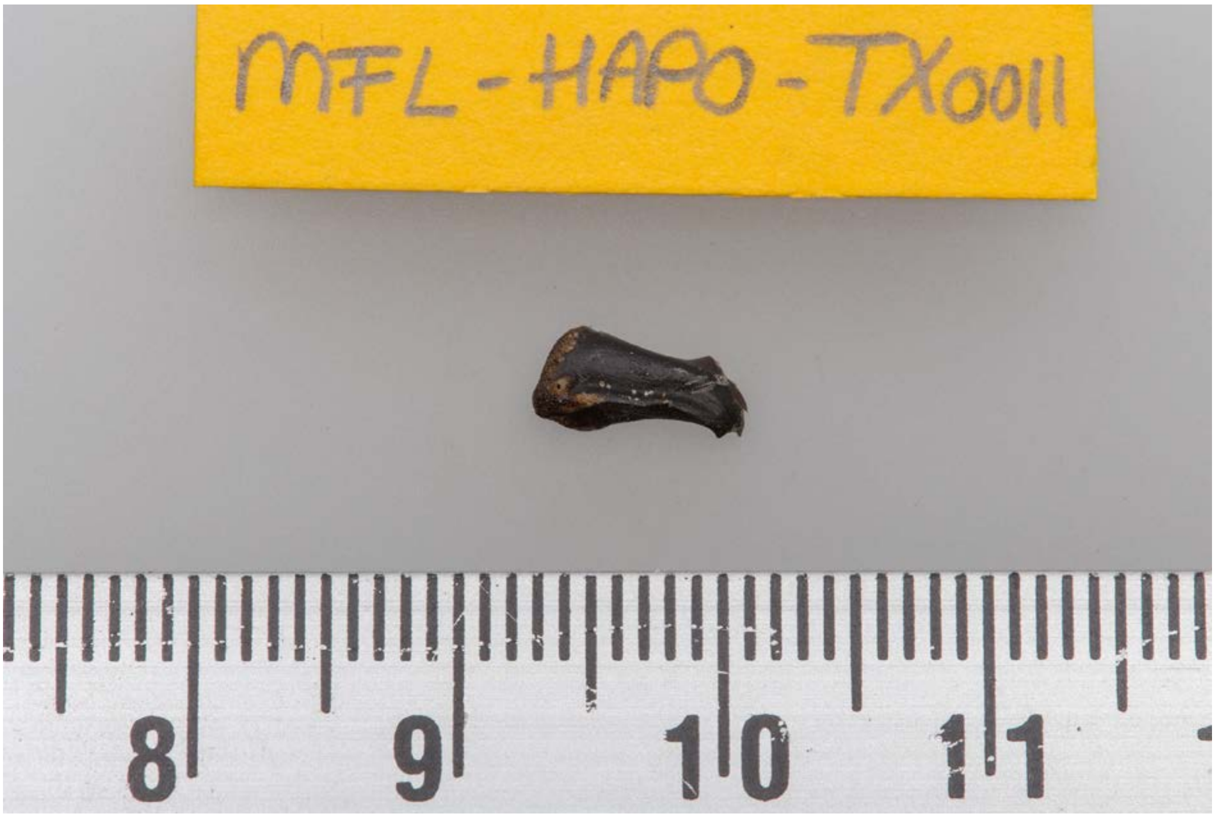

Fragment No ID (PA 72%) Mass 0.0196 g

MFL-HAPO-TX0012

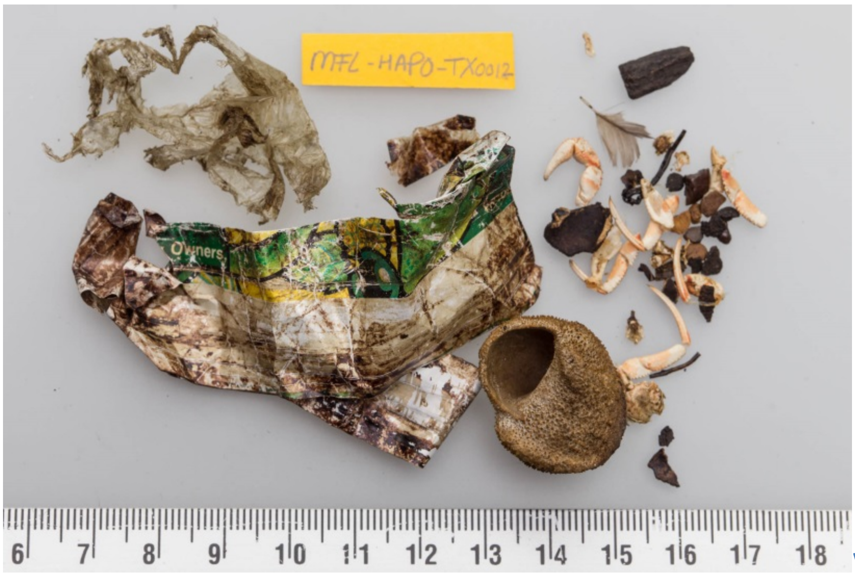

with natural items, a snail shell, hermite crab legs, feather and small pieces of bog-wood

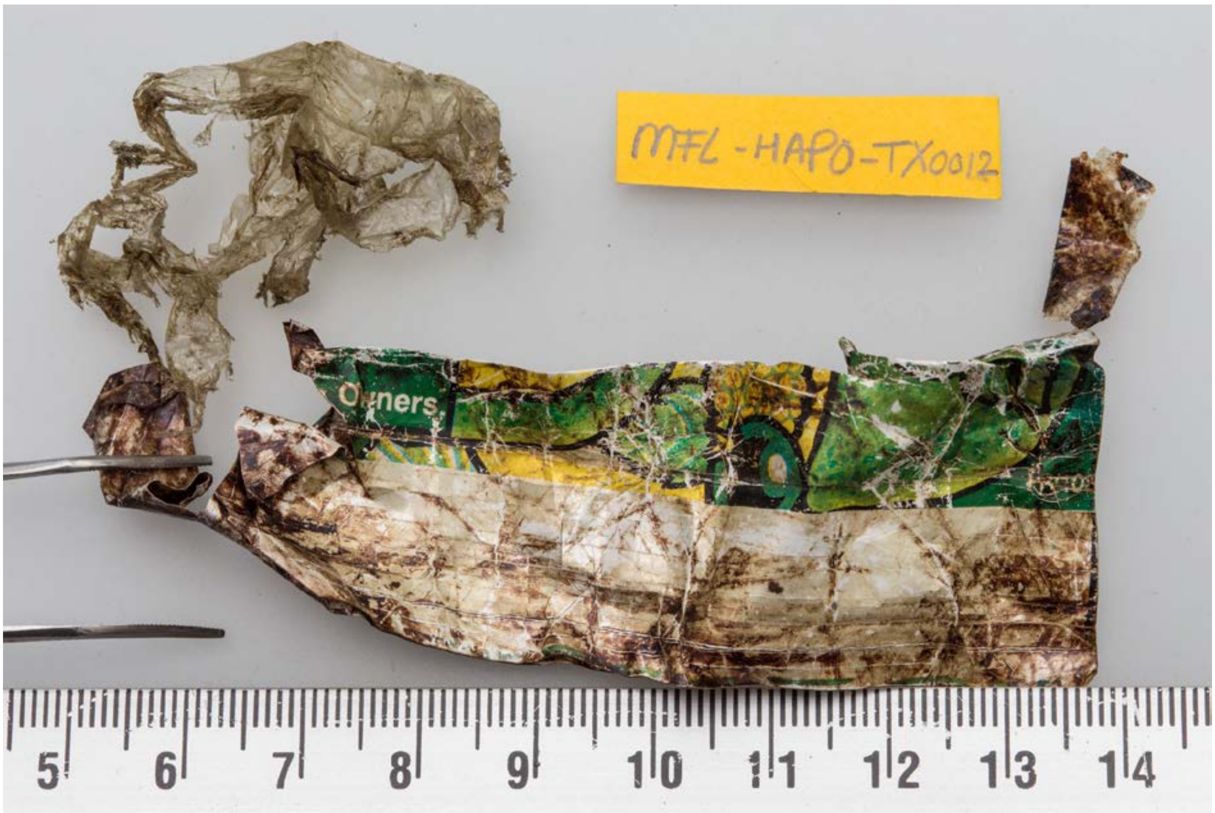

Transparent top left sheet PE (81%) Mass 0.0262 g;

Printed bottom sheet No ID (PP 50%) Mass 0.1399 g;

Dark top right sheet PP (91%); Mass 0.0049 g

MFL-HAPO-TX0038

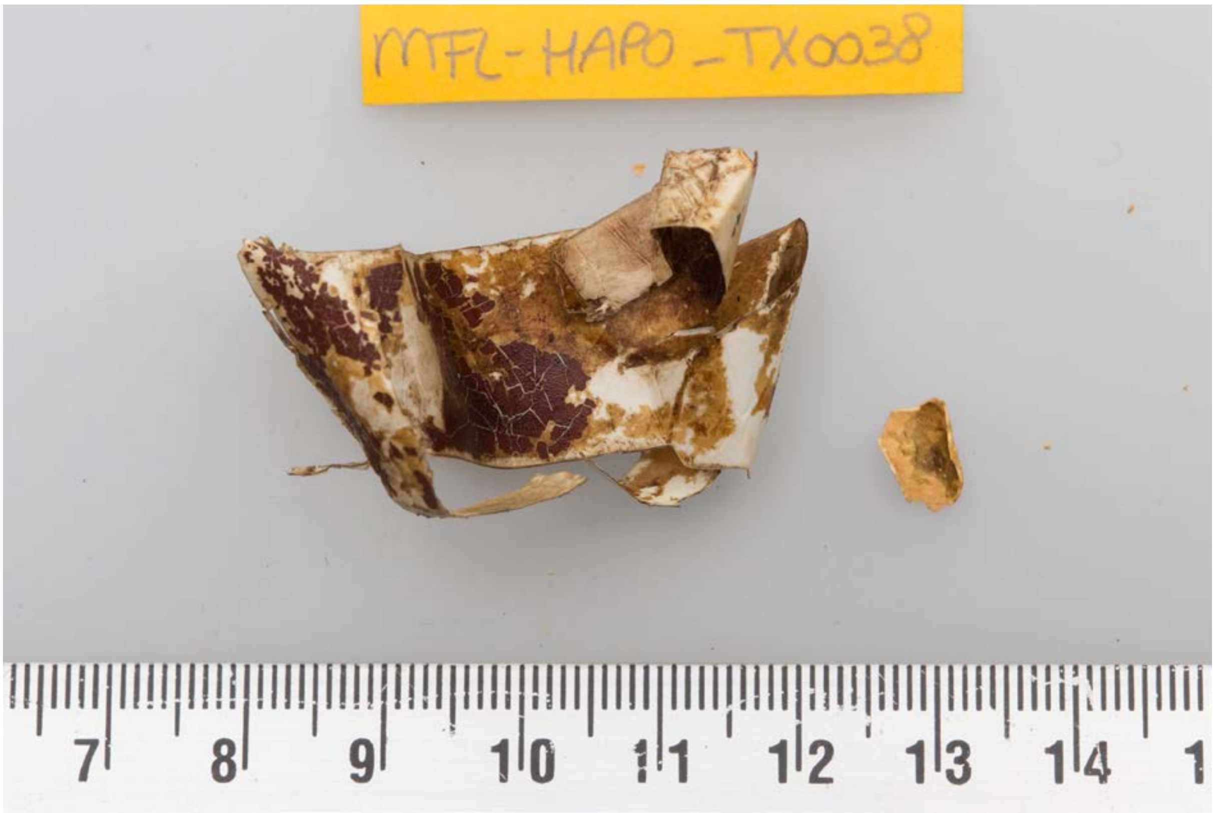

Sheet (left) No ID (PP 64%) Mass 0.1635 g;

other plastic (right) PVC (87%) Mass 0.0445 g

MFL-HAPO-TX0040

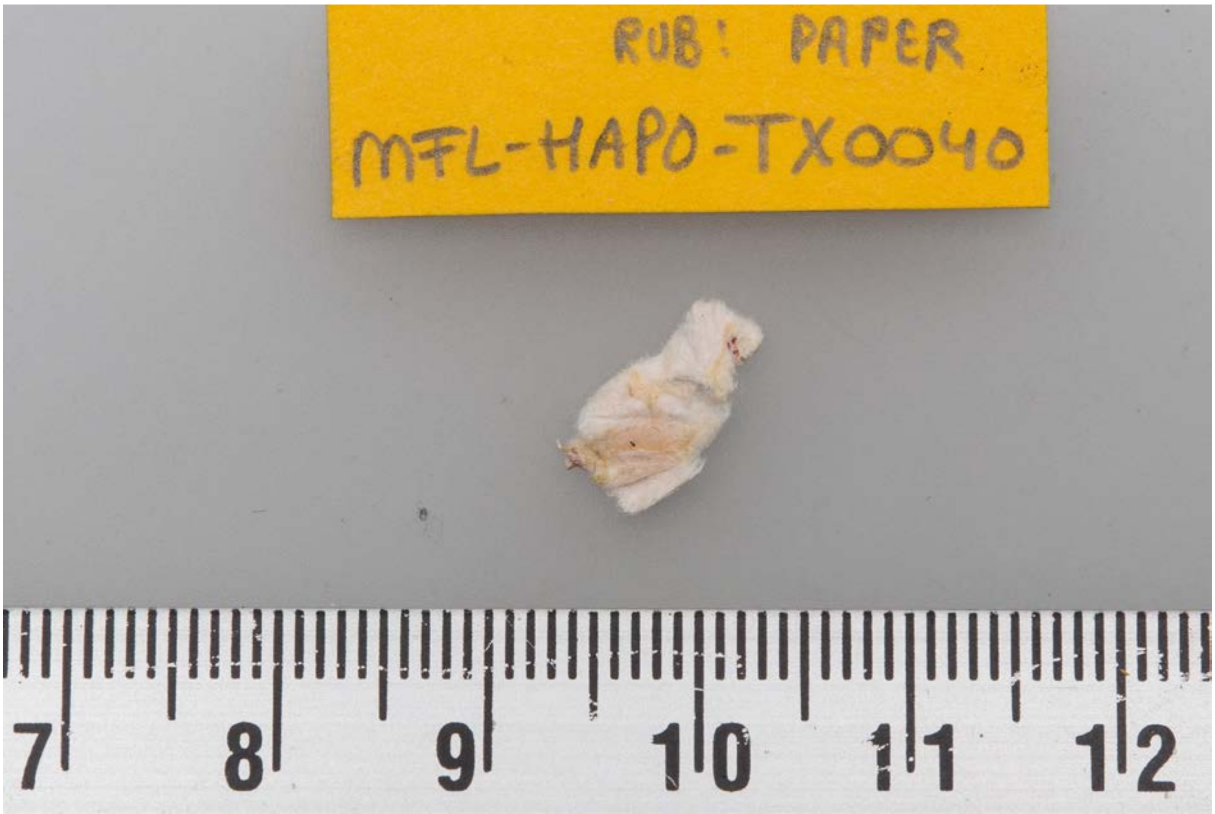

Not plastic but other rubbish:

Paper No ID (PET 68%) Mass 0.0051 g

MFL-HAPO-TX0049

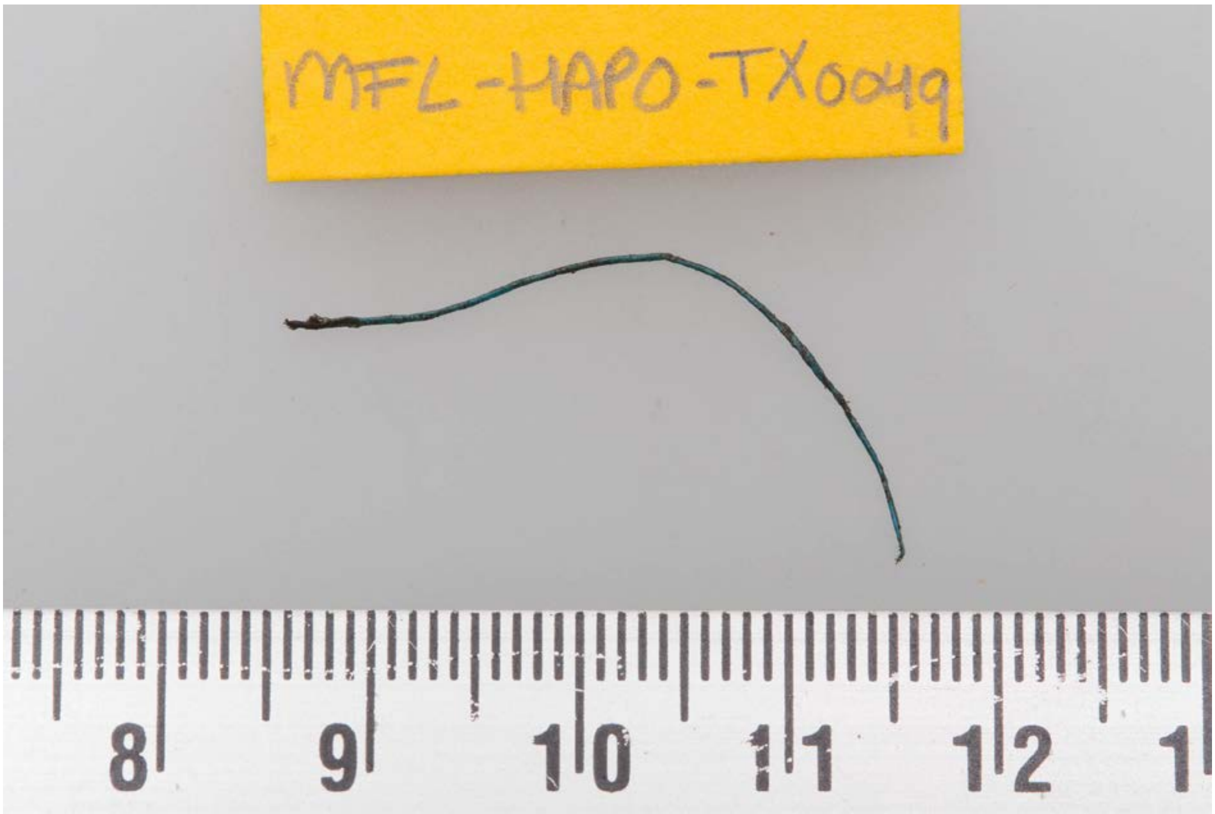

Thread PE (95%) Mass 0.0038 g

MFL-HAPO-UT0101

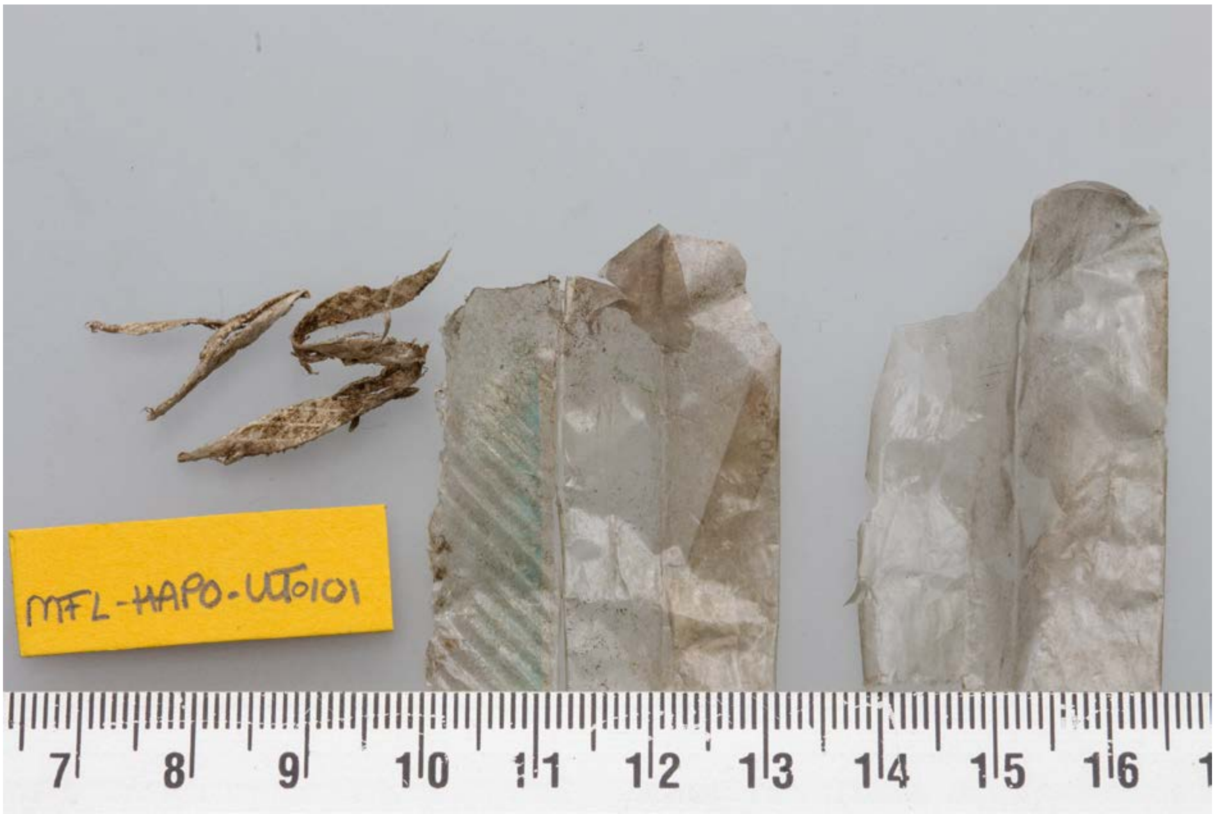

From left to right:

Sheet PP (87%) Mass 0.0142 g;

Sheet PP (87%) Mass 0.0367 g;

Sheet PP (97%) Mass 0.0365 g;

Sheet PP (97%) Mass 0.0480 g

MFL-HAPO-UT0108

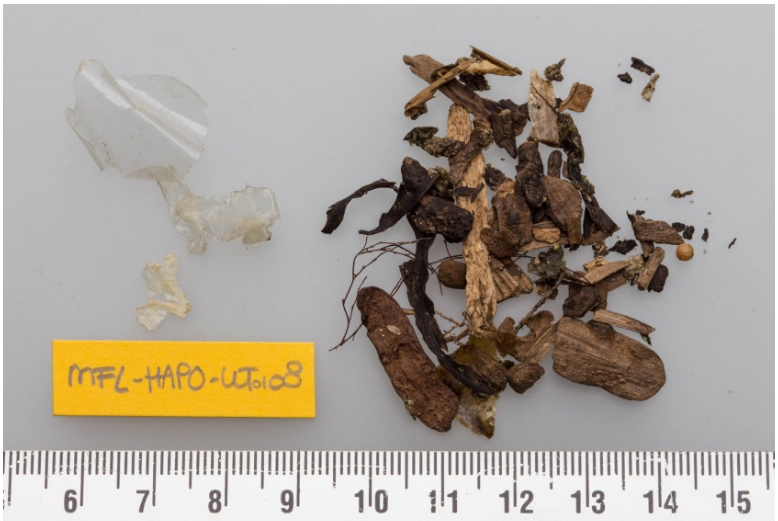

with natural items,
mainly smaller pieces of bog-wood

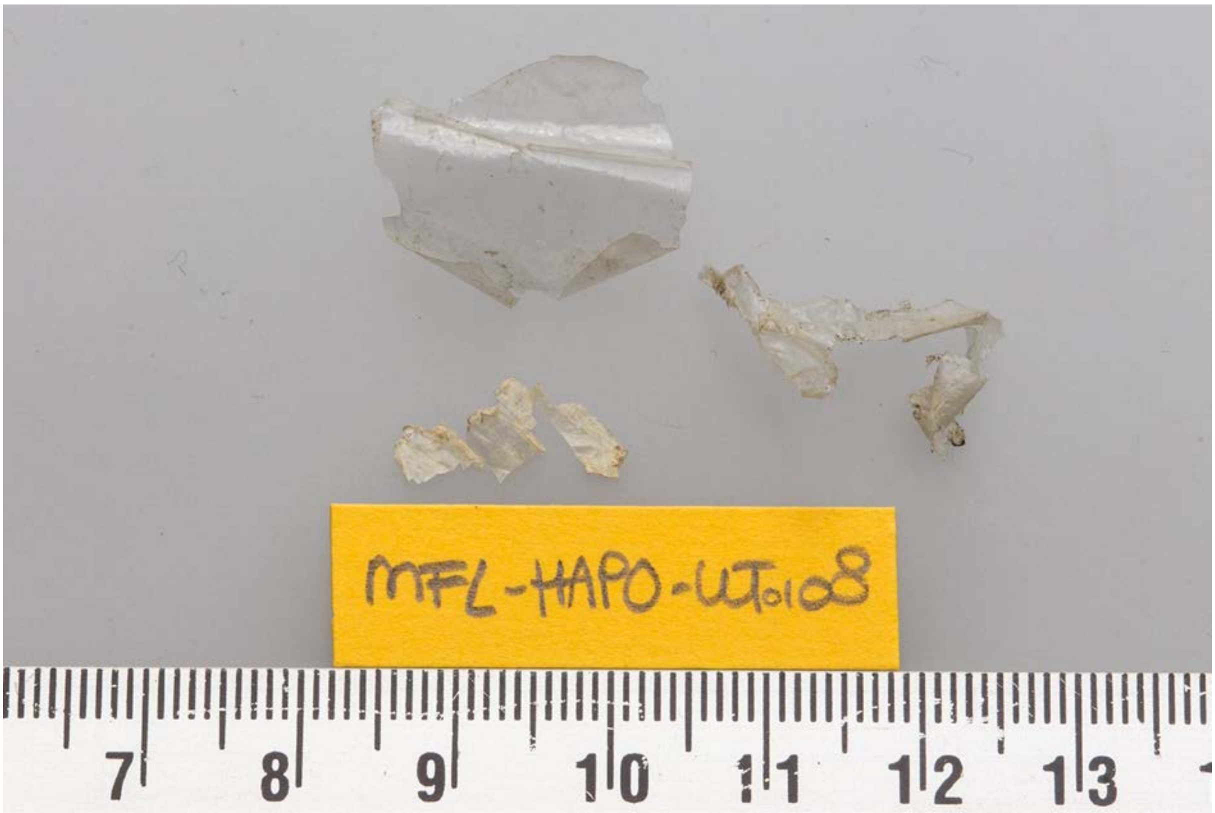

Top left Sheet PP (87%) Mass 0.0108 g;

Sheet bottom left No ID (PA 64%) Mass 0.0010 g;

Sheet bottom centre No ID (PA 64%) Mass 0.0001 g;

Bottom right Sheet PE (88%) Mass 0.0039 g

MFL-HAPO-UT0122

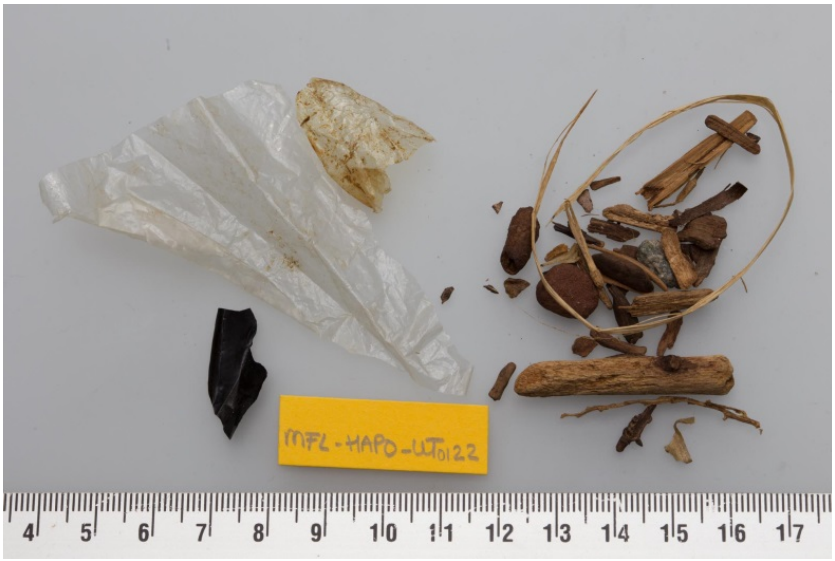

with natural items, mainly smaller pieces of bog wood

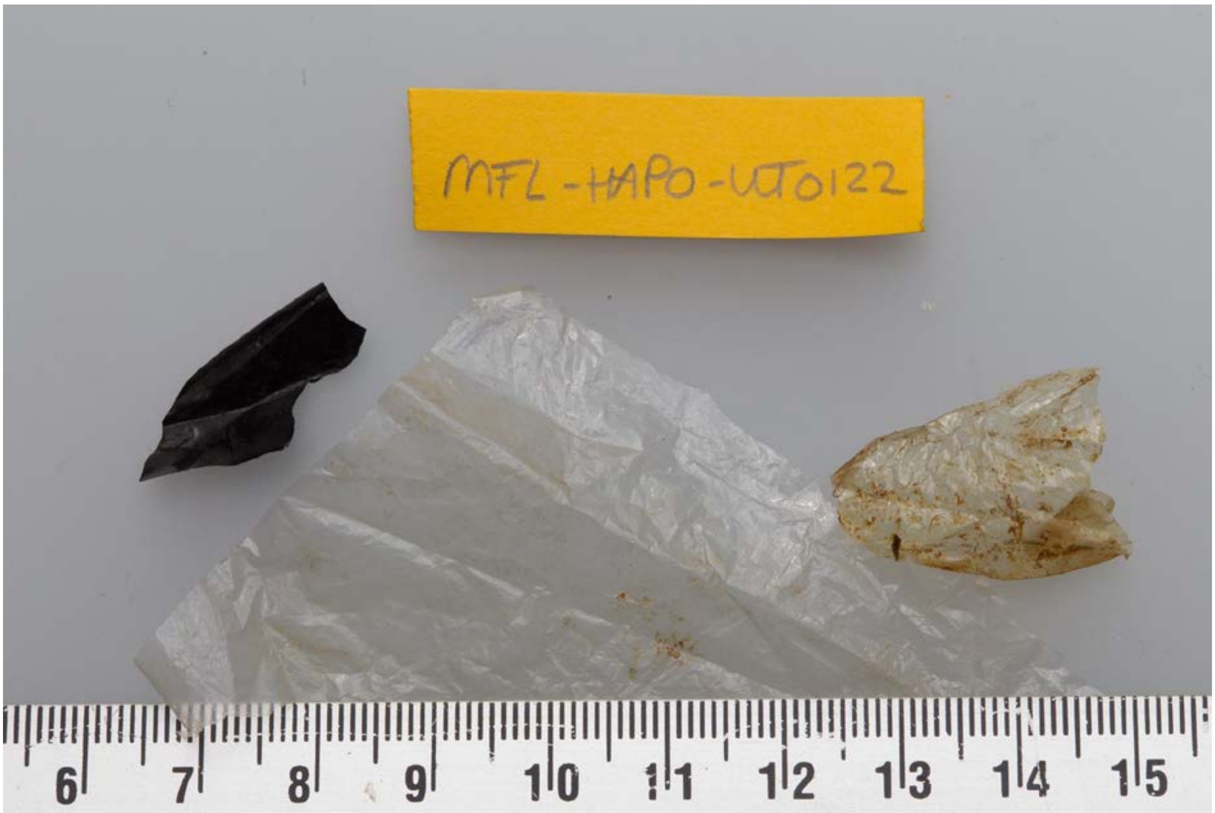

From left to right:

Sheet No ID (PS 15%) Mass 0.1510 g;

Sheet PET (89%) Mass 0.0524 g;

Sheet PVC (87%) Mass 0.0200 g

MFL-HAPO-UT0145

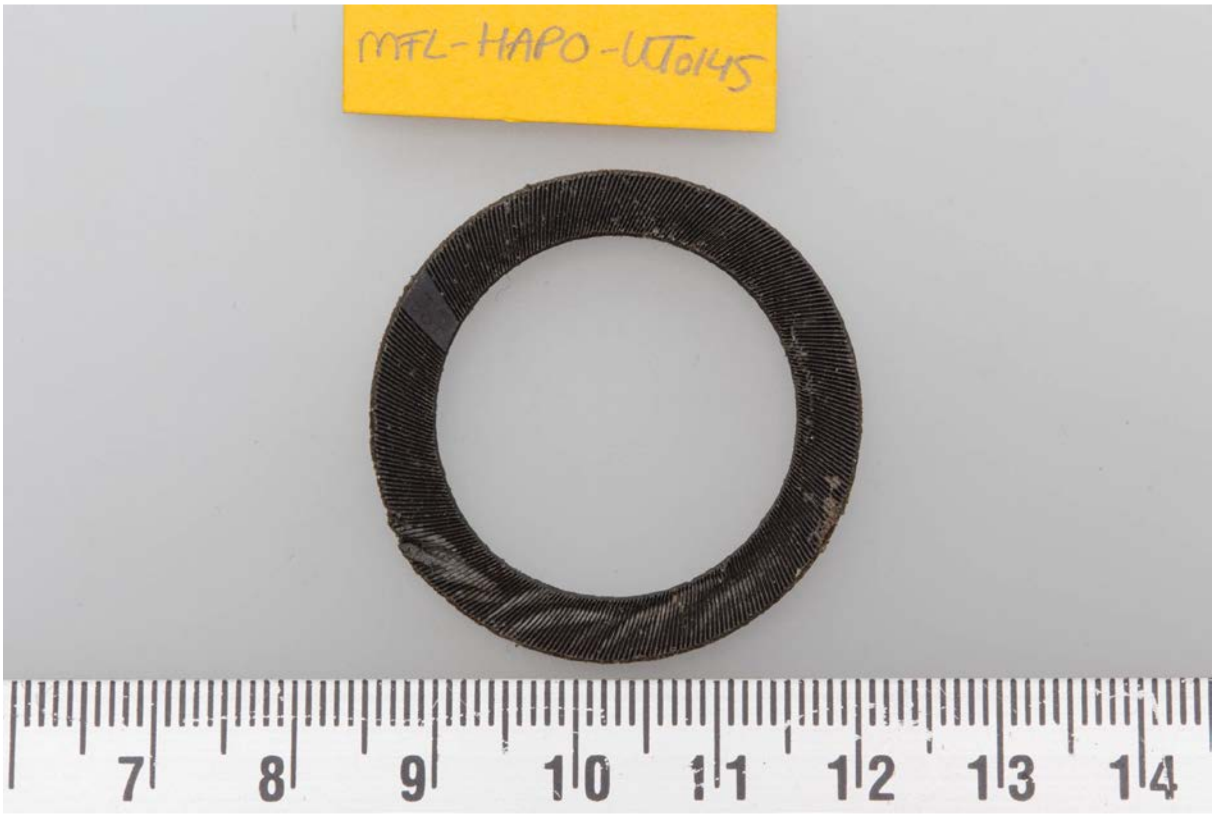

Other Plastic *(large rubbery like synthetic washer)*

no ID (PS 11%) Mass 0.3138 g

MFL-HAPO-UT0151

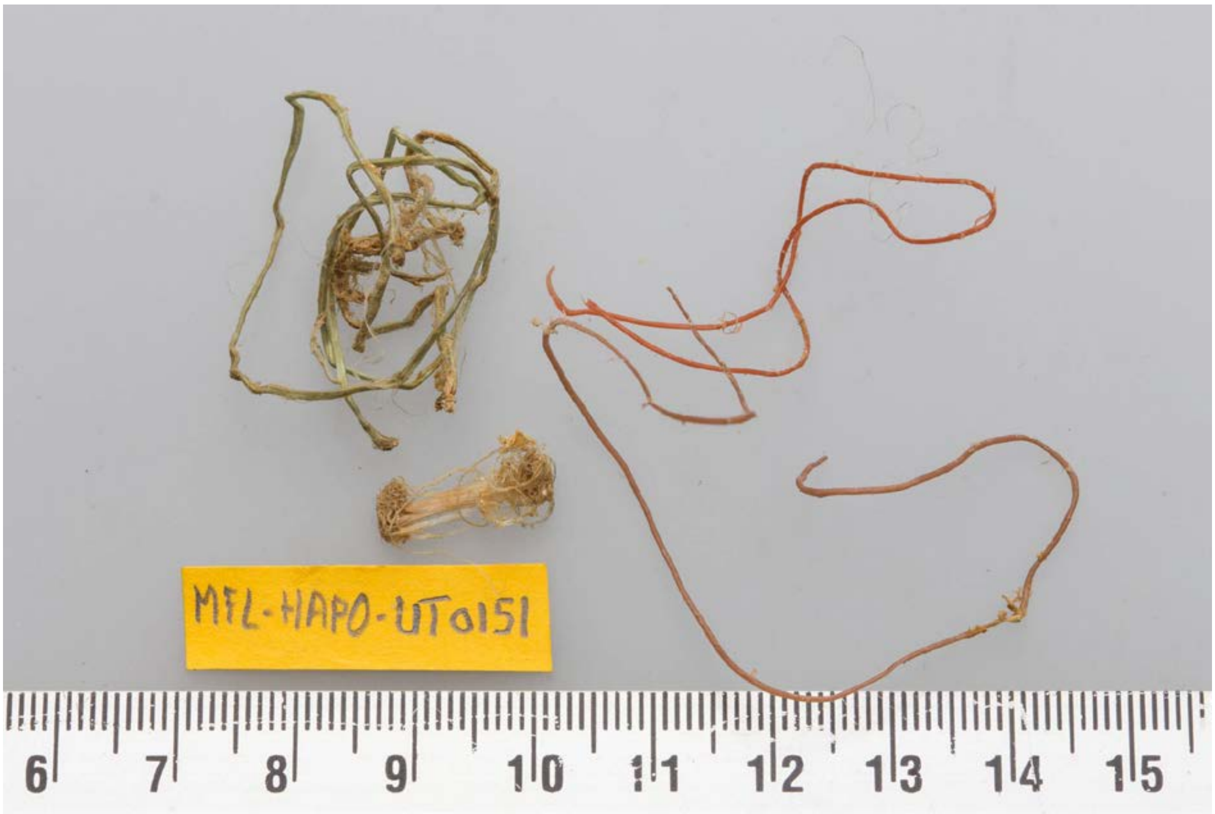

Top left thread PA (84%) Mass 0.0415 g;

Top left thread (entangled with previous) PP (92%) Mass 0.0344 g;

top right thread PE (97%) Mass 0.0168 g;

Bottom left thread PA (83%) Mass 0.0217 g;

Bottom right thread PE (92%) Mass 0.0304 g

MFL-HAPO-UT0153

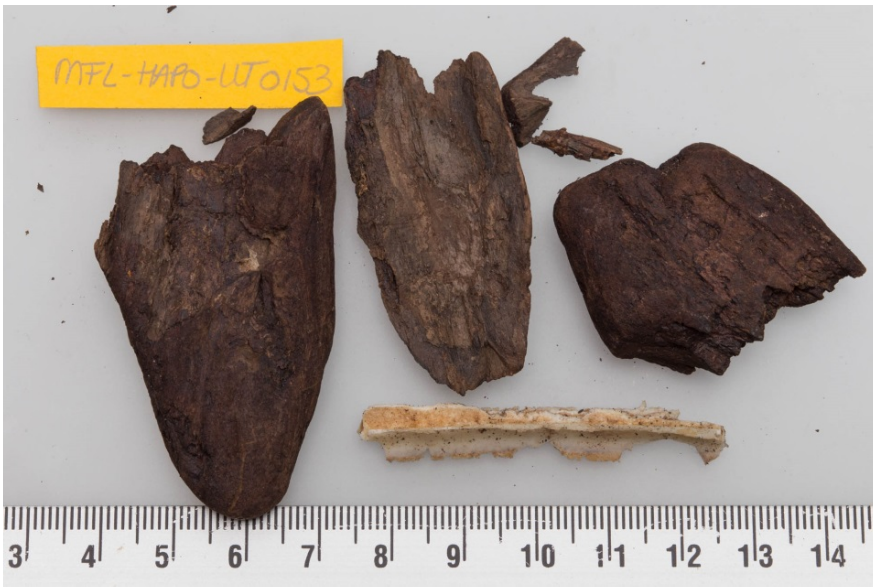

with natural items, large pieces of bog wood

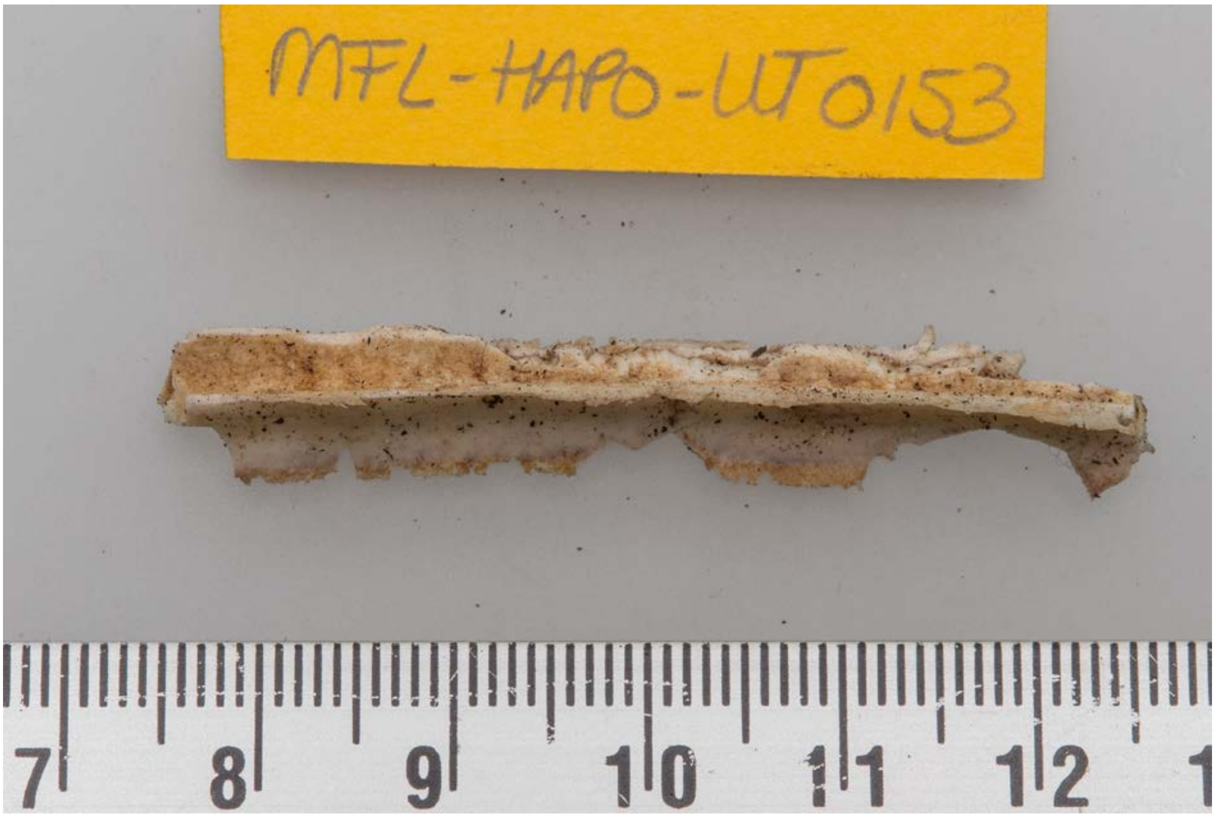

Other plastic No ID (POM 60%) Mass 0.6086 g

MFL-HAPO-UT0164

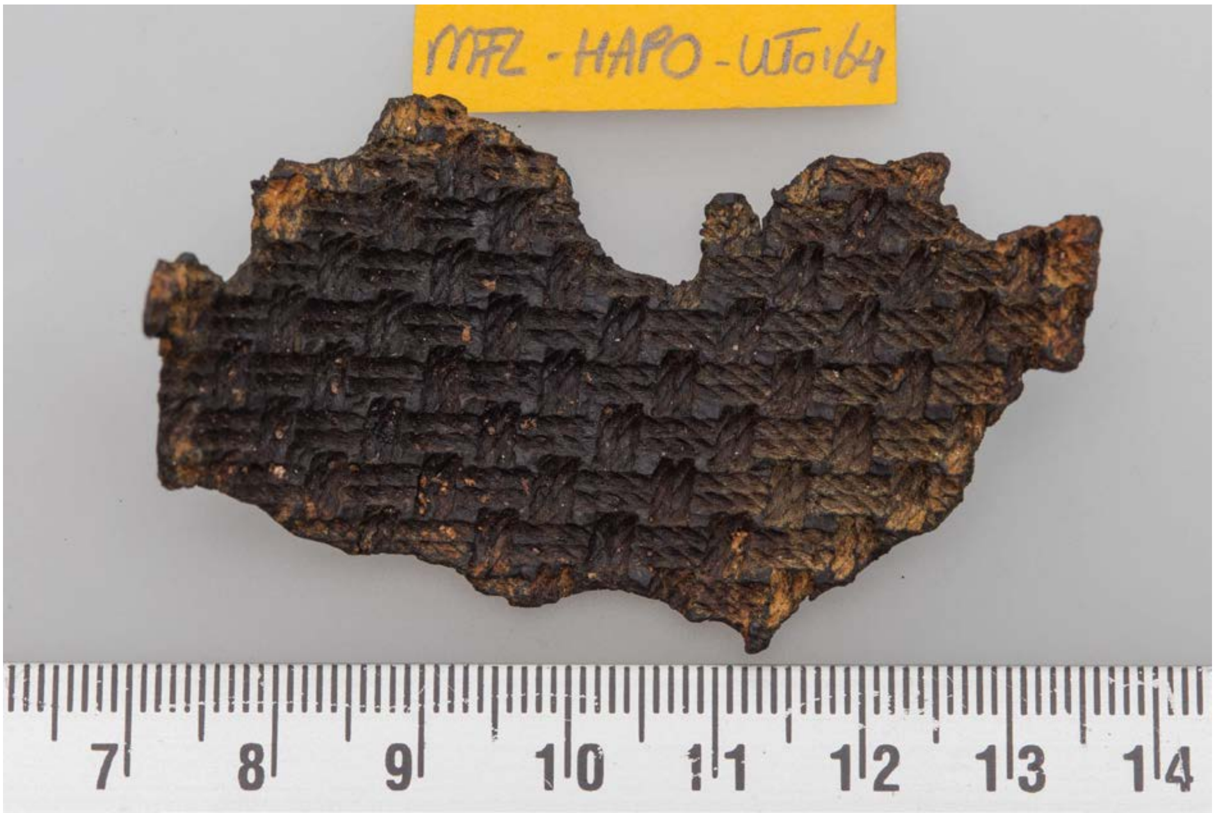

Other plastic No ID (PS 14%) Mass 2.5722 g

MFL-HAPO-UT0177

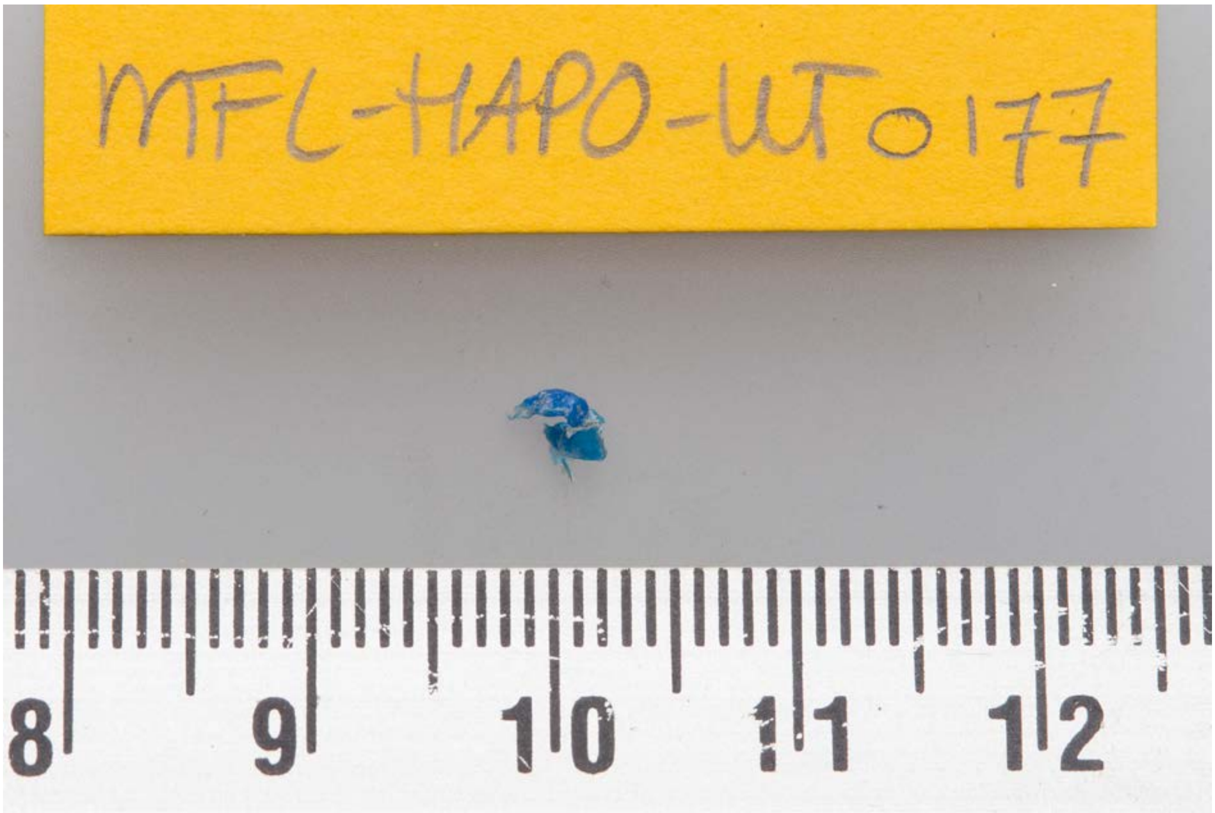

Fragment PE (98%) Mass 0.0037 g

MFL-HAPO-UT0180

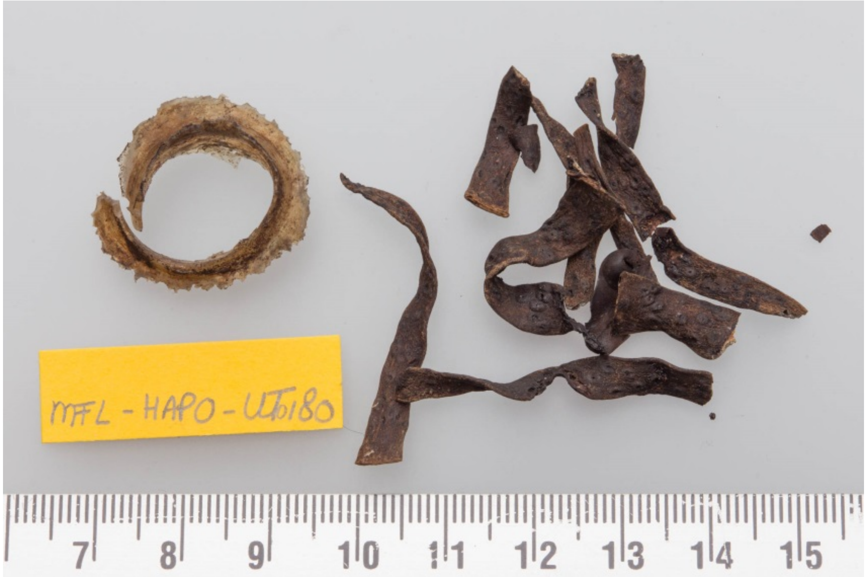

with natural items
seaweed

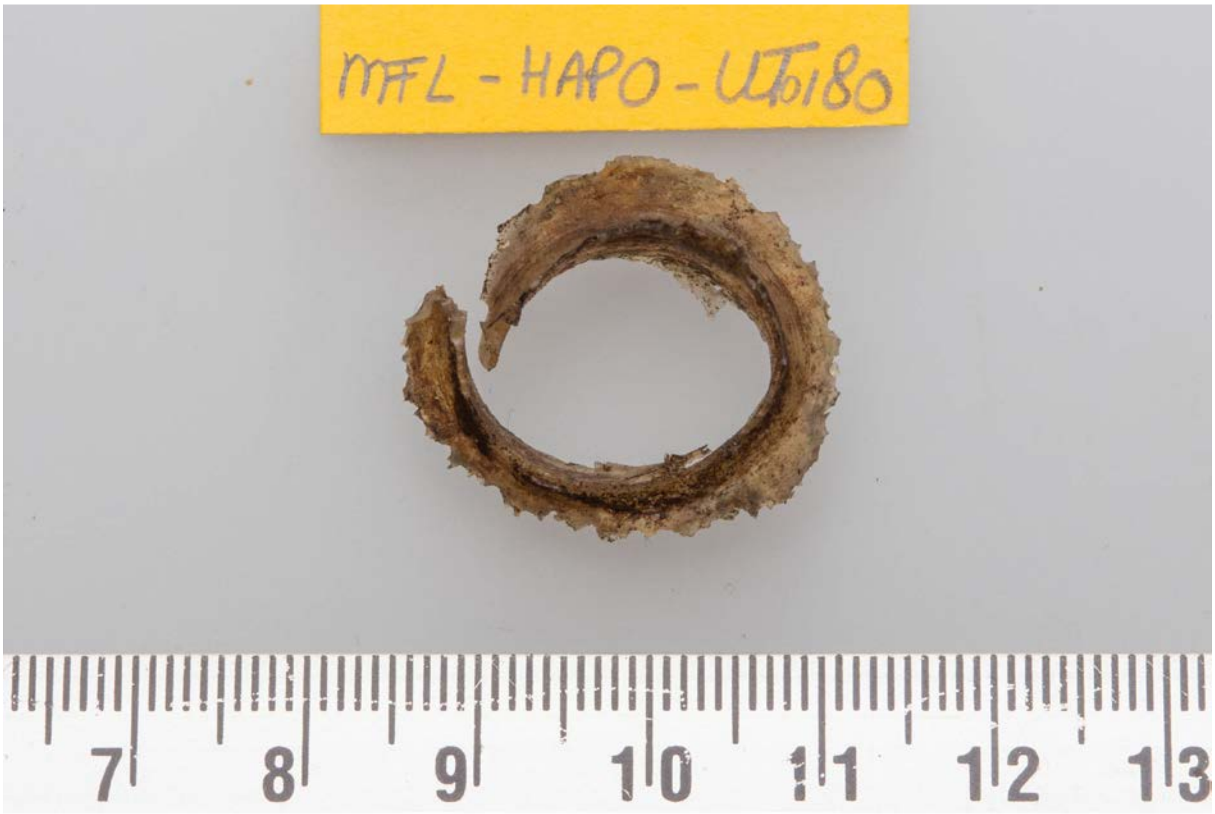

Other plastic No ID (POM 60%) Mass 0.6596 g

MFL-HAPO-UT0193

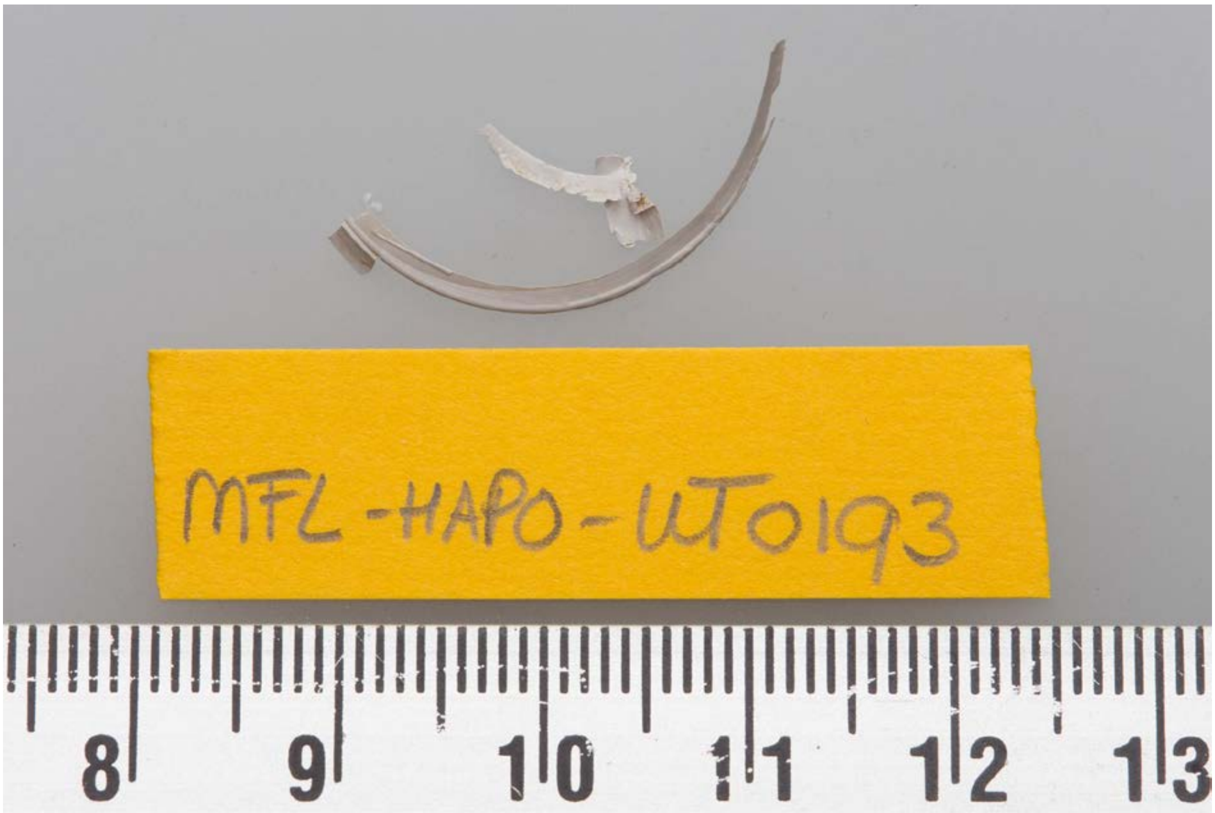

Fragment No ID (PSO 63%) Mass 0.0059 g

MFL-HAPO-UT0197

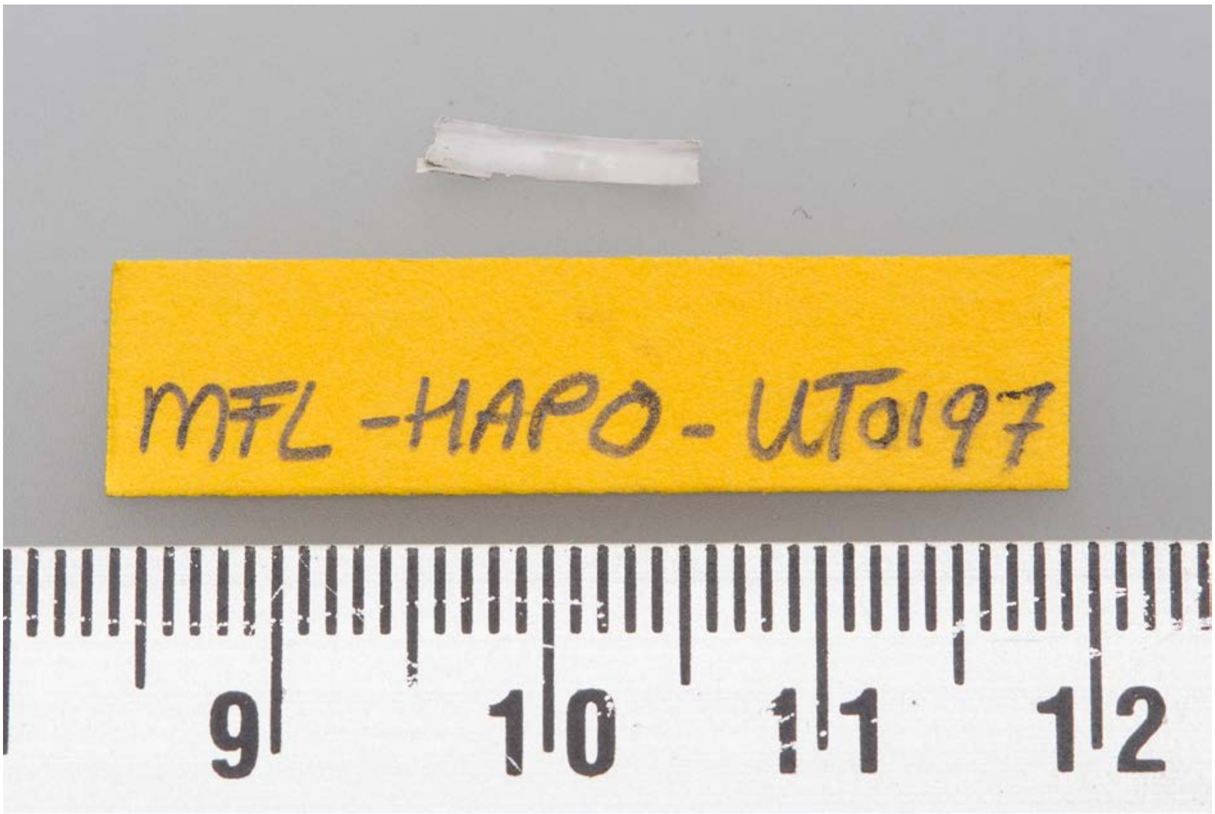

Fragment No ID (PET 72%) Mass 0.0023 g

MFL-HAPO-UT0201

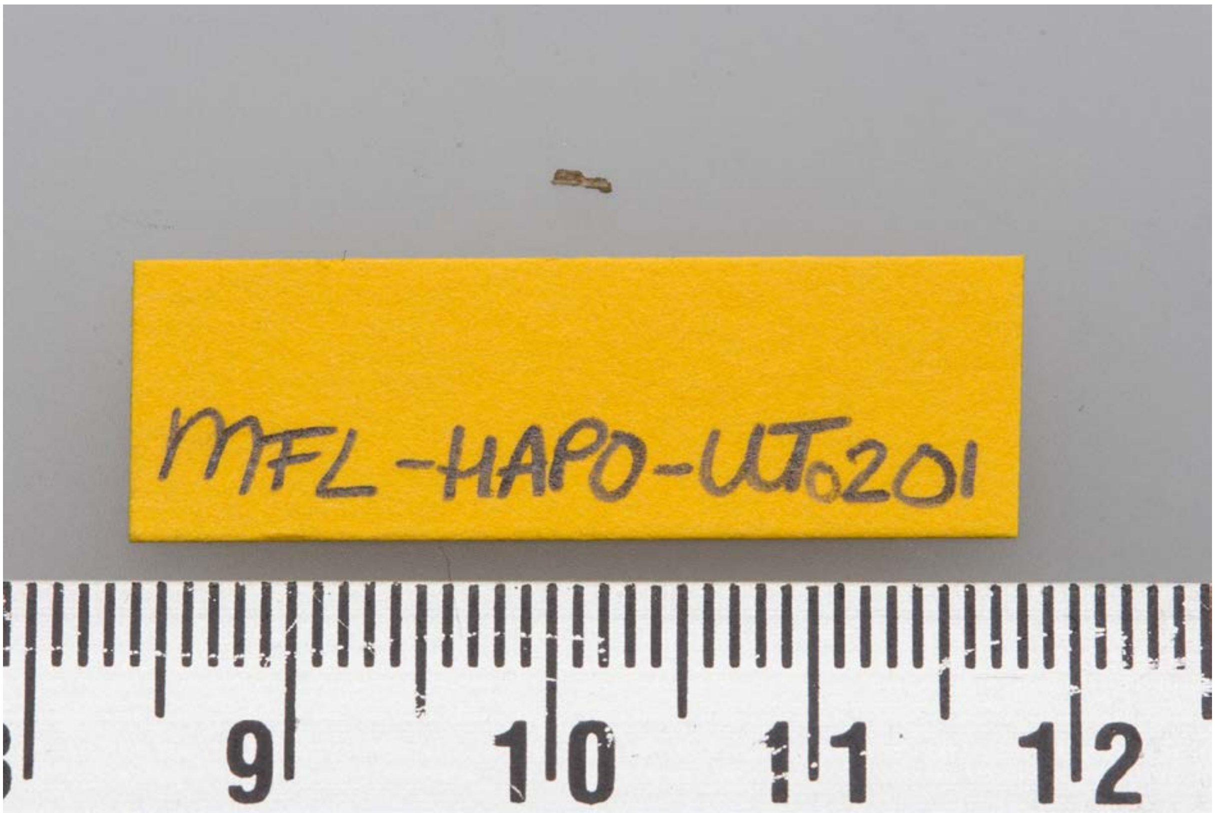

Fragment No ID (PA/ABS 46%) Mass 0.0003 g

MFL-HAPO-UT0208

no photo, because sample lost

(1 sheet estimated 0.001 g)

No NIR analysis done

MFL-HAPO-UT0210

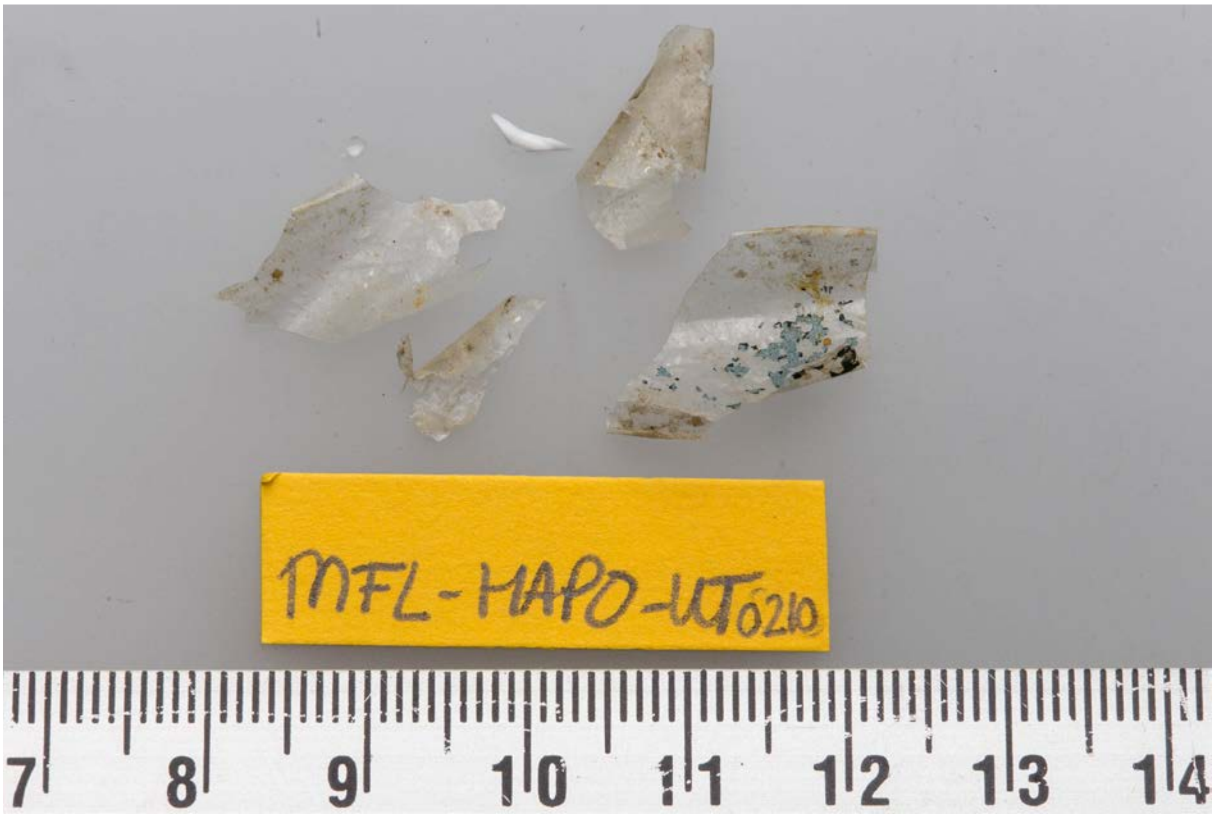

Top left sheet PP (90%) Mass 0.0035gr;

Top right sheet PP (91%) Mass 0.0040 g;

Bottom left sheet PP (89%) Mass 0.0015 g;

Bottom right sheet PP (92%) Mass 0.0063 g

MFL-HAPO-UT0221

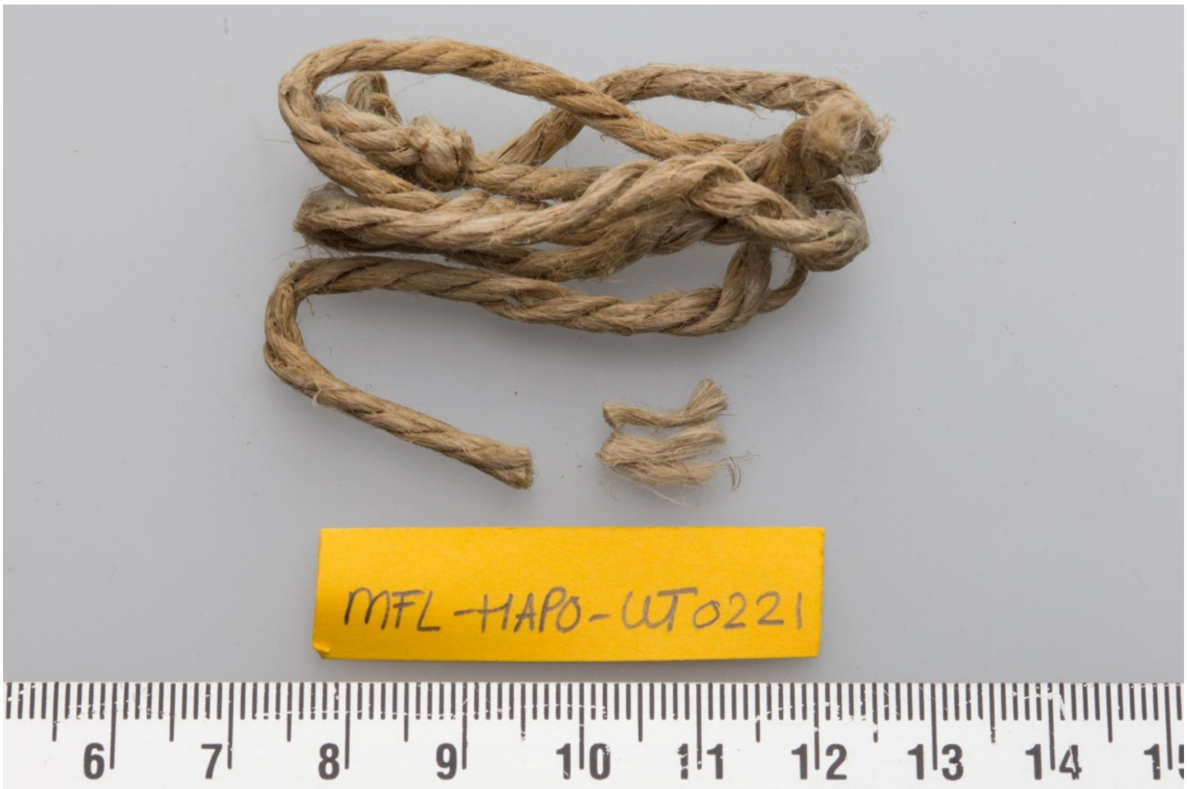

Other rubbish: Non-synthetic rope (broken during processing) No ID (PA 46%) Mass 1.1426 g

MFL-HAPO-UT0232

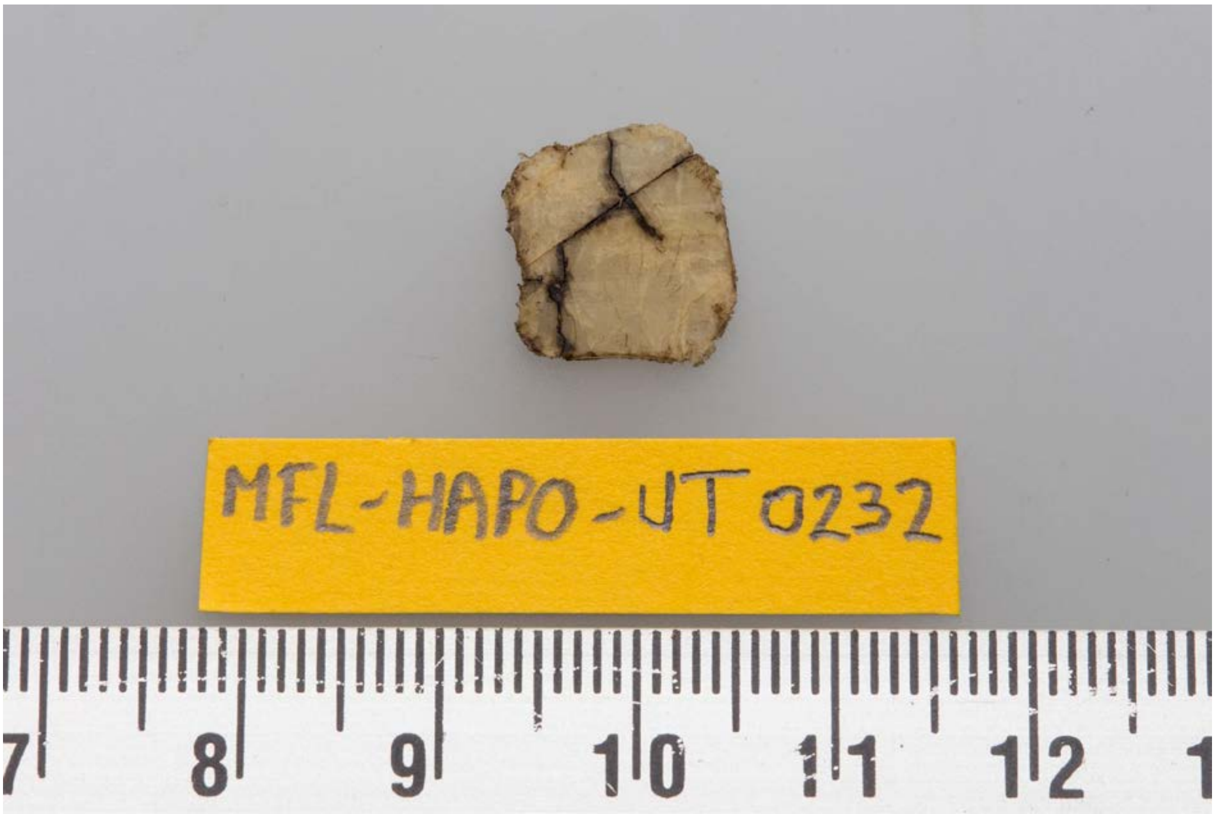

No NIR analysis done *(glass fibre like appearance)*

MFL-HAPO-UT0238

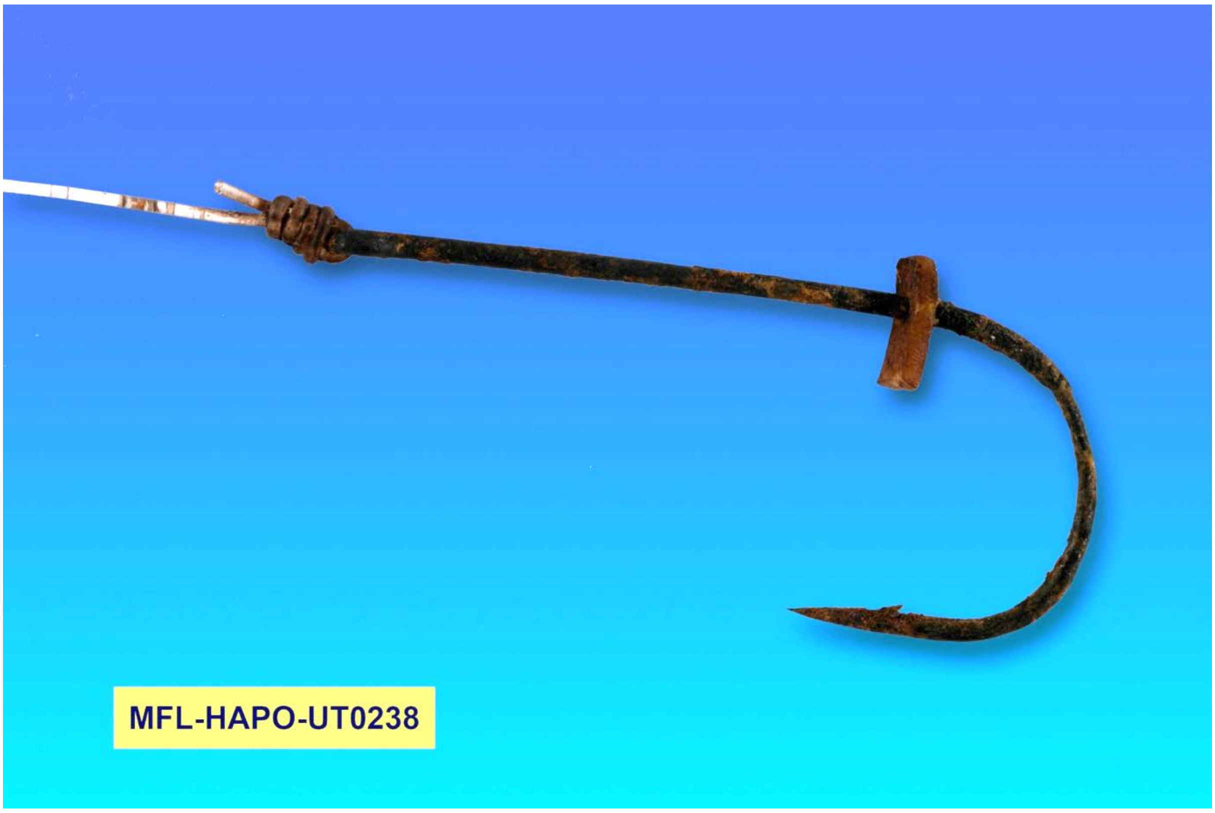

This item was recorded as both plastic for the nylon thread as under other rubbish for the metal hook. No NIR analysis done

MFL-HAPO-UT0348

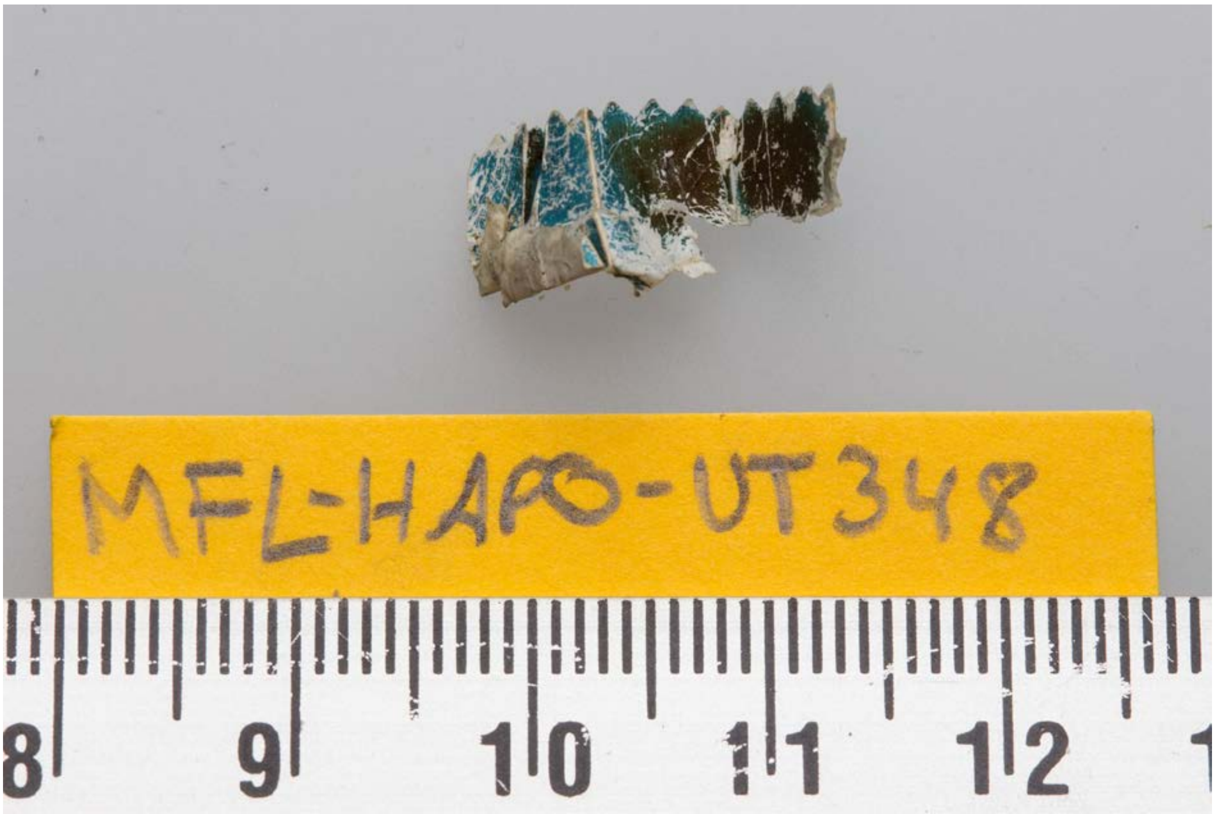

No NIR analysis done Mass 0.0114 g

MFL-HAPO-UT0360

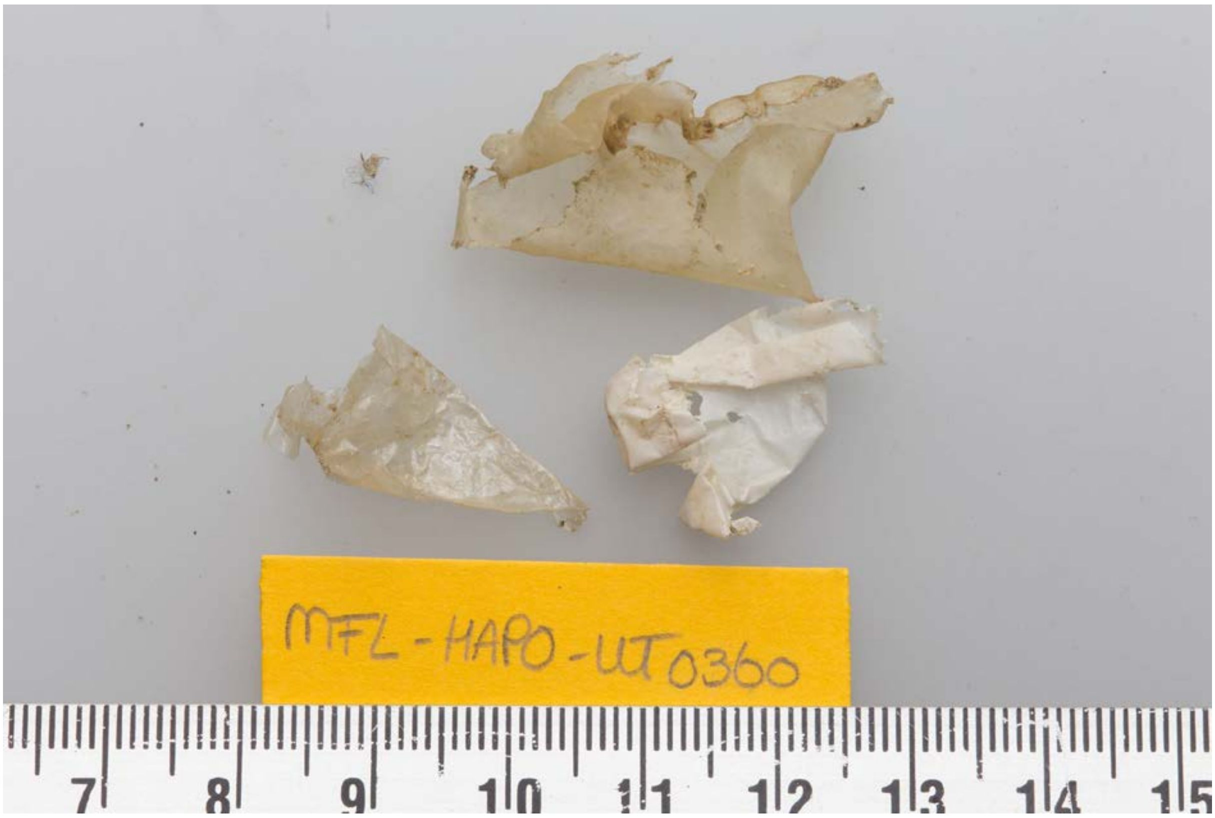

Top left thread No ID (PET 54%) Mass 0.0004;

Top right sheet PE (94%) Mass 0.0894 g;

Bottom left sheet No ID (PA 67%) Mass 0.0083 g;

Bottom right sheet PE (89%) Mass 0.0123 g

MFL-HAPO-UT0384

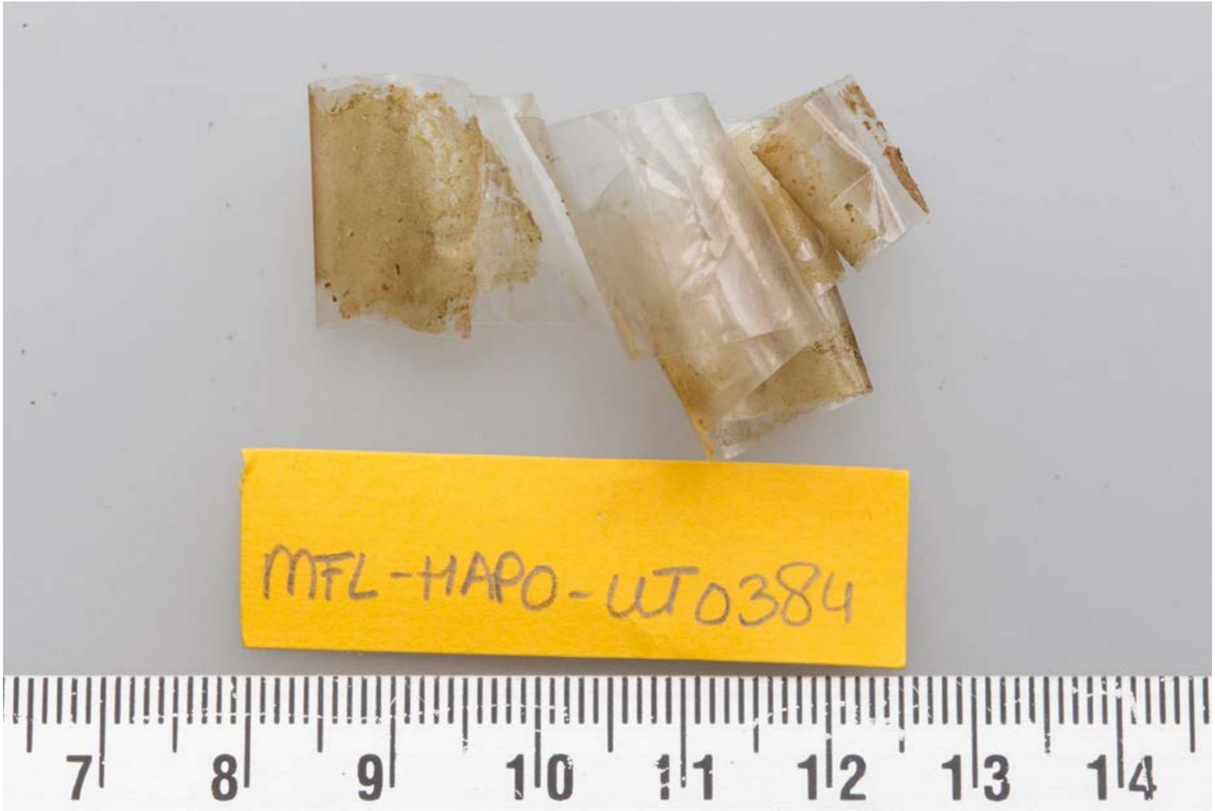

Sheet PP (90%) Mass 0.0083 g

MFL-HAPO-UT0413

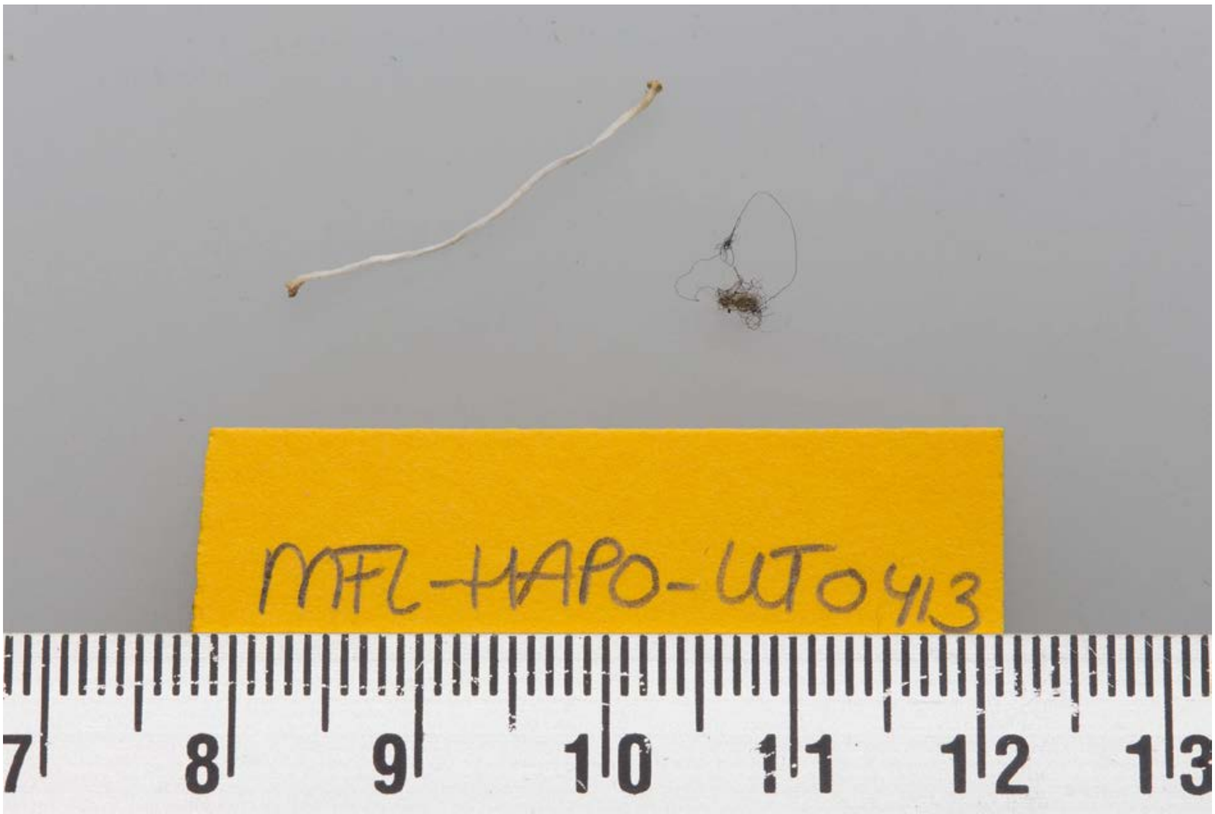

Left thread detected during overflow for the diet study; ball of threads (fibres) only detected later during analysis of the sieved remains.

Left thread PE (95%) Mass 0.0021 g;

right thread No ID (PA 46%) Mass 0.0001 g

MFL-HAPO-UT0435

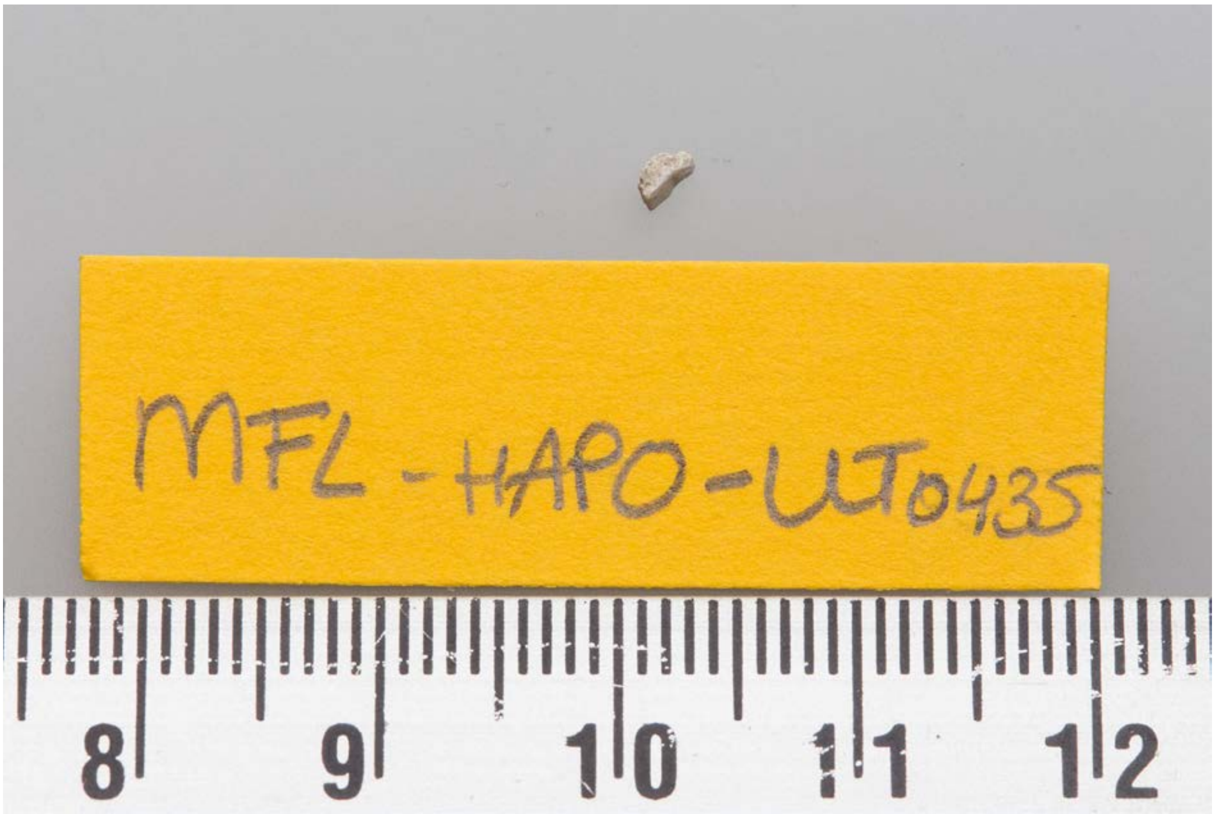

No plastic detected during the diet overflow procedure; this item found in the sieved remains.

Fragment No ID (PA 73%) Mass 0.0074 g

MFL-HAPO-UT0444

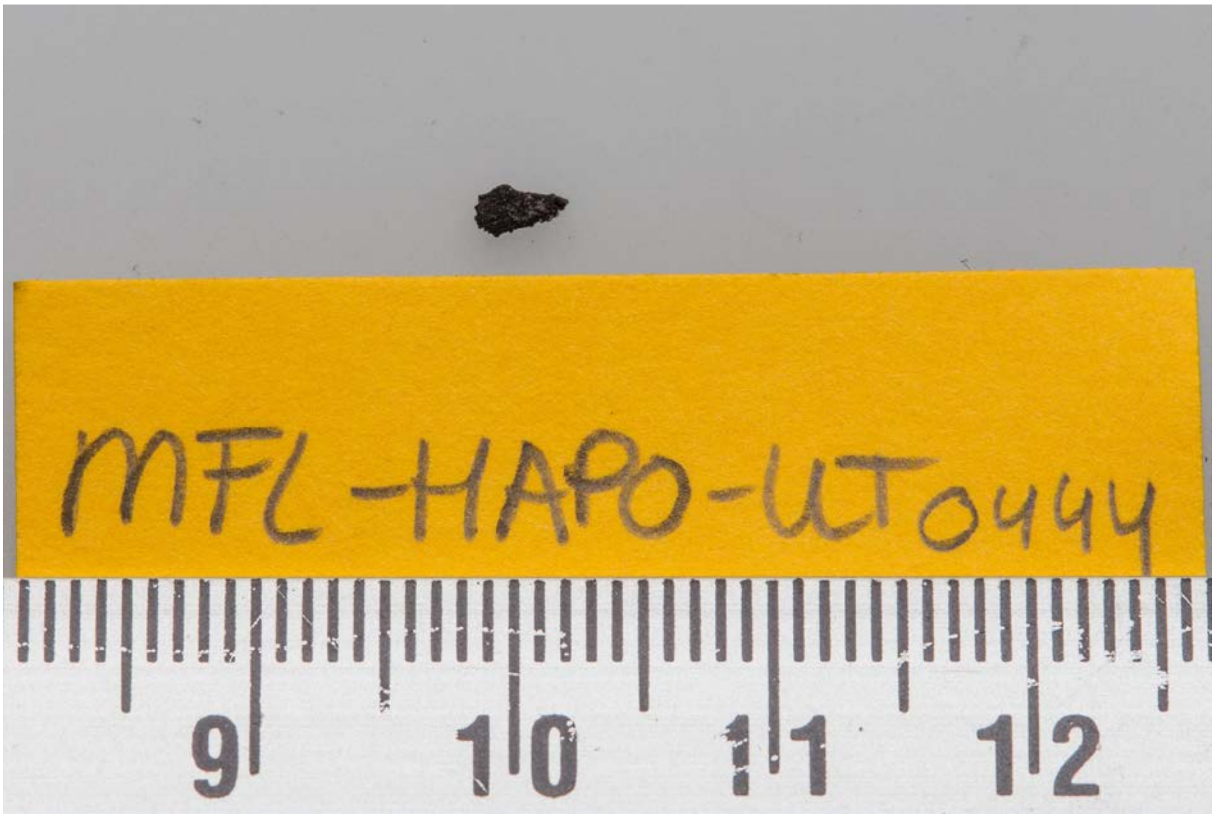

Fragment No ID (PA 69%) Mass 0.0037

MFL-HAPO-UT0471

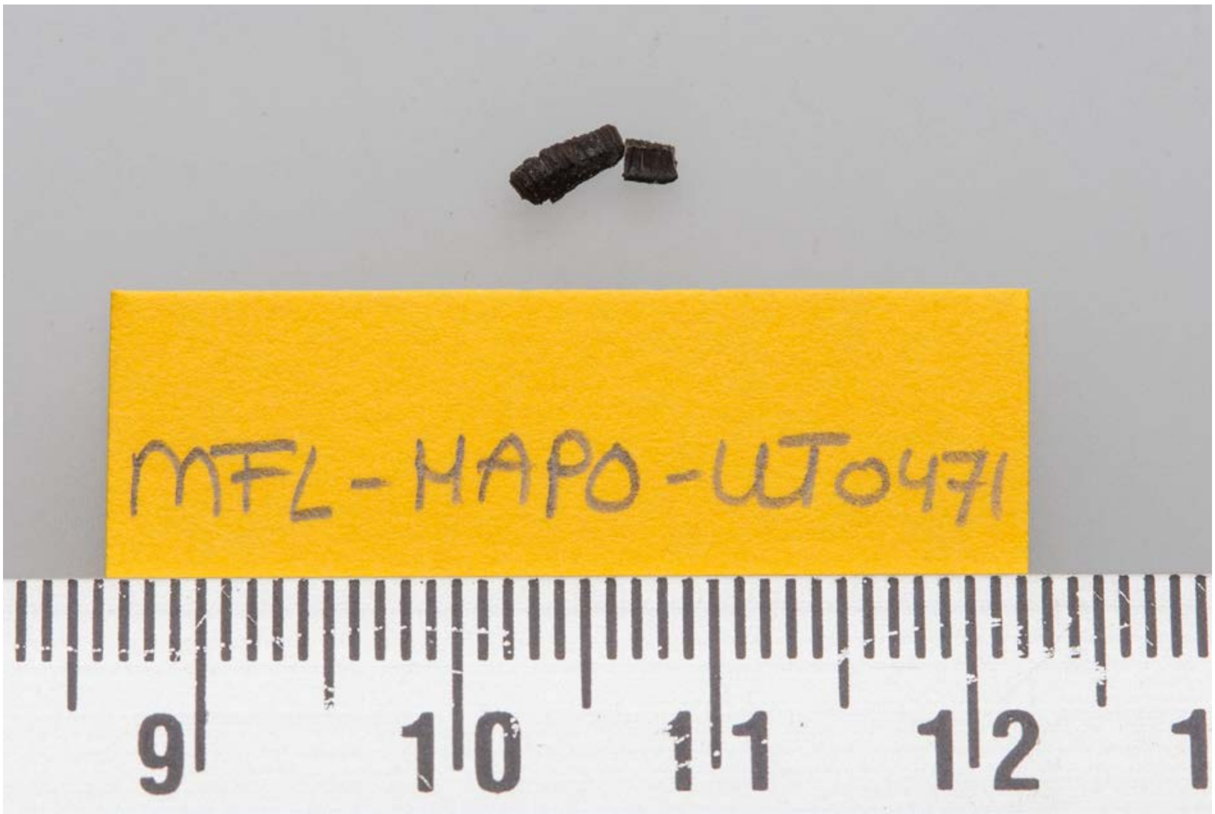

Fragment No ID (PA/ABS 68%) Mass 0.0071 g

MFL-HAPO-UT0510

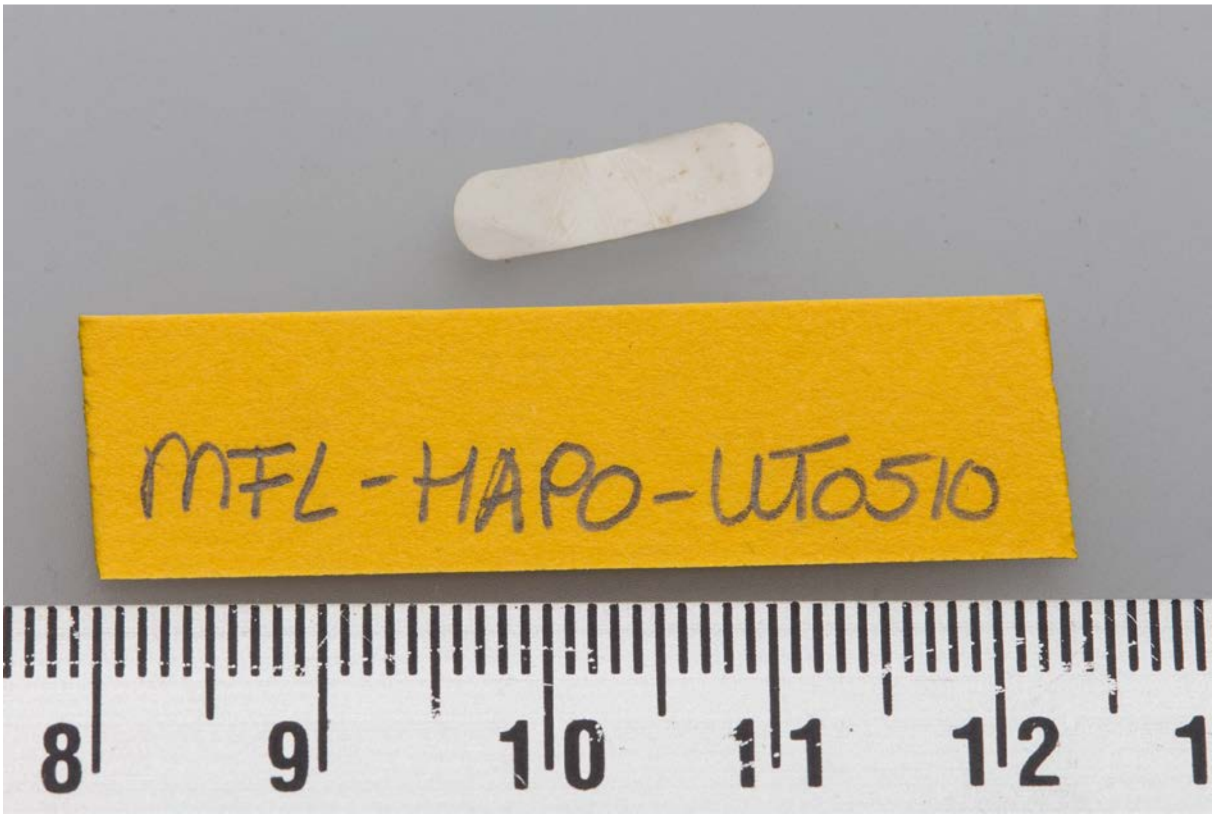

Sheet No ID (PP 56%) Mass 0.0041

MFL-HAPO-UT0511

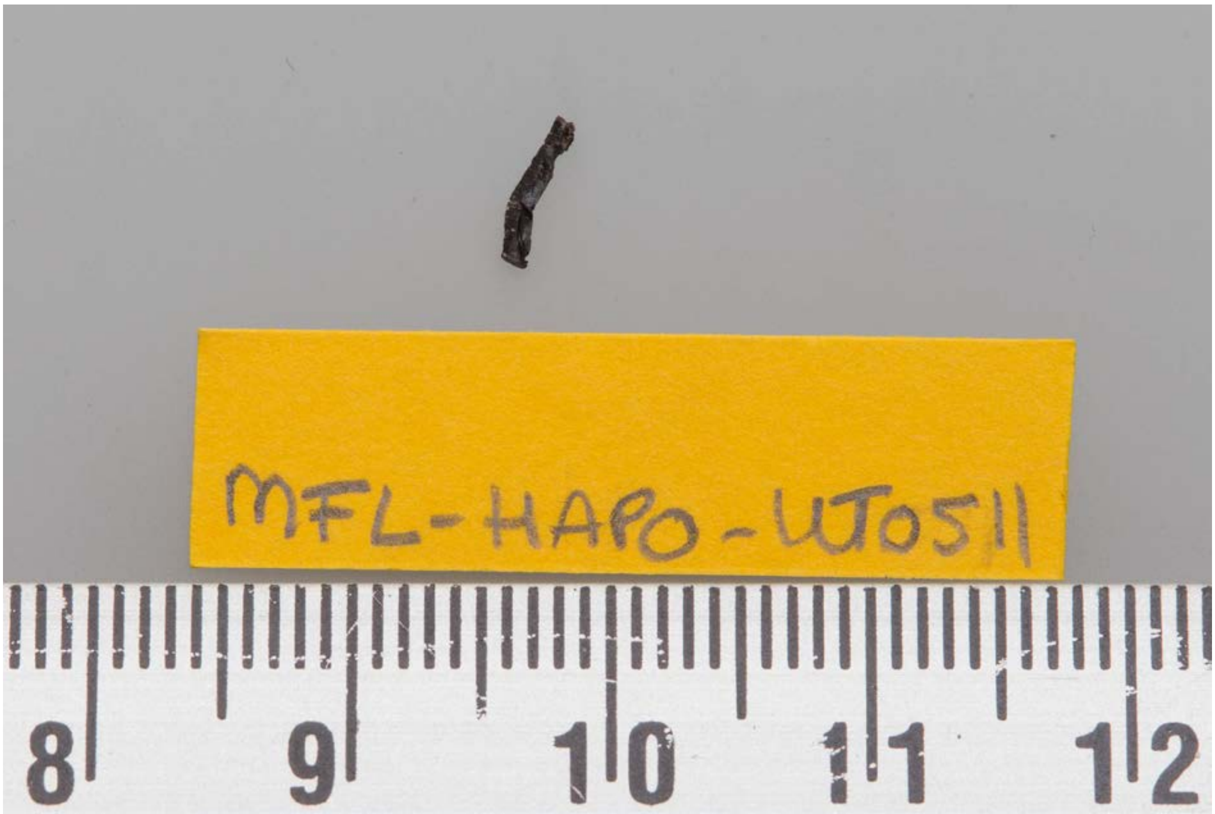

Fragment No ID (PA 68%) Mass 0.0013 g

MFL-HAPO-UT0516

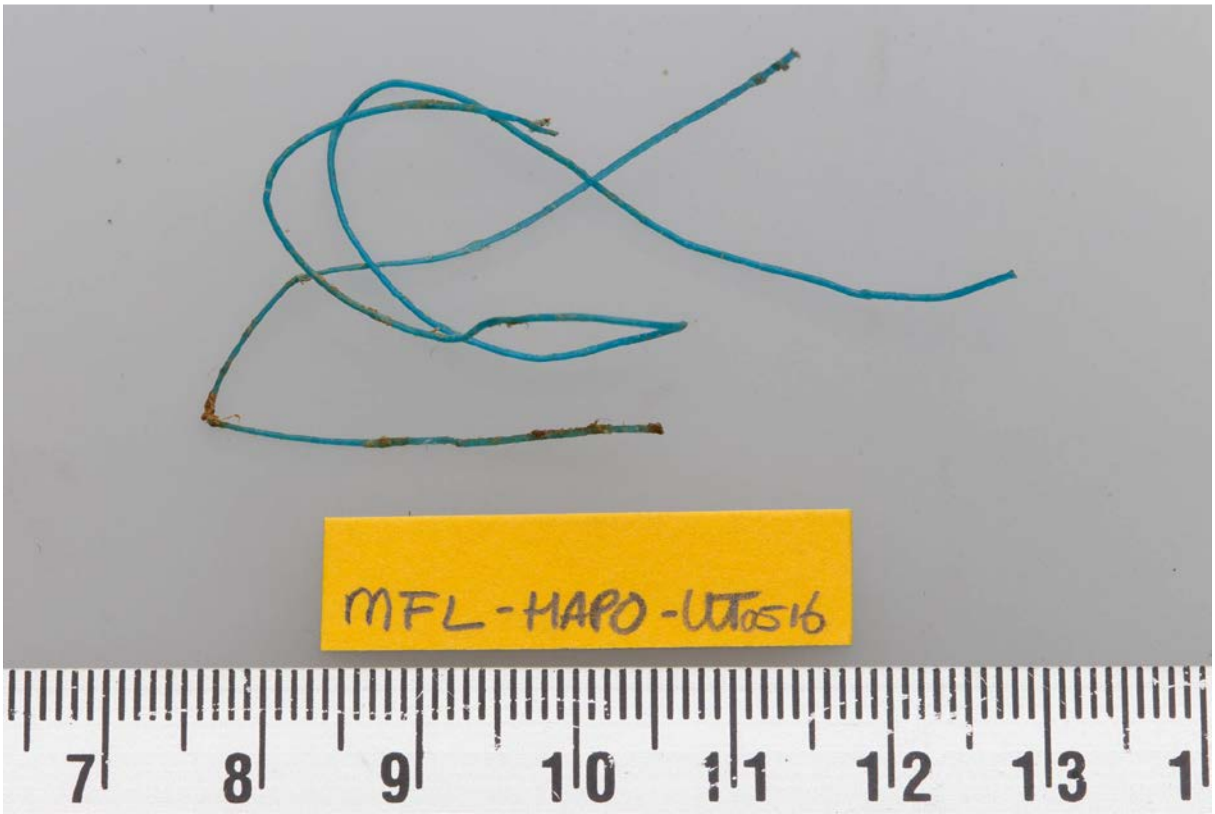

Left thread PE (94%) Mass 0.0104 g;

Right thread PE (95%) Mass 0.0231 g

MFL-HAPO-UT0532

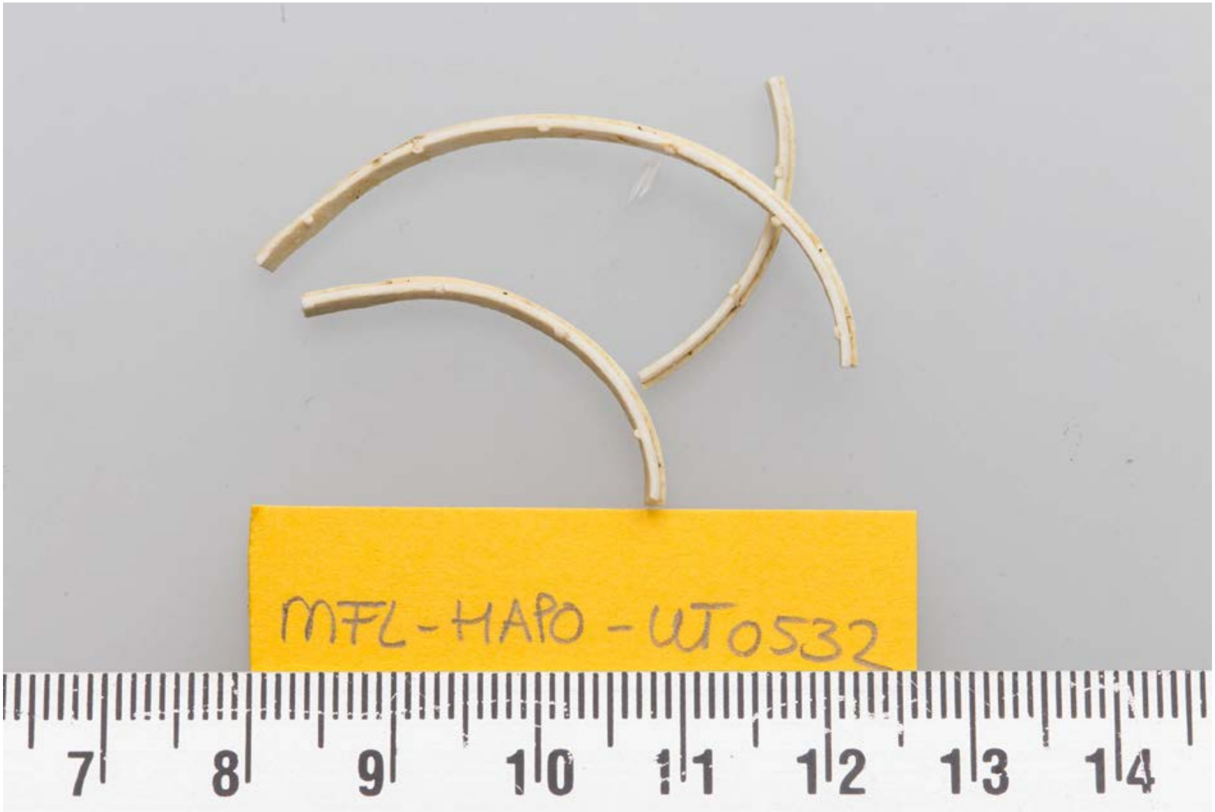

Fragment PE (95%) Mass 0.2457 g

MFL-HAPO-UT0605

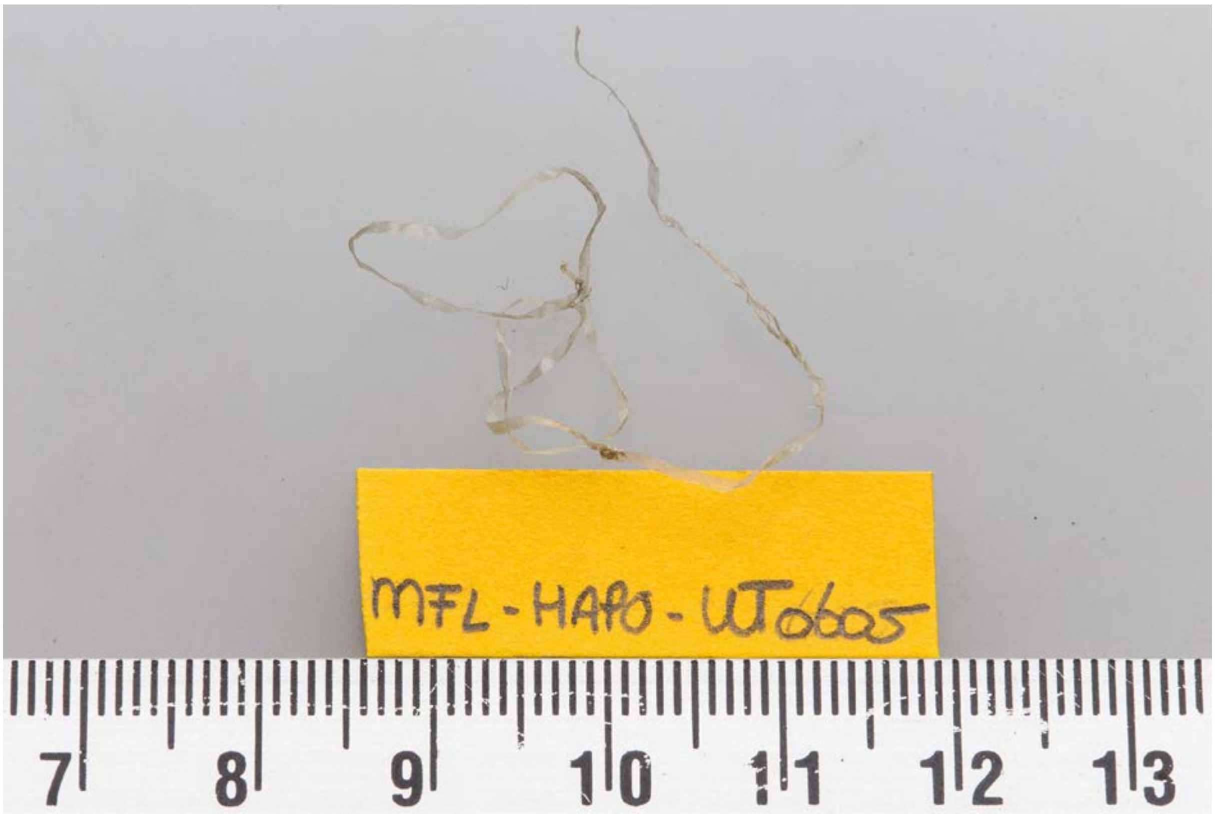

Thread PE (90%) Mass 0.0032 g

MFL-HAPO-UT0617

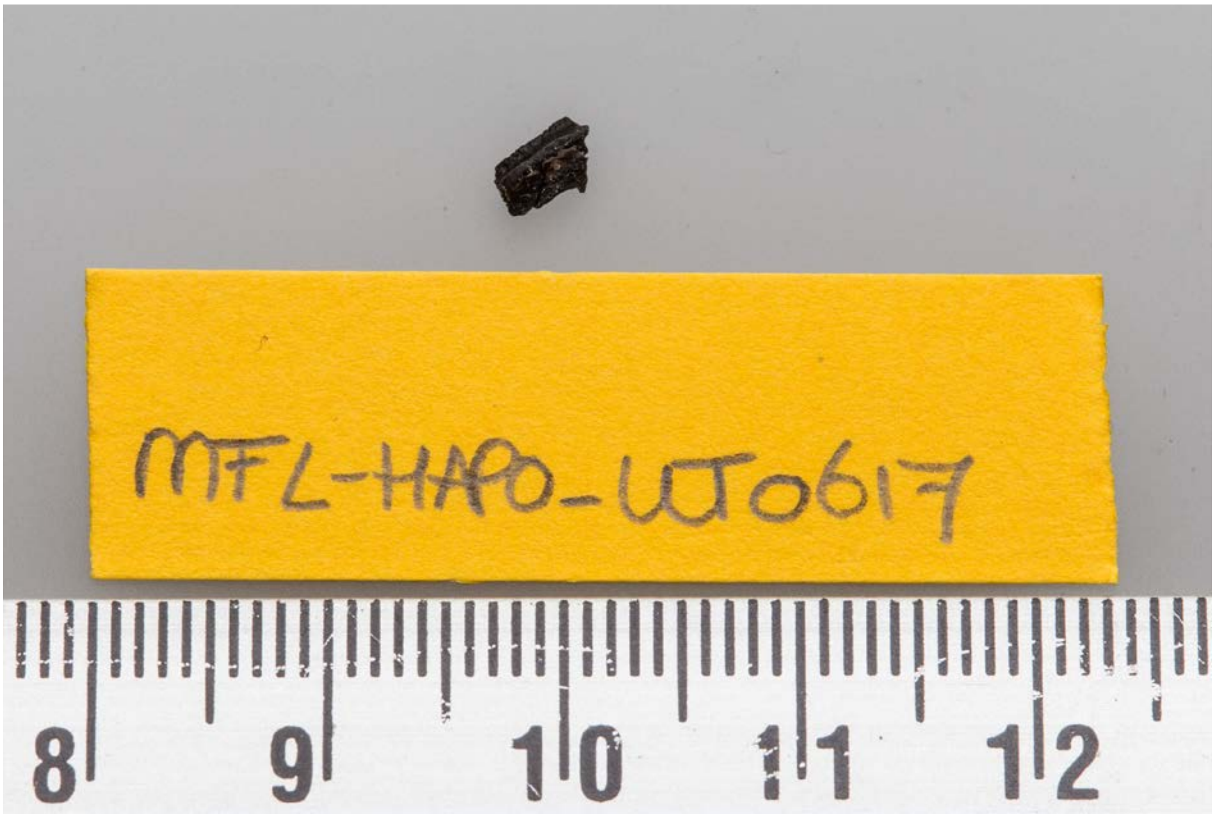

Fragment No ID (PA 71%) Mass 0.0111 g

MFL-HAPO-UT0674

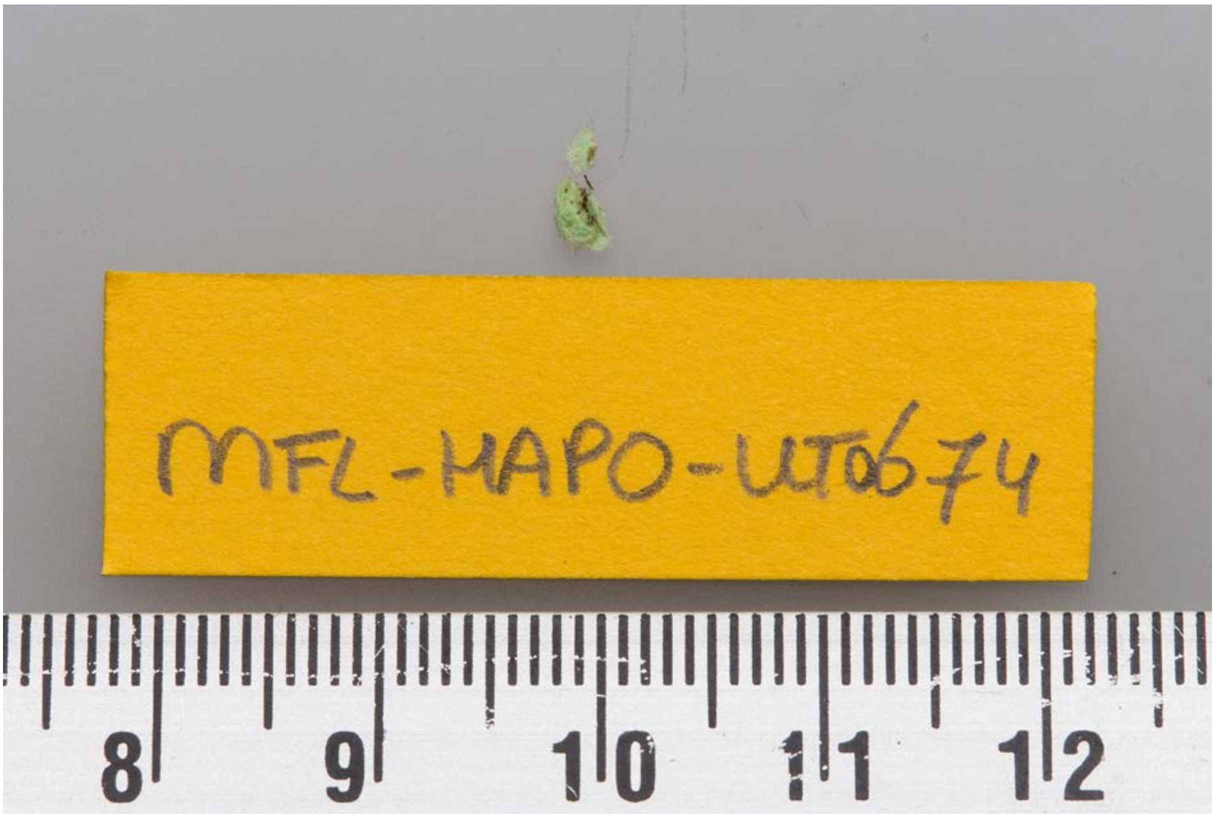

Rubbish: Paper (PI 23%), no plastics present Mass 0.0001 g

MFL-HAPO-UT0687

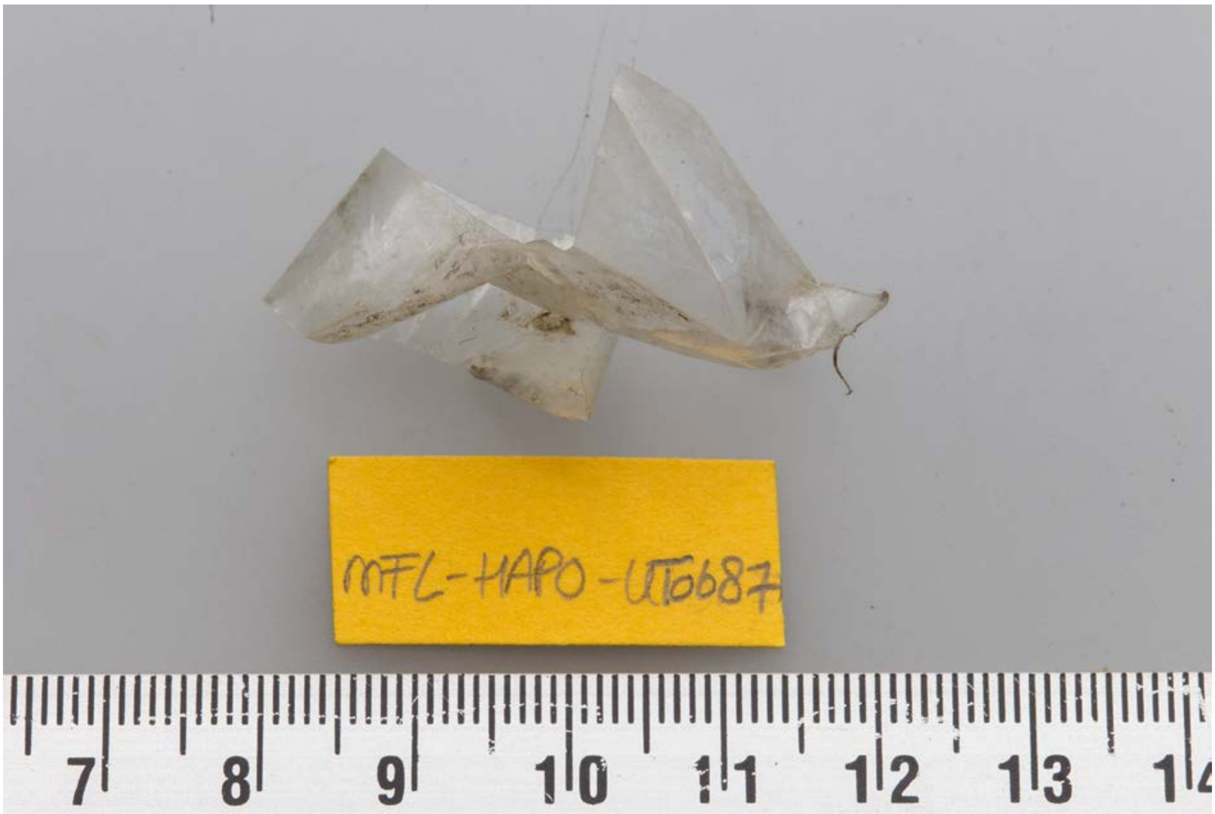

Sheet PP (93%) Mass 0.0396 g

MFL-HAPO-UT0714

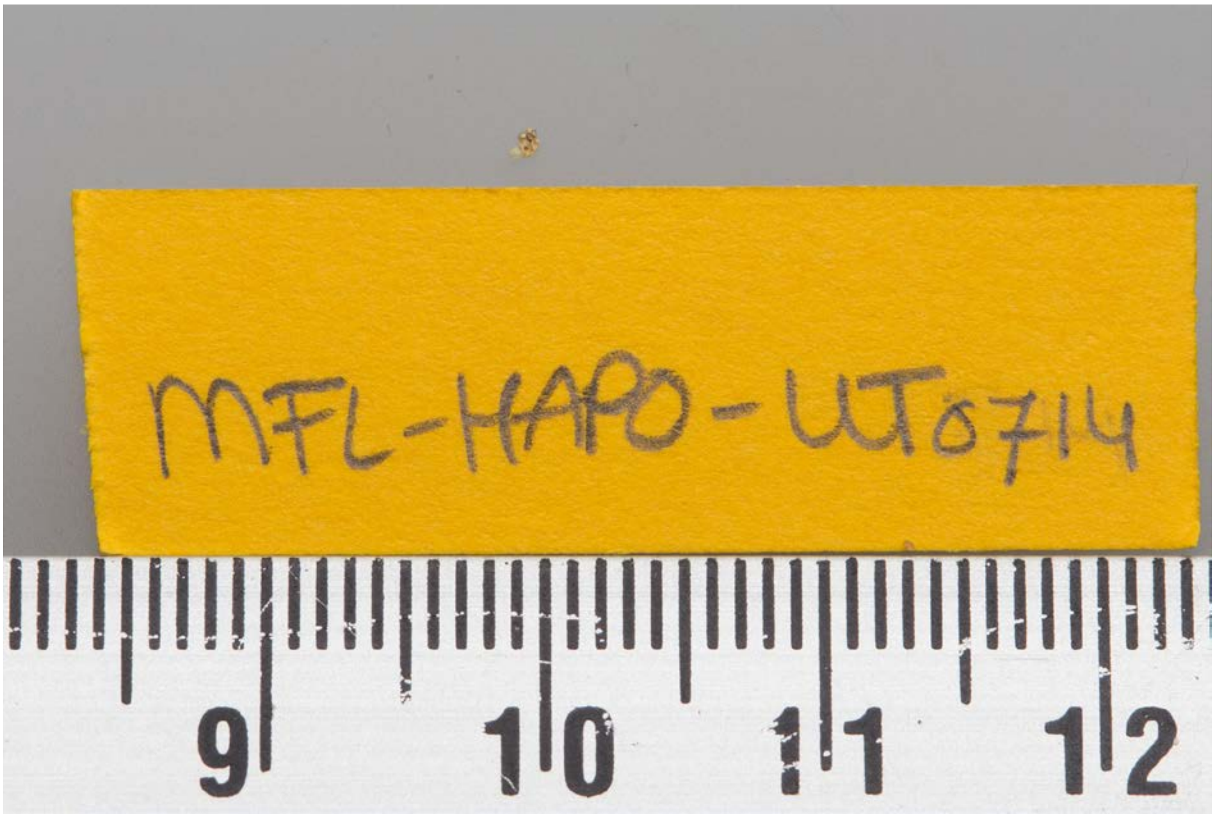

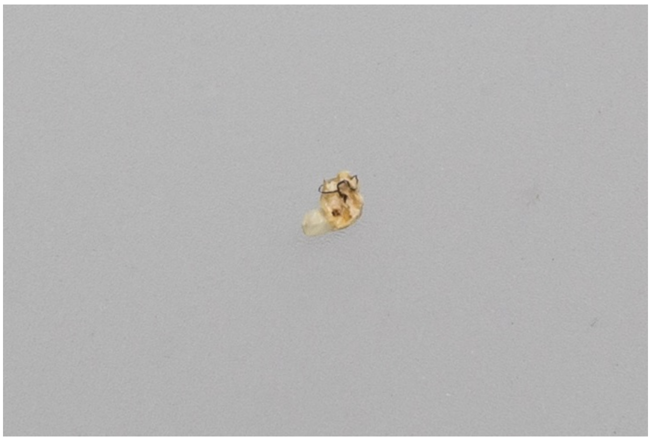

No plastic detected during the diet overflow procedure; this item found in the sieved remains.

Foam No ID (PA 60%) Mass 0.0001 g

MFL-HAPO-UT0799

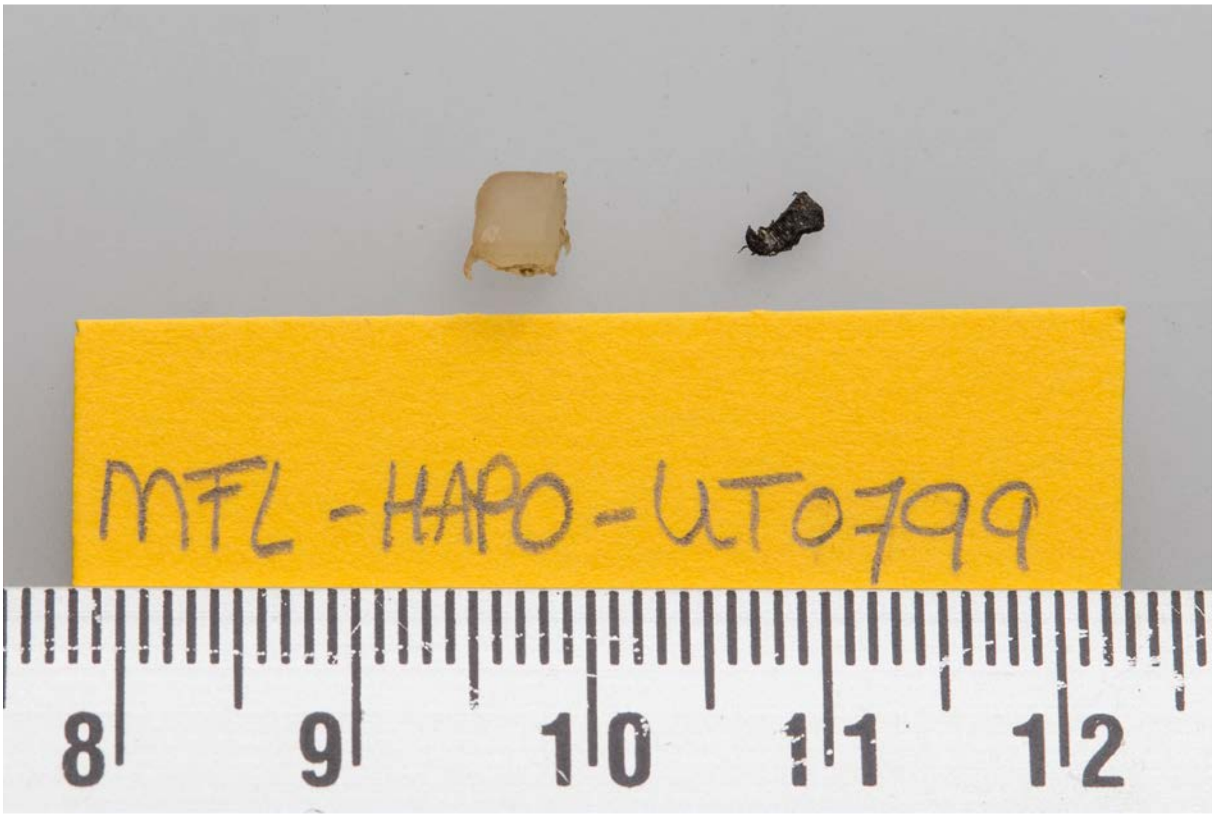

No plastics detected during the diet overflow procedure; these items found in the sieved remains.

Industrial Pellet (left) PE (99%) Mass 0.0328 g;

Fragment (right) No ID (PS 31%) Mass 0.0016 g

MFL-HAPO-UT0867

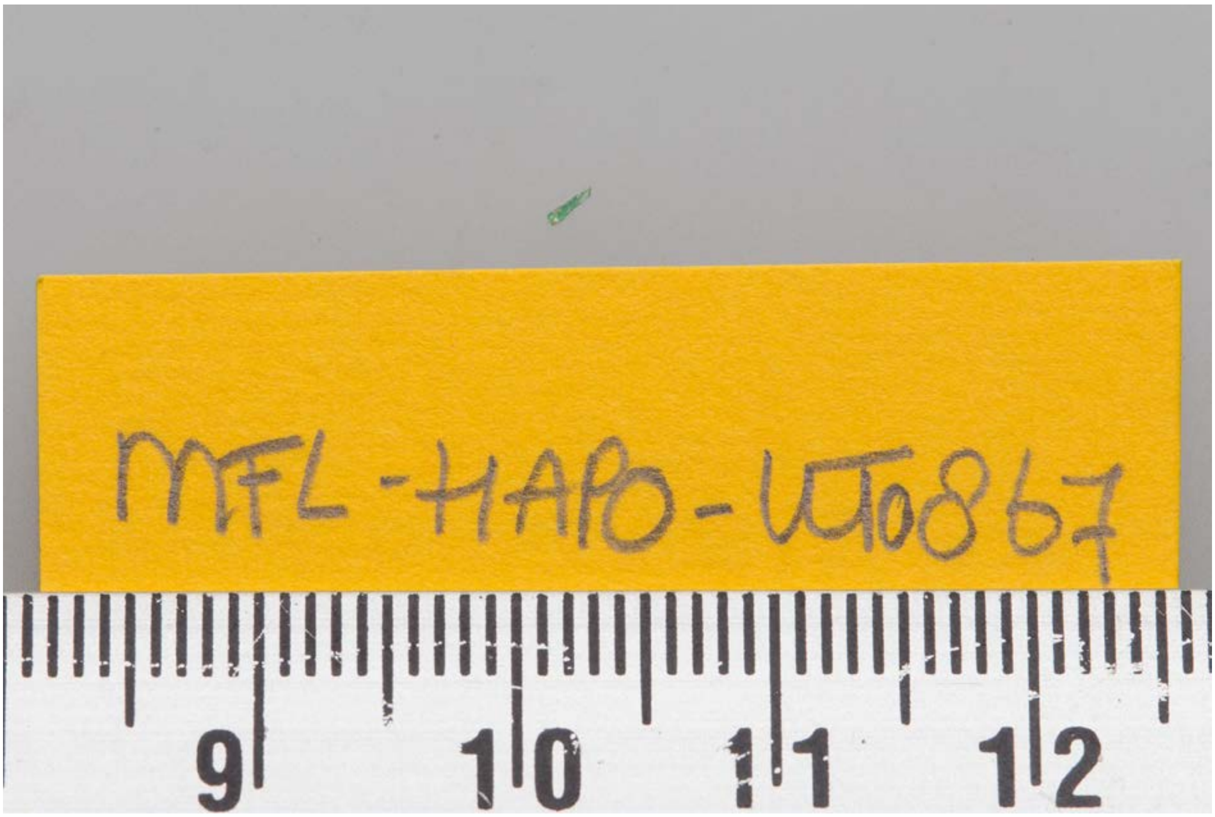

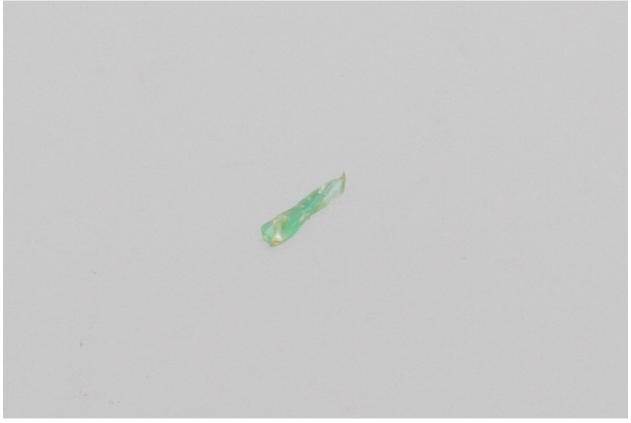

Fragment PE (92%) Mass 0.0001 g

MFL-HAPO-UT0932

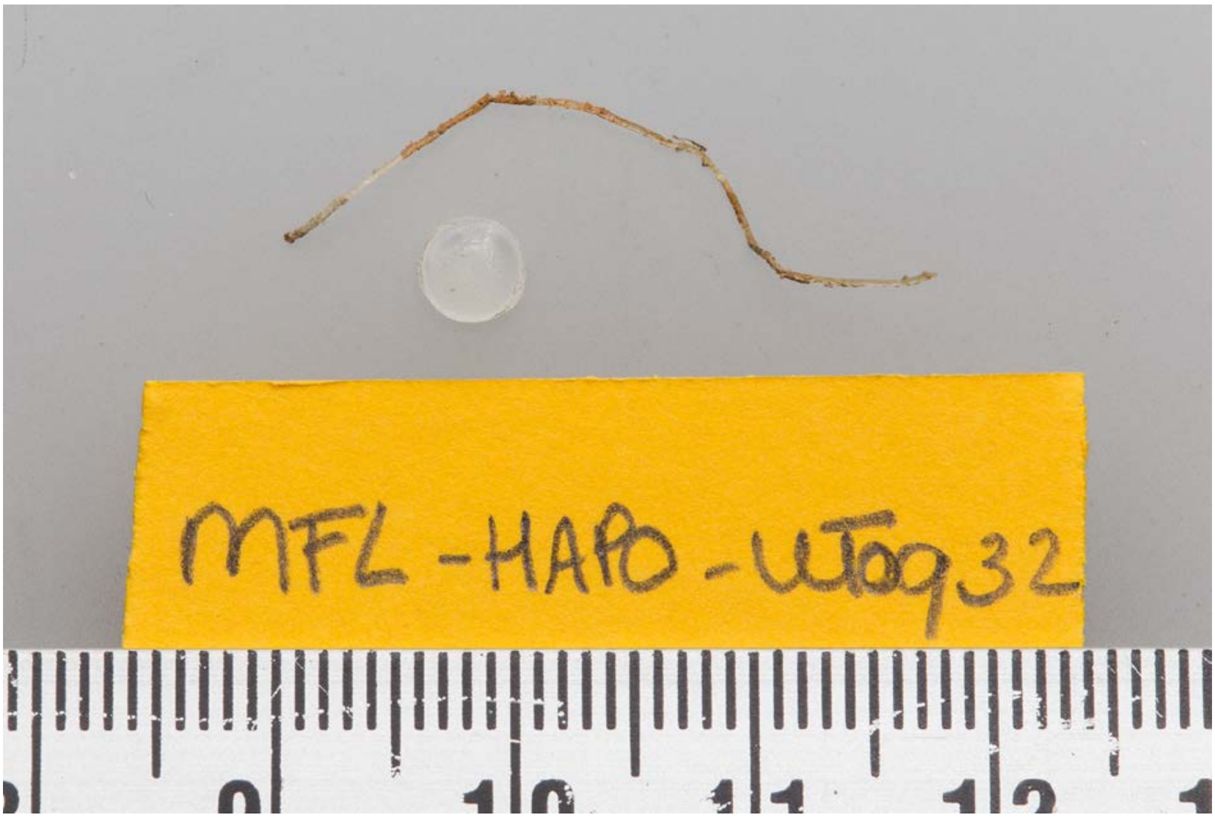

No plastics detected during the diet overflow procedure; these items were found in the sieved remains.

Thread (top) No ID (POM 69%) Mass 0.0024 g;

Industrial Pellet (Bottom) PE (97%) Mass 0.0131 g

MFL-HAPO-UT0945

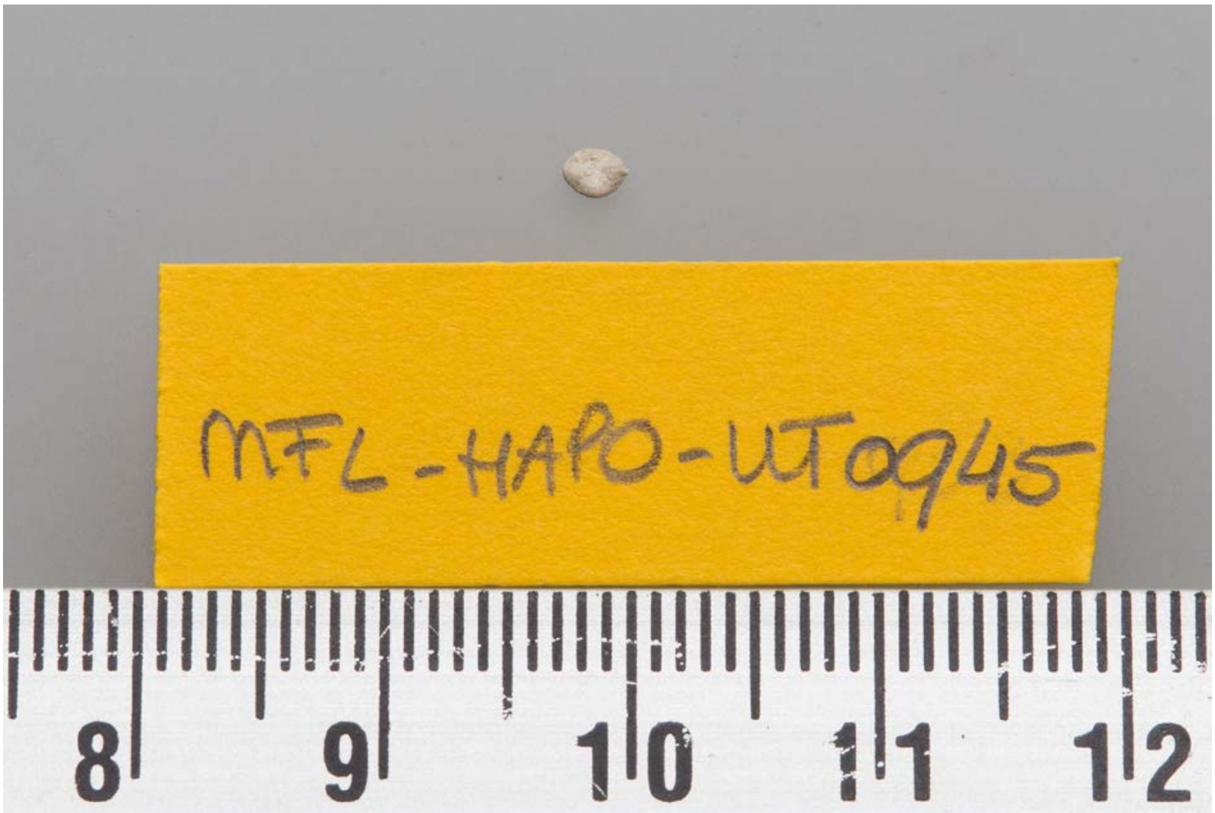

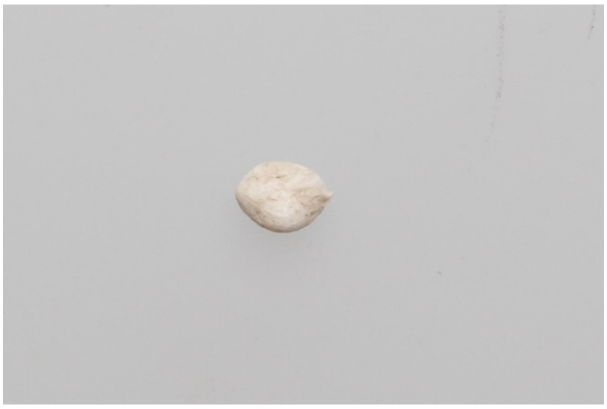

No plastic detected during the diet overflow procedure; this item found in the sieved remains.

Foam No ID (PS 74%) Mass 0.0006g

MFL-HAPO-UT0974

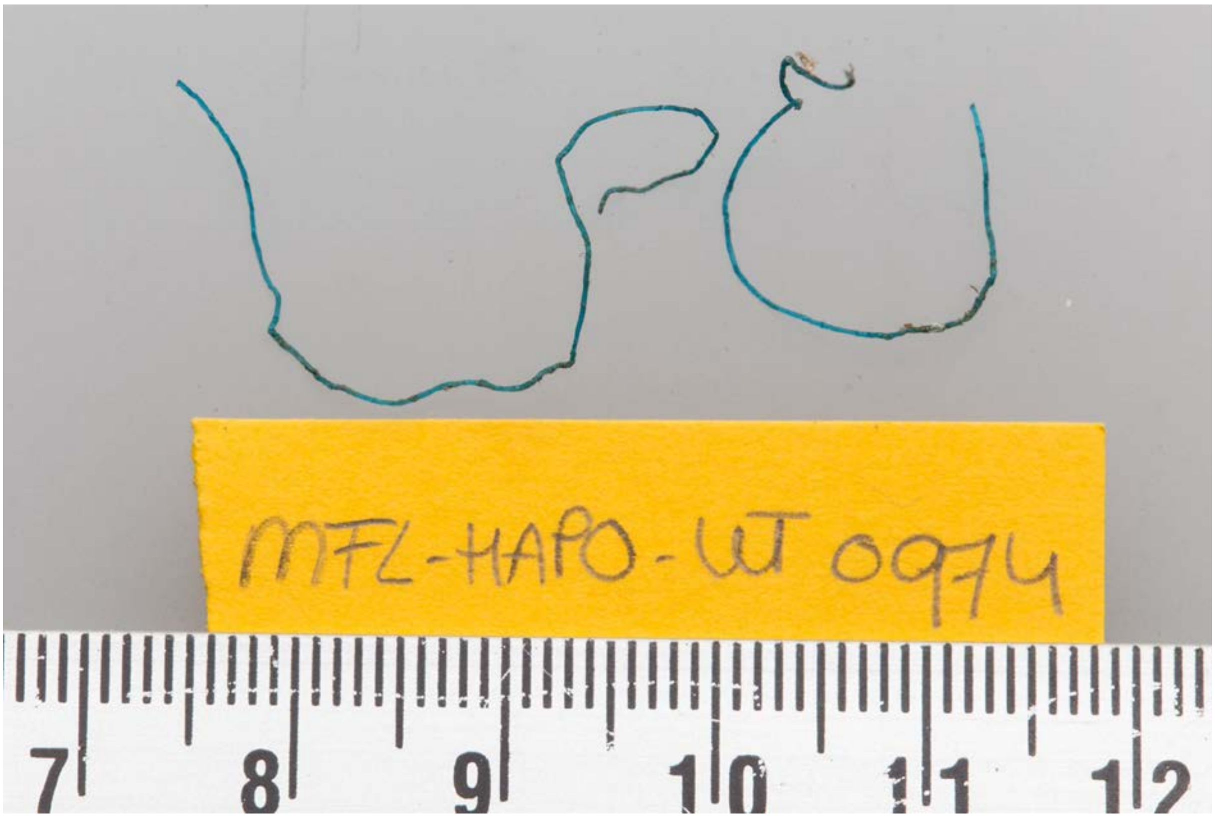

Left thread No ID (PI 48%) Mass 0.0030 g;

right thread No ID (PMMA 51%) Mass 0.0020 g

MFL-HAPO-UT0983

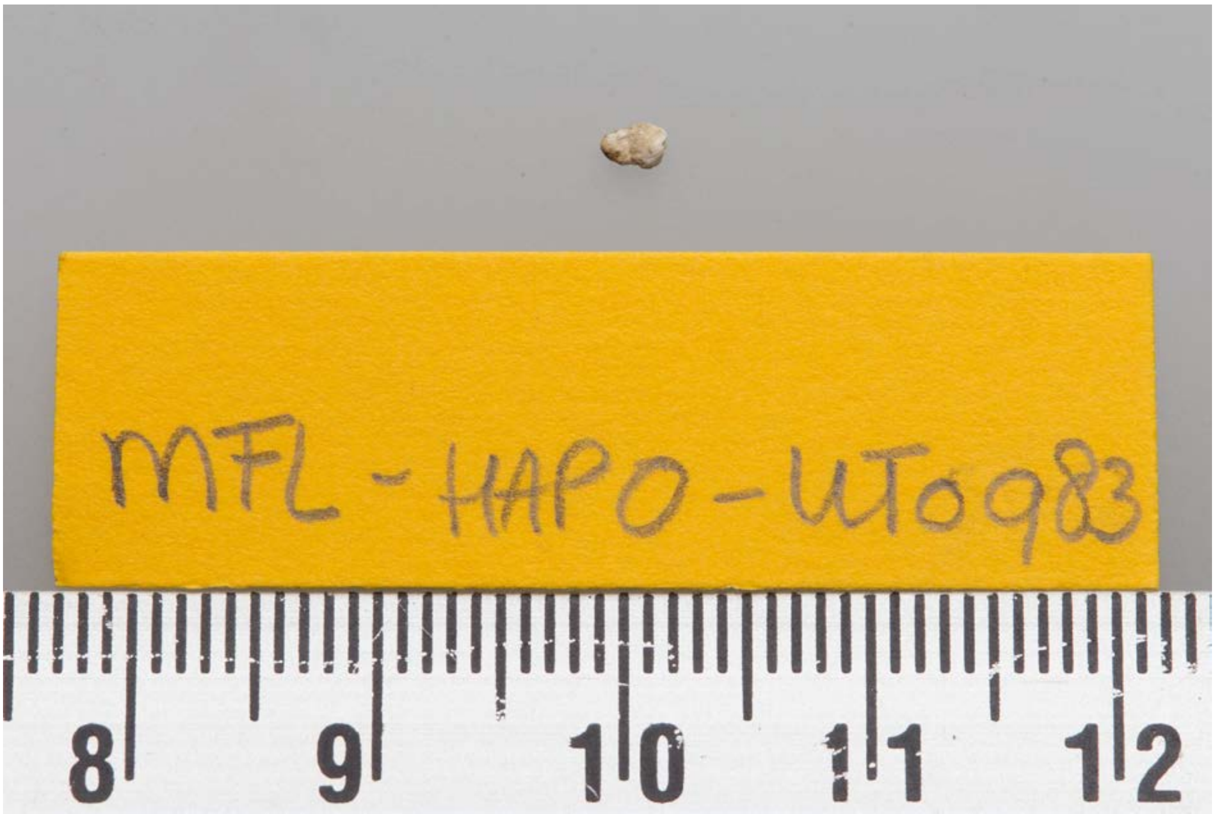

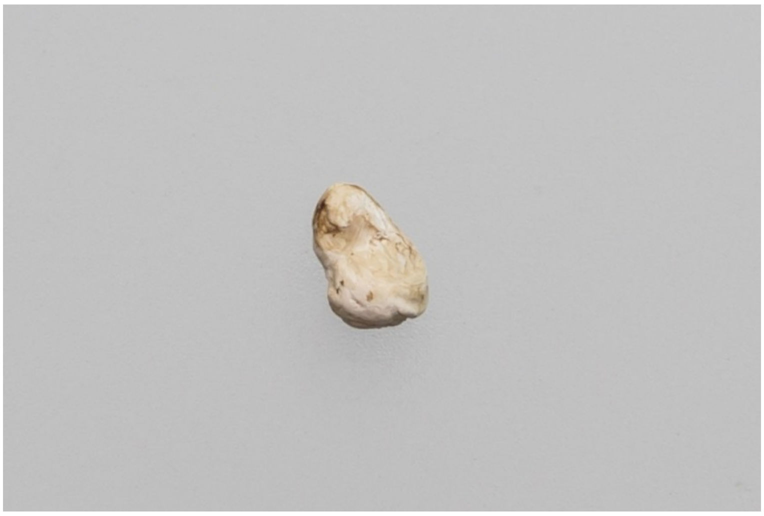

No plastic detected during the diet overflow procedure; this item found in the sieved remains.

Fragment No ID (PC 51%) Mass 0.0031 g
